# Periscope

**Published:** 1832-04-01

**Authors:** 


### Periscope;

OR,

### CIRCUMSPECTIVE REVIEW.

" Ore trahit quodcunque potest, atque addit acervo."

I.

I. A Grammatical Introduction to the London Pharmacopœia and Preface; or the Student's Self-Instructor, &c. By S. F. Leach, 1828.

II. Selections from Gregory's Conspectus Medicinæ Theoreticæ, and Celsus de Medicina, &c. for the Use of Medical Students, &c. By S. F. Leach, 1831.

III. A Literal Translation of those parts of Gregory's Conspectus and Celsus which have been fixed upon by the Court of Apothecaries for the Examination of Candidates. By S. F. Leach, 1831.

What is the object of the Court of Apothecaries in examining candidates in Gregory and Celsus? They will answer, of course, " in order to ascertain the candidates' proficiency in the Latin language." The Court could not take a more effectual method of defeating their object, than by specifying the portions of the works in which the youth is to be examined,—or indeed by specifying any work. The specification in question leads to the study of only the parts pointed out—and hence it is that we have literal and other translations of those portions, so managed and " made easy," that a sharp pupil may in a month or two get them all by heart, yet knowing hardly any thing of the language in which he is examined! This system is much worse than that adopted at the College of Physicians; for there the works of Celsus and Sydenham lie on the table, and the candidate knows not what page may be opened for his translation. Besides, the examination itself is in the Latin language, which affords some farther security against want of classical education.

The author of the works, at the head of this article, has done all in his power to facilitate the object of the candidate for a license at Apothecaries' Hall.

" I now present to your notice a literal translation of that portion of the Latin language, which has been selected, by legal authority, for the preliminary examination of candidates in the medical profession. The student desirous of self-instruction may confidently anticipate efficient assistance; and both teacher and pupil may calculate upon the diminution of labour, and the economy of time. Use freely what is here offered; but depend upon the *security* of your object *only* in a thorough knowledge of the rudiments and structure of the language you are required to understand."—*Pref.*

Mr. Leach is too well acquainted with the actual state of things, not to be aware that most of the candidates in question will attend but very little indeed to the *latter* part of this advice; and that they will be very ready to accept all the advantages of diminution of labour and economy of time, which are promised in the beginning of the passage. We blame not Mr. Leach. The Court of Examiners have, as it were, advertised a contract for the cheapest latinity of a candidate—and if Mr. Leach has been a successful bidder, the more fortunate he! Of all the translations of Celsus and Gregory, Mr. Leach's is the most literal—indeed it supersedes the dictionary in most instances. We shall introduce a specimen, by which the eader will be enabled to judge.

"As agriculture *promises* (promittit 31) aliments to healthy bodies, so medicine promises health to sick *bodies*. This *medicine* never indeed is not (Hæc nusquam quidem non est, this every where exists): since even the most unskilful nations have known (noverunt, *nosco*) plants and other *things* efficacious (prompta) in the assistance of wounds and of diseases. However it was cultivated among the Greeks a little earlier (aliquanto magis 19) than among other nations; and not even among those, from the first origin, but a few ages before us; inasmuch as Æsculapius may be celebrated the most ancient author. Who, because he cultivated this science as yet rude and common, a little more acutely (paulo subtilius 19) was received (receptus est, *recipio* 71) into the number of the gods: then his two sons, Podalirius and Machaon, having followed (secuti 45) the General Agamemnon to the Trojan war, brought (attulerunt, *affero* 31) no moderate (considerable) assistance to their fellow-soldiers."

In the preface to the second work at the head of this list (the Selections) the author cautions the student against the plan of committing to memory literal translations, or "interlineary versions," which plan would consume more time than would be necessary for obtaining a thorough acquaintance with the authors selected for examination, besides being attended with no small degree of *uncertainty*. In the following passage we entirely agree.

"The Editor of this little Work strongly cautions the Student from relying upon any of the facilitating Methods of Instruction so speciously advocated in the present day. An experience of more than twenty years sanctions the assertion, that the only legitimate, successful mode of learning a language, is by the medium of the rudiments. Let the declensions and conjugations be well fixed in the memory, and the structure of a language may then be explained, and readily understood."

We may remark that these two volumes are, one the Latin text, the other the translation of Gregory and Celsus, and are, no doubt, well calculated to facilitate the student's labours and acquirements in this department. We nevertheless object to the system adopted by the Court of Examiners, as leading necessarily to these royal roads to latinity.

The Grammatical Introduction to the London Pharmacopœia is free from all objections, and is a very useful little book for smoothing the crabbed technicalities of that Alcoran of our therapeutical faith.

---

II.

CASES OF ILIAC ANEURISM SUCCESSFULLY TREATED BY OPERATION.

THE operation of tying the external iliac artery for aneurism of the femoral, or of itself near the femoral, has been highly successful. Theory appears to have offered a true explanation of the fact, in the space presented by the vessel for the formation of an efficient coagulum, and if this be not the whole truth, it is a very material part of it. Two additional cases of this description have been recently placed on record, and as the operation, on each occasion, was performed in the theatre of a metropolitan hospital, the particulars are free from suspicion of colouring. The first case to which we shall refer, occurred to Mr. Bransby Cooper; both are reported in the London Medical and Physical Journal for January of the present year.

*Case* 1. Hans Jacobs, æt. 44, a Dane, a sailor, who in 1809 was in the Naval Hospital at Deal, with dysentery, and in 1825 in hospital at Bombay with a fever, but who had since been in good health, was admitted into Guy's Hospital, 16th July, 1831, under the care of Mr. B. Cooper. Below Poupart's ligament, towards the inner part of the left thigh was situated a large pulsating tumour, of an oval figure, the long axis extending in a horizontal direction, being rather broader from side to side, than from above to below. The circumference was solid; the central part prominent, and indicating fluid; tense; admitting of being considerably lessened in size by pressure; but again enlarging and extending upwards over Poupart's ligament. The skin was not discoloured, but traversed by large veins, which extended round the limb towards the hip. The whole limb was swollen and enlarged, the temperature increased, the veins congested, and the leg œdematous. His health was impaired; pulse 66, natural; countenance sallow, anxious.

Whilst working at the pumps on ship-board on the 2d April, he suddenly felt, without using any extraordinary exertion, a sensation in his left thigh, "as if the limb had been taken clean off by a shot," attended with severe pain in the lower part of the thigh, calf, and ankle. Four or five days after this the swelling commenced, when the pain diminished, but was reproduced on his attempting to stand on the limb. For two months the tumour increased little, during the last three weeks it had done so with rapidity, and been accompanied with severe pain, especially about the knee-joint, as well as on the outer part of the thigh. Some aperient medicine was given, and on the 19th the following operation was performed by Mr. Bransby Cooper.

*Operation.* "The patient was placed on a convenient table in a horizontal position: an incision was made, commencing half an inch above and to the outer side of the external abdominal ring, and extending outwards to within an inch of the anterior superior spinous process of the ileum, in a slightly crescentic direction, the convexity of which was downwards towards Poupart's ligament, and the concavity upwards towards the abdomen. The object to be obtained by this incision was to expose the tendon of the external oblique muscle, but in this case it was so covered with adeps that it required several incisions before it could be completely cleared. This done, by the next incision the tendon of the external oblique was divided precisely in a direction corresponding with the course of the first incision. This semilunar edge of the tendon of the external oblique was now lifted up by an assistant, in order to expose the internal ring; but, from the great development of the muscular fibres of the internal oblique, where they arise from Poupart's ligament, this object could not be effected, the ring being completely concealed by them: it was, therefore, necessary to pass a director underneath these muscular fibres, and separate them from their origin by means of a blunt-pointed bistoury.

The spermatic cord and internal ring were now exposed. The spermatic cord was then drawn upwards and inwards by an assistant; the fore-finger of the right hand was passed into the internal ring, in order to separate the peritoneum from the iliac vessels, by pressing it upwards into the abdomen. The fascia which connects the iliac vein to the artery on the inner side was next separated, in order to make a free passage for the aneurismal needle, which was then passed from within outwards around the artery, armed with a silk ligature, of double the usual size.

The object in using a large ligature was to prevent a too quick separation before so large an artery is properly secured by the adhesions on its internal coat. From some experiments, I have found that a very fine ligature will ulcerate a large vessel so quickly, that it will sometimes cause, and at all times lead to the danger of after-hemorrhage.

Before the ligature was tightened, the artery was brought to view; a precaution which in this instance was particularly necessary, as a small branch of the external spermatic nerve was seen taking its course along the front of the vessel, and this had to be carefully separated, to prevent its being included in the ligature, which was afterwards tightened. The pulsation in the aneurism immediately ceased, and the size of the tumour lessened at least one third: the edges of the wound were then brought together; one suture was employed to maintain their apposition, and the remaineder was secured with adhesive plaster and a dossil of lint. The operation lasted twelve minutes from the first incision to its conclusion."

Little in the after-symptoms or after-treatment requires particularization. Under warm flannels and gentle friction the temperature of the limb was maintained, or nearly so—distressing pain in the thigh was relieved by opiates—subsequent symptoms of local and general irritation by fomentations, opiates, aperients. On the day after the operation the tumour was merely half the size it had been previous to the operation—on that day the patient was allowed some white wine, and on the third day a mutton-chop—on the twenty-second day the ligature came away. After this the wound healed, and health is re-established. It deserves remark and attention that, when convalescent, the patient was attacked with violent inflammation in the right testicle, and subsequently with inflammation, less acute, of the left. The phenomena are to be explained, we presume, by the irritation and implication of the spermatic cord, and the continuity between the vasa deferentia. The case is highly satisfactory, and creditable to Mr. Cooper.

*Case* 2. The following is related by Mr. Guthrie, and formed the material of a clinical lecture to his pupils. We are happy to perceive that this able and eminent surgeon is frequently in the habit of communicating to the public his observations and experience. If all of the same standing made their opportunities as available to others, as Mr. Guthrie endeavours to make his, it would neither be amiss for science nor themselves. But we have done.

John Hicks, æt. 28, the subject of the case, presented an aneurism, situated above Poupart's ligament, which was raised by the lower part of the tumor, and by its indentation seemed to divide it into a large and a small portion, the former below, the latter above, and appearing to occupy a space equal to the lower half of the external iliac artery; it was as large and as prominent as a good sized orange: the pulsation very strong and distinct. There was a slight bellows-sound in the heart, and though the patient was bled thrice in the month preceding the operation, the pulse remained generally above ninety, and sharp. The aneurism as far as he could recollect or judge, had taken place from a strain. The situation and size of the tumor rendering it probable that the greater part of the external iliac, if not the whole, might be unsound, Mr. Guthrie decided on performing the operation so as, if necessary, to place the ligature on the common trunk of the right iliac arteries. We cannot do justice to the operator, but by transcribing his own description of the operation.

*Operation.* "I began by making an incision in the side of the abdomen, extending from about an inch within the ninth rib, and about one inch above the level of the umbilicus, to nearly an inch within the ileum. This incision was six inches long, and the integuments, superficial fascia, and the external oblique tendon, were divided as rapidly as possible, until the fibres of the internal oblique were fairly exposed: these, being of uncertain thickness, were next divided cautiously, until the tendinous expansion of the transversalis was brought into view, going to form the sheath of the rectus. The lower portion of this was muscular, but I divided the tendon immediately above this part very easily, introduced a director upwards and downwards, and cut the transversalis upon it in both directions. The peritoneum was now exposed, covered, however, still by the fascia transversalis, which in places fastened and bound it down so firmly as to require it to be divided with greater caution by the knife and director. This obstacle being removed, the peritoneum rose and fell with the intestines on each motion of the muscles of the belly, shewing how carefully the preceding steps of the operation must be done to avoid doing it an injury, which at this part might be fatal. I now separated the perineum from the fascia transversalis and transversalis muscle, and endeavoured to pass my hand behind towards the spine, between the ribs and the ileum; but I found there was not quite room for it to pass easily: I therefore enlarged the incision as much downwards as the proximity of the aneurism would permit, and divided the transversalis tendon upwards for about half an inch more. As it was here very firmly passing across, I raised the part to be divided on my two forefingers, and desired an assistant to divide it with a pair of blunt scissors: in doing this, a small fold of *peritoneum* was caught and divided, to the extent of near a quarter of an inch. It was, however, out of the way, and my hand could now pass with perfect ease, although it required to be done with the greatest caution, raising and separating the peritoneum before it, and passing over the psoas muscle, until my forefinger rested on the common iliac. It was only at this part of the operation that assistance could be given by any one: Mr. White now, however, took charge of the bag of peritoneum containing the intestines, and raised it and them sufficiently to enable me to see the point of my finger resting on the vessel and on the bone beneath. I could now feel, and indeed see, the common iliac and its bifurcation into the internal and external iliacs, and it was as easy to have placed a ligature on any one as on the other of these three arteries, and more particularly on the common trunk. The external iliac appearing, however, to be sound, and of its natural size and appearance, an inch below the bifurcation, I cleared it with a blunt knife, and passed a ligature around it from within outwards, made of two strong threads of dentist's silk, well waxed. I then cut away one end, laid it straight, and allowed the peritoneum and intestines to fall back into their places. If I had a doubt about the soundness of the external iliac, I should have tied the common trunk: but as it was, and as I could with propriety place the ligature an inch below the bifurcation, I did it, and of course gave my patient an additional chance of escaping mortification.

The wound was first brought together by three strong ligatures passed through the integuments, leaving the muscular wall to itself; and as these did not close the wound, four stitches through the skin, of a single thread each, were added in the interstices, which brought the edges of the whole line of incision into contact. The adhesive straps were then applied, and a compress was retained by other straps and a flannel roller. The legs were both raised by a pillow under the knees, the body bent, and the patient inclined towards the affected side."

Mr. Guthrie remarks, en passant, that the peritoneum which was exposed and accidentally wounded had a reddish colour and slight roughness of its external surface, which readily distinguished it from the leaden colour of the peritoneum covering the intestine. We pass to the events that succeeded the operation.

"The temperature of the limb previously to the operation, on Satuday the 19th of November, was 102; the pulse 92. On being put to bed, a hot bath was applied to the foot, and two persons were directed to rub the foot, leg, and thigh, constantly under the bedclothes, which they did until midnight. At 8, p. m. the pulse was 106, the temperature 92. At twelve, on Sunday, the pulsation of the femoral artery was distinguishable in the middle of the thigh, and he was free from pain in the limb, which I considered to be a most important sign of safety from mortification; for you may lay it down as a general rule, that, when a limb is going to mortify after such an operation, there is always great pain in the heel, calf, and even in the thigh, whilst at the same time there will be a mottled appearance of the skin, which together are the forerunners of an inevitable and generally fatal mortification. In the evening, the pulsation of the posterior tibial artery was just discoverable at the heel; and on the Monday, or forty-eight hours after the operation, I considered the danger of mortification to have passed by, and that of inflammation to have commenced. The pulse rose to 134, was jerking, but neither full nor hard. I had him bled to nine ounces, when the pulse diminished in volume nearly three fourths, and he felt himself relieved from an oppression he could not describe before. The abdomen was a little swelled and tympanitic, but not painful any where on a fair degree of pressure,

An enema, with Ol. Ricini, was given, and repeated with Ol. Terebinth., with full effect. In the evening the pulse was 140, and very peculiar, there being seventy strong beats, and seventy short feebler ones following them; the countenance a little anxious. I had him bled, with my finger on the pulse, until twenty-two ounces were drawn, when he became faint, a profuse perspiration came over him, and the limb lost in temperature."

Mr. Guthrie was anxious to avoid syncope, and did avoid it, but during the eight subsequent days 107 ounces of blood were abstracted, being cupped, buffed, and firm till the last. The man also took colchicum and digitalis till the pulse intermitted, was limited to four ozs. of bread and tea daily for the first fortnight, to eight ozs. with a pint of milk for the second. The ligature came away on the 28th day. On the 13th December, the tumour was evidently softer, and increasing towards the line of incision, into which it emptied itself next day, with diminution of size; it still continues to discharge slightly. The man now takes good diet, and, as far as the operation is concerned, it has succeeded. Mr. G. observes that, during the operation, he laid bare one inch of the common iliac artery, and he felt confident that he could, with ease, have placed a ligature on the aorta, by a farther extension of the external wound for half an inch. Mr. Crampton, we believe, has made an observation of a similar description. In the present instance, Mr. G. attributes his success to the extent of his incision. Mr. North, who saw the operation, speaks highly of the coolness and manual dexterity with which it was performed.

We direct attention to one striking point of contrast between the case of Mr. Cooper and this. In the former, wine was given on the day succeeding the operation, and the patient may be said to have been supported; in the latter, depletion was used boldly and antiphlogistic regimen severely; yet both individuals did well. But Mr. Cooper's patient was more advanced in age, had suffered sickness, and probably been intemperate—debilitating circumstances; whilst Mr. Guthrie's patient was young, and apparently vigorous. We regret the absence of particulars respecting the manner in which the disease commenced; being ignorant of this, we can form no conjecture on the ultimate event. In another part of this Number, will be found the particulars of a case of aneurism of the axillary artery, for which the subclavian was unsuccessfully tied by Mr. Brodie.

---

### III.

### CASE OF FUNGUS CEREBRI, WITH THE DISSECTION.*

FUNGUS of the brain, after injuries or operations, is an affection that has excited some attention, much perplexity, and is still involved in a great degree of obscurity. The explanations hitherto offered of its nature are unsatisfactory, the actual condition of organization or disorganization which attends it unknown, its treatment dubious. We are reduced to the collection of facts to determine the truth, if the truth is to be determined, on these several questions. We, therefore, notice the following cases, related by Dr. Armour.

*Case.* William Park, aged 15, was admitted into the Glasgow Royal Infirmary on the 29th Nov. 1816, at 9, p.m. At this time, Dr. Armour was surgeon's clerk, received, and subsequently watched him. At 3 o'clock in the afternoon, he had received a kick from a horse on the frontal bone, immediately above the right eye. A large depression of the bone was produced; he was stunned for a little while, but soon recovered his senses and recollection. He was bled to ℥x. previous to which the pulse was 120, and immediately afterwards fell to 90. Some time afterwards he vomited slightly, the pupils were dilated, and he had two or three convulsive fits. On admission, the bone was found

---

* Glasgow Journal, No. 16. Nov. 1831.

depressed to a considerable extent, broken into many fragments, with some cerebral substance lying on their surface. A piece of the adjacent bone was removed by the trephine, the fragments taken away, and the fracture found to extend downwards exactly to the orbital process of the frontal bone; the dura mater and other membranes were lacerated to the extent of an inch and a half, and spicula of bone driven into the brain. These were removed; a coagulum then occupied the centre of the wound; the edges were brought together by stitches; straps applied, with an aperture for blood to escape, and a piece of lint and a handkerchief placed over all. *V.S. ad* ℥xv. He moaned occasionally, but was quiet, and at 11 p.m. answered quite collectedly, and said he had little pain in his head. *Dose of calomel and rhubarb, to be repeated in two hours, and in the morning if necessary.*

30th. At 5, a.m. he complained of some pain in the head, with a sharp pulse. He was bled to ℥xij. after which he had a fit but immediately recovered, and slept. At 8, a.m. he had had two stools—pulse 100, firm. *Rep. bolus.* At 10, p.m. *V.S. ad* ℥ix. He became faint and vomited. At 3, p.m. the pulse was 120, sharp; skin dry and warm. *V.S. ad* ℥xij. *Liq. amm. acet.* ℥ss. *o. ⅟ horâ.* After losing the blood he was seized with convulsions, which soon ceased, but left him delirious, and very restless. The dressings were removed; the wound adhered where its edges were placed in contact, and a coagulum was formed over its centre. *Rep. boli* ij. *Hirud.* x. *temp. dextro. Vesicat. nuchæ.*

Dec. 1. The restlessness left him after the last bleeding; to-day the countenance is healthy, pulse 124, soft. *Hirud.* x. *tempor.* 1, p.m. Complains; pulse 440. Wound looks well, coagulum has come off, and laceration of dura mater exposed; already an attempt at granulation on the surface, but at one point a small projection, apparently cerebral, of about the size of a common bean. *Mag. sulph.* ℥ss. *p. r. n. repetend.*

Dec. 2. The cerebral tumor has enlarged astonishingly: it is of the size of a pigeon's egg, and is distinctly seen passing out from the wound in the dura mater. It was handled without occasioning any uneasiness, and was torn away on a level with the cranial bones, without any perceptible effect. Health improved—pulse nearly natural—expression good—sleeps naturally.

On the 4th, the tumour had increased very much in size, and the health was not so good. The fungus had been shaved off without success, and the surgeon now began to dig it out from considerably lower than the level of the cranium, and, allowing the blood to coagulate, was in hopes that an organizing action might take place between it and the cut surface of the brain, and that granulations would form. Any reason is better than none, but a good one is better than a bad. The practice was unsuccessful, for the coagulum having remained in situ for two days, was forced off by the fungus, which increased in spite of pressure, and the brain exhibited no healthy appearance. It was again shaved off by the scalpel without obvious benefit or injury. Powerful pressure was now resorted to, but symptoms of compression of the brain shewed themselves, and were removable only by the removal of the pressure. Caustics (argent. nitrat.) were applied, the bandages being only removed when symptoms of pressure were actually present; this plan was continued till the 20th day from the appearance of the fungus without benefit. The boy had hitherto been perfectly sensible; on the evening of that day he became insensible, convulsions came on during the night, and at 8 o'clock he died.

" *Inspection.* Before opening the head a very great difference was observed, betwixt the appearance of the fungus, and that which it had exhibited during life. Even to the last moment of the patient's existence, the cerebral matter was found, either protruding through the cranial opening, or on a level with it. Now, it had retired completely within the cranium, and the finger, when introduced, did not detect the existence of any such matter. On looking in at the opening, a deep cavity alone was observable. When the skullcap was sawed off, the chief, and most remarkable fact noticed, was, that the greater part of the substance of the right hemisphere was gone, its remaining surface being in a putrid-looking state. The substance of the brain, throughout, was softer and more pulpy than natural."

Dr. Armour makes some observations on the case, which we must pass by. He discusses the question of phrenology: it were well if he could first settle that of hernia cerebri. But we can find more room for facts than arguments, and the following are said to be facts.

"A man was brought into the Infirmary, a considerable time after the preceding, with extensive fracture and depression of the bone of the left side and fore part of the head, and injury of the membranes. The necessary operations were performed, and the fragments removed; the antiphlogistic treatment was adopted, as the symptoms seemed to require, although I do not remember that he was bled; on the contrary, as far as my memory bears me, he was not. After a time, a fungus made its appearance, which was treated by pressure and the application of burnt alum, which acted as an escharotic. The progress was bad, and ultimately the appearances became so formidable, that I well remember, the surgeon (not the one who treated the former case,) at the visit, stopt at the bed-side, gazed at the patient, who was apparently in a state of insensibility, and passed on. I asked, 'What is to be done.' His answer was, 'Let him alone, he is dying.' In the evening, his friends, also, who had come from the country, were so convinced of his approaching dissolution, that they came to my room, to ask me where they should go to order a coffin, and when I went to bed, I had not the slightest expectation that he should see the morning. When I visited the ward, next morning, after breakfast, I asked the nurse, when he died. She answered, 'He is not dead, he has sat up in bed and has taken breakfast.' A hint was taken from Nature—no farther pressure or caustics were applied; simple dressings were laid on, and when I left the Infirmary, for the Continent, to prosecute my studies, the cure was advancing, and the fungus gradually becoming more and more encroached upon, by the cicatrization of the adjacent parts. At my return, I was told he had left the house well as to the fungus, although, I think, it was added, that his mind was somewhat weakened; I have neither seen nor heard of him since. Another case, which subsequently occurred in the same hospital, which I did not see, but which was mentioned to me by the attending, and still a different surgeon, was the following. The frontal bone was smashed down in numerous fragments, which were mixed with the hair and brain. The bones, hair, and portions of the anterior lobes of the brain were removed, much cerebral matter was also subsequently effused and taken away with the dressings. Only simple dressings were employed, and the man recovered."

In one of these cases, the cessation of mechanical treatment certainly appears to have been succeeded by the cessation of the malady. But as mechanical treatment could not have produced it, and as, in many other cases, the fungus has proceeded without interference or with it, we must not jump to conclusions too hastily, or take one case for more than it is worth. We observe a circumstance of importance in the first case related by Dr. Armour. Bleeding, on four separate occasions, was followed by convulsions. This is a fact, and not an insulated one, to be remembered in the observation and treatment of the effects of injuries of the head. We make no further remarks on the subject at present.

---

### IV.

### ANEURISM.

[St. Thomas's Hospital.]

CASE 1.—*Aneurism of both Carotid Arteries—Operation on one Side—Result favourable.*

Peter Varner, æt. 65, applied at this hospital, April 7th, 1831, presenting an aneurismal tumour of the right carotid artery, at the point of its division, which was the subject of his complaints, and principally attracted the attention of the surgeon. His report is, that a few days after Christmas, he first noticed a small swelling, possessed of pulsation, not larger than a pea, in the above situation, having carried a heavy load on his head a short time before, when he thinks he might have strained some part of his neck, but he is not conscious of having then received any injury. This enlargement has been gradually increasing in size, accompanied by more serious symptoms within his head, such as throbbing on that side, augmented at intervals, vertigo and confusion. Deafness of the same side has existed from childhood, but, on the left, has subsequently come on, and he hears now very indistinctly. The aneurismal disease has attained the size of a large walnut, and the pulsation of it is very forcible; pressure upon it is productive of considerable pain, and gives rise to increase of the unpleasant sensations within the cranium. Its growth does not appear to have been more rapid latterly.

He is of unusually short stature, and consequently short-necked. His employment has been that of a porter. He is stated to have lived regularly, and, though pallid in the countenance, has not looks of ill-health for a man of his years. Upon examination with the stethoscope, the action of the heart is found natural, that of the arteria innominata preternaturally strong, and, in the tumour itself, bruit de soufflet is plainly audible at each beat of the vessel. The pulse, when the man is quiet, does not exceed 88, but it is readily excited, as, at the time he underwent more particular scrutiny by Mr. Green, on the 8th, it was raised to 110, and was much more powerful than naturally. Tongue rather white, and clean at the edges. Bowels regular. Appetite good. His nightly rest has been often disturbed. Is capable of walking a long distance. Attacks of palpitation have been frequent, but never syncope. There is no cough nor dyspnoea.

April 15th. We cannot perceive any alteration in the disease on the right side of the neck, excepting, perhaps, a slight decrease, probably from the state of quietude the patient has been preserved in; but, on the left, an incipient aneurism has been discovered in the corresponding portion of the carotid, small, and unattended by conspicuous or violent pulsation. Everything connected with the patient's bodily functions has been properly performed, and his mind is now prepared to endure the operation of tying the common carotid artery, which Mr. Green to-day accomplished. An incision, between two and three inches in length, was made on the inner side of the sterno-cleido-mastoideus muscle, and in the direction of its fibres, which commenced a short distance below the cricoid cartilage, and reached to the upper edge of the clavicle. The neck being remarkably short, accounts for the external wound not being longer. With a few gentle strokes of the knife, the muscular substance was brought into view, and here a superficial artery, which bled rather copiously, whereby the deeply-seated parts were obscured, was secured by ligature; then, by means of the sharply-edged handle of a scalpel, the cellular tissue was torn through, and, after some time, the sheath of the vessels was exposed, which lay far from the surface, the consequence of the shortness of this region. This was raised with the forceps and cut into horizontally; and, being in the next place further opened on a director, the carotid artery was more fully seen, but still, from the cause alluded to, not to the usual extent. Some branches of the n. descendens novi were visible on the external side of the sheath, but nothing of the great jugular vein nor par vagum internal to it. A large ligature was placed under the vessel, from without inwards, with a blunt-pointed needle, and tied firmly. One extremity was cut close to the knot, and the wound being cleaned, was dressed with adhesive plaster. Suspecting that the coats of the artery might not be in a perfectly sound state, Mr. Green chose a thick ligature. The effect of the latter upon the aneurismal tumour was a manifest and instantaneous diminution in size, and in the force of pulsation, which was yet distinguishable. But the patient having been carefully removed to bed, in about an hour this had ceased altogether.* In the space of thirty minutes, the man was conveyed from his ward, the operation performed, and he was put into bed.

15th, 10 *o'clock, p.m.* Within the last few hours, respiration has become hurried and laborious, and the pulse 120, strong and bounding. About 9 o'clock, a slight convulsive state came on, and recurred in a less degree in the course of half an hour, after the patient had been bled to ℥xx. by Mr. Green's order. He is now quiet, and the pulse much reduced in force, but not in frequency. Has taken nothing except barley-water, and has not felt great thirst.

16th, 12, *a.m.* Very little time has been passed in sleep, but the patient has endured no absolute pain, and complains not of uneasiness in the head; he has been restless, and is impressed with the belief that he shall die. Pulse now 120, rather full, and has regained considerable strength. Respiration has been frequent during the night and loud, and this state has not subsided—there is no cough nor flushing of the face. The inferior portion of the right carotid is seen and felt to beat with rather violent action, but no pulsating sensation is distinguished in the tumour, over which the integuments have become wrinkled and flaccid, nor in the temporal artery. A remarkable tenderness is exhibited on touching the part. In the great vessel on the opposite side, no increased force is perceptible; countenance undisturbed; no motion from the bowels; urine has passed freely.

12, *p.m.* Our patient is now asleep, but, having been unquiet, is lying with his head below the pillow—is breathing easily, and not louder than natural—pulse has not changed during the day. One alvine evacuation has been procured by a dose of ol. ricini. At 2 o'clock Mr. Green visited him, and ordered him to be seen every six hours by the dresser, and the slops to be continued.

17th. He has enjoyed sleep for some hours, and acknowledges no pain within the skull. Some redness has taken place around the incision, with swelling, and, at 9 this morning, nine leeches were applied to the part by the dresser, and a poultice of bread afterwards. Pulse 104, reduced in volume, and soft—respiration not oppressed—bowels moved once this morning. The redness and tumefaction surrounding the wound being but little abated, Mr. Green directed 12 leeches to be re-applied, with the poultices, and prescribed—

Hydr. submur. gr. ij. Ant. tart. gr. ss. Opii, gr. ss. M. 6tis horis.

In the evening, there was some difficulty of deglutition, which was painful, but unattended by cough. Some beef-tea was given, however, without inconvenience, as the patient had become low.

April 18th. His night has been tranquil, and the medicine exhibited regularly up to 6 o'clock, a.m. Pulse is 100, soft, and not fuller than the natural beat—respiration quite easy—bowels acted twice yesterday, but no stool has been to-day obtained. The poultice and strapping, which had become excessively foul from the discharge of pus and bleeding from the leech-bites, were removed from the wound, which is looking perfectly healthy, and is filled with granulations. Suppuration to a trifling extent has taken place therein, and the redness has nearly disappeared. A few short strips of plaister were put on as before, and Mr. G. ordered the beef-tea to be consumed according to circumstances. Pills of calomel, &c. to be omitted.

19th. About 9 o'clock last evening, he had some pain in the stomach, which, in a few minutes, was followed by strong efforts to vomit, but nothing was ejected. His bowels not having acted during the day, an enema was thrown up, and soon brought away a dark-coloured

* Some doubts are entertained upon this point, by those who watched the case as attentively as ourselves. We, however, carefully examined the part at the above time, in the presence of Mr. Green, who, to the best of our belief, holds our own opinion.

and very fetid stool, and the retching ceased. At 3, a.m. another evacuation of like character passed, and there was some little difficulty experienced in voiding his urine. This, however, endured but a short time. At 1, p.m. Mr. Green saw him, and, as some nausea remained, ordered—

Hydr. c. cretâ, gr. iij. Opii, gr. ss. b. d. Mist. sal. eff. ℥ij. 4tâ q. h.

The wound is looking healthy, and a free suppuration is established. A feeble pulsation has been distinguishable for the last day or two in the tumour, which has been gradually decreasing.

20th. Has passed a good night—had no return of sickness, nor of difficulty in expelling urine, and expresses himself as quite well. One liquid motion of a healthy colour has come away. Pulse at 92, with rather less power than natural. Tongue clean. Expectorates a good deal of mucus, which effort, as well as that of deglutition, is effected without pain. The incision is healing, but continues to suppurate freely.

Takes the beef-tea, arrow-root in milk, and medicines.

May 19th. In the course of a few days the expectoration and difficulty of swallowing were at an end, and no unfavourable symptom presented itself up to the 24th day after the operation, with the exception of a temporary and slight dysuria. On this day the ligature separated, the noose thereof being perfect and firm, and the dressings having been applied to the wound daily. This has some exuberant granulations, not occupying more than half an inch, which have been touched with argentum nitratum. A feeble pulsation in the tumour has been constant since the last date, and we are of opinion that it has latterly been more vigorous; the tumour itself is very materially diminished, but not to the degree that, at this distance of time from the obliteration of the canal of the vessel, we should reasonably expect. The external jugular vein was observed to pulsate on the day succeeding to the operation, and this phenomenon has been repeatedly noticed since, the action of the vein being synchronous with that of the heart.

31st. Arterial pulsation has become more distinct in the tumour, but is yet weak. It is supposed, from the situation of the latter (at the bifurcation of the common carotid), which is favourable to such, that a communication exists in that part between the the external and internal divisions. The wound is reduced to the size of a bean, is healthy, and the granulations protrude. The patient, being highly delighted at his recovery thus far, has been taking exercise in the ward of too violent a nature, such as dancing, &c. which has been only recently discovered, and the practice is of course forbidden, as the increased impetus of the circulation is owing to it.

June 14th. The aneurismal sac has grown larger within the last fortnight, and the pulsation remains equally, if not more powerful. Some pain has been lately felt at the incision, from the centre of which, a small and prominent collection of granulations emerges. It is dressed with dry lint and strapping, lunar caustic previously employed. The beating of the jugular vein has been often manifest.

24th. The uneasiness alluded to was merely temporary; the incision is now cicatrized. We are not able to detect any change in the condition of either carotid artery worthy of mention.

28th. Complains of cold and cough, which latter is dry and painful. Bowels open. Mr. G. ordered—

Pulv. ipec. gr. j. Extr. conii, gr. iv. M. ft. pil. ter die sumenda. Milk diet.

July 1st. Cough no better. To keep at the back of the mouth the extr. catechu, till dissolved.

5th. Has procured relief from the catechu.

18th. The cough has quite left him, and there exists only debility as its consequence. The pulsation, on the side operated on, has become more indistinct.

28th. Patient is suffering from suppuration of the right tonsil; a diffused swelling has taken place externally upon the neck, a little below the angle of the jaw. His countenance is very anxious and pallid. There is great impediment to swallowing. Poultices have been applied, and the tonsil opened with a lancet. Bowels emptied by mist. sennæ.

30th. Is much easier. A free exit of pus from the tonsil. Some dysphagia.

Sept. 20th. The above local symptoms subsided in due course, and the man has subsequently gained flesh. His spirits are good, and activity of body but little impaired. The swelling on the right side retains the same trifling pulsation, and the other on the left has not advanced, and is at present the cause of no inconvenience.

CASE 2.—*Aneurism of the Popliteal Artery—Operation.*

John Wood, æt. 58, admitted Feb. 21, 1831, under Mr. Tyrrell. He states that, about a year since, he experienced an attack of fever, during which inflammation, apparently involving the cellular tissue, and of the diffuse species, took place in the leg, occupying the foot and entire surface up to the knee. This attack left him deaf, and, upon application to an irregular practitioner, he was put under a course of mercury and salivated, to cure his deafness and the disease in the extremity. The leg got well after the mercury produced its usual effects upon the system, the inflammation having lasted four months. Six weeks ago only, he for the first time discovered any swelling in the ham, when he could assign no cause for its appearance; it has not enlarged during the last three weeks; but, in the commencement, its development was very rapid.

When examined upon his admission, this tumour was found to fill the entire popliteal space, and to be also distinct externally to the outer hamstring. A forcible pulsation was plainly felt in every part of it, synchronous with that of the radial artery, which beats about 60 in the minute. The superficial veins of the limb below were dilated and tortuous; there was a constant pain in the tumour itself, and numbness was often experienced in the leg and foot. Nothing indicated that the patient's health was impaired; his appetite was good, the bowels regular, and tongue clean. The functions of the heart and lungs were performed in a perfectly healthy manner. By compressing the femoral artery just below Poupart's ligament, the pulsation of the swelling in the ham was arrested; and this instantly returned with the removal of the pressure from above.

Feb. 25th. Mr. Tyrrell to-day tied the femoral artery, at the upper part of the middle third of the thigh, with a single silk ligature; and, so soon as the latter was tightened, the pulsation in the ham ceased. One end of the silk was cut close to the vessel, and the edges of the external wound were then brought together with the red strapping used by Mr. Tyrrell.

*Vespere, 9 o'Clock.* The temperature of the limb has been maintained by flannels, but is at present rather below the natural standard. Some numbness has existed ever since the operation. He has not slept, but there is no pain, except some smarting in the wound, nor feverishness.

26th. Has been rather restless, and not slept much. Is free from pain and numbness. Pulse 70—bowels not open—limb of natural warmth, even to the toes.

27th. Is devoid of fever, though constantly moving about in his bed. Face rather red—no failure of temperature in the leg, nor return of pulsation in the tumour, which has manifestly decreased. A copious evacuation has been procured by an enema.

28th. No bad symptom has shewn itself. The strapping was to-day removed, and the union of the incision is very complete.

March 4th. Wound nearly healed, and tumour in ham has certainly diminished since last report.

8th. The incision has perfectly cicatrized, excepting at the point whence the ligature emerges. He has no febrile action about him, and the ham is the seat of no pain, nor of pulsation. Bowels are regulated by enemata and the house medicine.

22d. The ligature has not yet separated, although it is kept continually on the stretch by means of a piece of cord, attached to its extremity, and fixed amongst the dressings. In every respect he has been doing well.

31st. Patient is still confined to bed, in consequence of the ligature being retained.

April 25th. Ligature has remained in the thigh until to day, when it separated with the exertion of some little force after the protracted period of two months.

May 10th. Presented well.

### Large Malignant Fungoid Tumour on the Neck. Incomplete Extirpation—Death.

John Weir, not quite 23 years of age, first mate of a vessel trading to India, and a healthy-looking and vigorous young man, applied at St. Thomas's Hospital with a large tumour situated on the left side of the neck, June 21, 1831, and was admitted on the 23d, under Mr. Green's care. When he was visited by Mr. G. and Mr. Travers also on the 24th, we collected the following statement from the surgeon who had previously had the charge of him, from his father, and himself, who, being in a station of life superior to patients generally seen in a hospital, was able to give a clear account of the origin of his disease, and the symptoms arising from it.

Shortly before his last voyage to Bombay, and about twelve months from the present period, he caught cold from exposure to night easterly wind after violent exertion in dancing; an inflammatory swelling of one or more of the cervical glands near the angle of the jaw was the consequence, accompanied by a smart degree of febrile excitement; these were, however, so far reduced by the successive application of leeches, and by purgative and saline medicines, that in the space of a fortnight no enlargement was remaining, except the induration and thickening common to such cases. He now went to sea, and before many weeks had elapsed, a distinct and firm tumour, apparently to himself and the surgeon of the ship nothing more than chronically enlarged glandular substance, attracted his notice in the same situation as the former swelling. But few remedies were tried to discuss it, the surgeon waiting till they should touch at the Mauritius, where he contemplated procuring iodine and ointment of hydriodate of potass. The latter were obtained here, and they having been used for the rest of the passage onwards some little diminution was effected, the part yet being larger than a hen's egg. Having landed at Bombay, he consulted a resident practitioner, who introduced a common lancet, but no other fluid escaped than blood, and this in small quantity. The surgeon was frightened as well as his patient, from the description of the latter; blood was afterwards abstracted locally by cupping, but nothing further to merit mention was done for him when in India. Nor during the voyage homeward were any means used to arrest the progressive increase of the enlargement which was taking place; for, owing to some internal disagreement between the surgeon of the ship and rest of the company, he declined his assistance. Upon his return to England, about the 7th of the present month, he again placed himself under his former medical attendant, residing near the Commercial Road in London, and the tumour is represented then to have been nearly equal to its present size. Great anxiety was now at length aroused within him, but notwithstanding the immense bulk no pain had been complained of, and his general health ailed not. Whilst outward bound, his mode of living was unrestrained, but coming home he abstained from wine and spirits. He is quite unconscious of having ever received a blow upon the neck, to which the commencement of morbid action might be attributed.

Being on this occasion examined by Mr. Travers and Mr. Green, the tumour seemed to the latter gentleman to have grown even within the last few days since he had seen the patient, and in this opinion he was supported by others who also inspected it at the first interview with himself. It is of irregular shape, but its dimensions may be judged of by its extending from a little anterior to the angle of the lower jaw to the median line of the back of the neck. In the centre it is conical, and at the apex, which is somewhat less than the extremity of the index finger, a slightly reddened and thin state of the integuments exists, where ulceration may be soon anticipated; at this point also the projection outwards from the plane of the neck measures between three and four inches. We trace the enlargement above to the lobules of the ear, which is thereby displaced from the skull, and below to within two inches of the upper margin of the clavicle; in the former situation it is narrow, but broad in the latter and more defined. In front again it becomes narrow, and posteriorly very broad, with its edge gradually bevelled off as it approaches the ligamentum nuchæ. To the fingers of Mr. Travers, an uniform fluctuation, as if from contained fluid, was conveyed; and the sensation was general in all parts handled. It appeared perfectly immoveable; of this particular we could not fully satisfy ourselves, as Mr. Green expressed a strong desire that the part should not be touched unnecessarily. Pain has recently been considerable; within the few days last past, indeed, he has suffered severely; it is of a lancinating character, recurring at frequent intervals, and more acute on some occasions than others. And the growth of the mass has been latterly rapid—in the course of three weeks its magnitude has been very greatly augmented. Numbedness has been experienced on the side of the head, proceeding from the ear to the vertex. The surface is rather above the natural temperature, and often feels to the patient stinging and heated. No enlarged or varicose superficial veins are observable upon it, nor any discolouration, save at the spot above referred to. There is not the least aspect of malignant constitutional affection in the man's countenance, and he affirms that no internal ailment has within his memory assailed him. No traces of such meet the eye, but yet the system is not altogether free from sympathetic disturbance. His pulse is excited, and at 96; but this frequency may arise partly from mental influence, as he has been surrounded by a large concourse of pupils, and much interrogated. The bowels are rather irregular, and the tongue white and indented at the sides; it is tumid, though moist; his appetite is excellent; has no thirst. He is perfectly void of cough, dyspnœa, or any other disturbed function of chest, or abdomen. From the action of cathartics, taken since his coming on shore, a state approaching to syncope has been generally induced with each dose, a fact shewing the facility with which the balance of the circulation is disturbed. He has pursued a sea-faring life from boyhood, and exhibits all the fortitude and high spirits of the sailor. The father is a robust healthy man, and of the same avocation; and the other members of his family are of sound constitution. His nightly rest has been broken, often entirely destroyed, wherefore opium in abundance has been taken, but not with its ordinary effect.

Mr. Travers appeared anxious to know more of the nature of the swelling by puncturing it, and such also was the wish of Mr. Green. Still they both agreed that if it were malignant, a chance of relief would be afforded by an operation, and as Mr. G. was averse to having the skin injured for any length of interval, during which a fungus might protrude from the wound, the puncture was postponed till immediately before the removal of the disease, should it turn out to be what they expected. This decision met with the entire concurrence of the patient, who has firmly made up his mind to undergo any operation that may offer a probable means of relief. An early day was therefore appointed for the pupose of setting the question at rest, viz. the 27th, and Mr. Tyrrell was requested to see the case in the mean time. Mr. Green directed 20 leeches to be applied to the part most painful—just below the central projection—and a cold spirit lotion constantly. The bowels to be opened by house medicine; and liquor opii sedativ. ♏. viij. horâ somni.

June 25th. Relief for a few hours was procured by the leeches, which abstracted a plentiful quantity of blood, but towards the evening the pains recurred, and have precluded ease as well as sleep during the night. A dose of mist. sennæ comp. was administered yesterday, and followed by two copious stools in the evening, after the evacuation of which he almost fainted. He seems now inclined for rest, and has imprudently eaten two mutton-chops for his dinner, which he himself provided. Pulse and tongue as yesterday. The heat upon the surface of the tumour is diminished, and comfort results from the application of the evaporating lotion. The anodyne draught was not given last night, as he thought he could dispense with it and it was prescribed conditionally.

26th. Has been easier, and got four hours sleep last night. His countenance is lighter and more cheerful, it having been rather dull yesterday. Another supply of leeches has just been put on. Skin is quite cool—no change in tongue—pulse 100, softer, and less irritable. One stool has been passed to day, and he is about to repeat the dose of purgative medicine.

27th. He has not been uncomfortable but is a good deal excited at the prospect of the impending operation. Pulse is nearly at 120, and weaker. Bowels have been twice moved from the physic. No alteration is externally manifest in the tumour, which has been kept wet with lotion. There has been no difficulty of swallowing, nor symptom of irritation about the larynx.

The whisker and hair from the lower part of the temple being shaved off, Mr. Green at one o'clock to day commenced *the operation*, which we believe was the most formidable we have ever witnessed.

He first plunged a lancet into the most projecting portion to the depth of an inch, to ascertain the contents ; from the puncture there issued florid blood in a free stream, which was not easily checked, and the patient became faint from the loss of the minute quantity of this fluid thus afforded. The flow was, however, arrested by plugging the aperture with lint, and applying pressure continually during the different steps of the performance. It being thus decided that pus was not a component part of the tumour, Mr. G. forthwith proceeded to extirpate the disease, beginning by making an incision from the front of the tumour to its most posterior extent, slightly curved towards its anterior extremity (with the concaving looking upwards) in order to inclose the apex, below which it passed. The whole length of the incision was somewhat elliptical, and did not penetrate beyond the integuments. A smaller one, and correspondent in shape, was then carried above the conical portion, meeting the former at some little distance from either end. Within the ellipsis of these two a considerable proportion of sound skin was comprised, together with the summit of the mass. When the superficial fascia of the neck, posteriorly, was touched with the knife where it becomes thin, it was readily divided, and in one or two parts the capsule of the tumour, which proved to be very delicate, was cut into, and from them a fluid freely flowed, of a dark green colour, mixed with blood, and of a peculiar and disagreeable odour. This was prevented from further escaping by pressure on the openings with the fingers of an assistant. The more anterior fascial covering was now detached with the knife supported on the finger of the operator, which was placed between it and the capsule ; and this latter was now fully exposed in its centre. At the present stage of the operation some idea was formed of the deep attachments of the diseased parts, the difficulty of separating which was just beginning. The sac was very firmly connected above and behind, close to the occiput, and in the division of its connexions in this situation two large superficial arteries were cut through, and in consequence of their bleeding profusely tied with ligatures. Below, and on the posterior direction, it was less strongly adherent, but excessive pain was produced by the manipulation necessary to disengage it with the knife and fingers. This point was the lowest of any we have mentioned in our account of the external appearances, and beyond it the enlargement did not descend on the posterior aspect. But in front, a portion passed behind the clavicular portion of the sterno-cleido mastoideus muscle, which could not from this cause be traced downwards to its ultimate boundaries without a great deal of careful and protracted dissection, and probably it would have been requisite to divide the muscular substance. The poor fellow, too, had become exhausted to an alarming extent from copious loss of blood and pain, he having been under the knife upwards of half an hour, and it was thought by Mr. Green, Mr. Travers, Mr. Tyrrell, and Mr. Key, who was also present, perilous to proceed with the removal of this part in the patient's very depressed state. He had vomited on the table, and by this effort the heart's action was in a trifling degree restored; but a death-like pallor was spread over his whole face, and he was for five or six minutes bordering on syncope. On this account the operation was necessarily delayed for awhile, and after it was resumed he moaned much and loudly, his pulse continuing excessively low and irregular. Brandy, pure and diluted, was given, but it did not speedily revive him. We should have stated, however, that previously to the vomiting and exhaustion supervening the sac was emptied of its contents, and cautiously dissected away superiorly. By the evacuation its bulk was diminished, its different processes rendered more distinct, and consequently its separation facilitated. The loose portions of the cyst were at last quickly removed, and with the exception of the prolongation concealed by the sterno-mastoid muscle, the entire mass also. Before the patient was conveyed from the operating theatre, several pieces of German tinder were gently inserted to the bottom of the wound, from which there was still an abundant general hæmorrhage, and there compressed. By this application, and a dossil of lint, confined by a single strip of plaister, the bleeding was restrained, and the man brought into his ward lying on the same table, and unfortunately in a very low condition. He reclined in bed, chilly, and extremely pale; seemed drowsy, and spoke not, but for water to allay his thirst. The pulse was feeble to the lowest degree, irregular, and the artery felt empty; in fine, he was barely alive, his cheeks displayed a cadaverous hue, and immediate death was looked for.

It ought not to be omitted, that from the portion left behind, covered by the mastoid muscle, Mr. Green squeezed out the soft matter filling the sac, previously to any dressing being used.

*Structure of the Tumour*. The contents of the sac exhibited an undoubted specimen of the malignant fungoid disease, in the stage of encephaloid tumour. The substance of this last was soft and readily broken down, of a dull white colour, pulpy consistence, and presenting a convoluted aspect, very similar to brain externally. In addition to this structure, there are to be mentioned the fluids which came away during the operation, very characteristic. The cyst itself was denser in some parts than in others, shining on its superficial surface, and seemingly aponeurotic in texture.

No kind of re-action having taken place, in the space of a few minutes half an ounce of brandy, with fifteen minims of tincture opii, was given to him in camphor mixture. From this a state of tranquillity was derived; but no restoration of temperature was observed by 5 o'clock, p. m., when, accordingly, the draught was repeated, though not exactly with the same quantity of laudanum.

At nine o'clock in the evening we found he had rallied very inconsiderably, his pulse being nearly as low as ever, and the feet still cold. He had complained of thirst, and some pain in the neck; had slept for a little while, and been during the whole time almost motionless. Was now only just able to speak in a whispering tone. No bleeding had occurred, nor oozing from the dressings. Hot bricks had been constantly applied to the feet, and flannels to the whole surface of the body.

June 28th. About 2 o'clock, a. m., the above tranquil state, during which the poor fellow might well nigh be regarded as lifeless, and which was probably the effect of opium in some measure, went off, and he was becoming restless. A small dose of laudanum was therefore administered, and as his system was not roused by the cordials he had taken, some brandy was united with it. Of the latter he had partaken at intervals, in sago and gruel. Shortly after four o'clock vomiting came on, preceded by pain of epigastrium, retching, and nausea. The former continued unabated up to eight this morning, the arrow-root, sago, &c. being rejected as soon as swallowed, when Mr. Whitfield prescribed a draught composed of tinct. opii, ♏. v., tinct. cinam. ʒss. aq. cassiæ, ℥j. Since taking this, he has thrown up nothing; the retching and nausea have returned, but a little tea beat up with egg has staid on the stomach. Hiccough has lately appeared, but is neither violent nor frequent. His pulse is excessively feeble, tremulous, and imparts the sensation of the vessel being evacuated. The surface is tolerably warm, and moist, but he still feels a coldness of the lower extremities. Does not acknowledge pain of head, except from the straining exertions. Countenance is less pallid, and there is a more animated expression in the eyes. His urine has been voided voluntarily, with facility, and in proper quantity; has had no motion from the bowels. About three hours have been spent in sleep since nine o'clock last evening. The wound has been easy, but not interfered with.

Mr. Green ordered at his visit, at two o'clock, one grain of opium immediately. Brandy to be continued in cold arrow-root; *a plaister* of mustard to the scrobiculus cordis; and the bricks, &c. externally.

29th. No sleep has been enjoyed, but he has not been suffering pain. He is nearly as low as yesterday, though having more power to speak. His pulse is up to 140, and of the same weak character—whole surface of the trunk and extremities naturally warm, and the countenance betrays less of the deathly pallor. Hiccough has been present throughout the night, and has not yet ceased, but there has been no recurrence of vomiting; the former comes on about every half hour, and lasts for the same period before it goes off again; it is feeble, short, and not loud. He makes water easily; bowels not moved. Some swelling is observable around the incision, but no discolouration. About a pint of brandy has been consumed since the operation, and six ounces since yesterday. He has also taken jelly, blanc-mange, and arrow-root, in good quantity. At five, p. m., Mr. Green saw him, when the hiccough was unrestrained by opium, capsicum, and effervescent mixture, which had been severally tried by the dresser during the day; and no evacuation was procured from the bowels. An injection of gruel and common salt was directed to be thrown up directly for the latter purpose.

9, *p. m.* The hiccough has alternately ceased and returned for half an hour, and it is now very feeble and short, but most distressing to the patient, who is labouring principally under this symptom, and moans in a most pitiable manner after each spasmodic action of the diaphragm. An enema containing ʒij. of laudanum and two ounces of starch has been injected, and the hiccough was checked for a short time by it, as it was previously by the cayenne also. The pulse is 140, very weak. It, as well as the system generally, is quite under the influence of opium, which has been liberally supplied through the day.

30th. He has been comfortable, with the exception of the occurrence of hiccough, which has however been less frequent, and indeed absent for an hour together. At six this morning he took opii, gr. ij. and the pulse is now equally accelerated as yesterday, and the skin hot, and rather dry. There is a marked improvement in the countenance. Several purgative glysters have been employed without any effect; consequently, no stool has passed since the day of operation. A second opiate injection was likewise exhibited last night, to allay the hiccough, and with some temporary advantage. Says he has appetite for chicken, and some is in preparation for his dinner; has taken a fair proportion of nutriment in the form before mentioned, together with brandy and effervescing lemonade. A bread poultice is laid over the dressings on the neck, in consequence of uneasiness in the wound.

9, *p.m.* One scanty stool was discharged an hour ago, without glyster, or voluntary exertion. No opium has been had recourse to during the day. His pulse has varied from time to time, both in fulness and frequency—it is now very rapid and soft; skin temperate and moist; breathing free; hiccough recurs less often; neck is still uneasy. Ate two or three slices of chicken at two o'clock.

July 1st. Severe pain has been experienced in the neck the greater part of the night, and an offensive smell, as from sloughing, arises from the wound and dressings, along with some purulent matter. There is rather more surrounding swelling, but no redness. His night has been sleepless on account of the pain, and the hiccough has been unimportant, and appears gradually going off. Three stools have been passed, not copious. Pulse is 130, feeble but fuller, and has been less changeable—skin warm, and has some moisture—tongue furred, white, and marked by pressure against the teeth—thirst diminished. The whole of the German tinder and lint were removed from the wound, which, owing to the complete separation of its edges, was of course very extensive, and its surface was sloughy. There were, apparently, deeper sloughs towards the anterior part. Some pus escaped, the fœtor of which and the entire part was most intense. At the circumference, posteriorly, two granulating spots are visible, pale and smooth, and, to our eyes, disposed to assume the fungoid character. Lint, steeped in solution of chloride of lime, was placed upon the foul parts, one or two adhesive strips to support the sides of the wound, and a bread poultice afterwards. Mr. G. did not think a continuance of brandy (whereof ℥jss. has been consumed during the last twenty-four hours) necessary, but ordered him to drink port wine and water, in such quantity as circumstances might demand; the jellies, arrow-root, &c. as before, with whatever meat he might fancy for his dinner; and the following draught every six hours—

℞. Mist. camphoræ, ℥jss. Ammon. subcarb. gr. v. Liq. opii sedativ. ♏viij. M.

9, *p.m.* The neck has been easier, but a slight erysipelatous redness is now developed on the cheeks. Some chilliness, bordering on rigor, has been observed by the sister, but the patient denies having had the sensation. Pulse the same. Has taken the wine and water and a custard pudding; could not take so much meat as yesterday. Slept a good deal in the day.

July 2d. There is increase of erysipelas upon the face, but not important. During the night, the redness was very bright at one period, but it is at present of a light hue, and not accompanied with much effusion or tension. The disease has not made much progress below the wound or behind it, being here limited to a very few inches. Pulse very rapid and feeble, at 134—skin hot—great thirst—tongue white, more furred and loaded. A colocynth enema was injected last night, and, although some time retained, no alvine matters were solicited, and this morning no motion has come away.

Mr. Green was present about 3, *p.m.* and ordered—

Hyd. submur. gr. v. Ammon. subcarb. gr. x. M: statim sumend.

The former draughts, wine, jellies, &c. to be continued without any alteration.

9, *p m.* Erysipelas has spread in every direction, but the inflammation is not of an active description, the redness being not at all vivid, and there is no confusion of ideas nor headache—the swelling is greater—has dozed much—the bowels have been once sparingly moved.

3d. The erysipelatous inflammation has extended in each direction, more particularly downwards toward the chest. Still he has not complained more, and the pulse retains the same characters, being 130 in the minute—tongue not changed—has had some sleep—bowels once open. Wound does not shew larger sloughs, but the discharge is very offensive, and the granulations appear as though inclined to fungate. Mr. Green did not see any indication for change in the treatment, and ordered brandy to be resumed if the strength should fail.

4th. Further diffusion of erysipelas is now noticeable, with increased tumefaction of the face. His pulse is at 125, but more feeble, and the countenance is, moreover, more fallen—the tongue, however, is cleaner and moist—skin temperate—bowels free.

Remedies as before. Cold saturnine lotion to the inflamed parts.

5th. The inflammation occupies a considerable portion of the opposite side of the face, and the swelling has augmented. There is also more of the redness upon the trunk. Pulse was on last evening irregular, extremely depressed, and of the previous frequency. Skin is temperate—two motions from the bowels—wound much in the same state, but yields less discharge. Wine, &c. taken in good abundance; and he does not suffer much pain, either locally from the wound and erysipelas, or in his head from the latter cause.

6th. Was delirious during the night, and talking incoherently for some hours. The erysipelatous redness has in some degree declined, and the swelling also, and none of the face is further involved. More of the trunk is, nevertheless, implicated anteriorly. He is exceedingly low, but does not refuse nourishment. Pulse 130, regular, feeble—tongue cleaning—bowels acted once in the night—discharge from wound still scanty. Ten ounces of port wine have been given to him in water during the last 24 hours, with the other means; and Mr. Green now prescribed—

Quinæ sulph. gr. ij. Pulv. capsivi, gr. iij. M.

to be taken either in combination with the former draught, or in any other fluid menstruum the patient might prefer. To encourage suppuration from the neck—

Catapl. Cerevisiæ to the wound three or four times daily.

7th. We were unable to visit him in the morning, but his condition was not, to appearance, then altered. Delirium had been pretty constant throughout the night, and he had been in much agony, principally from the neck, though quiet. At 7, *p.m.* his breathing was laborious and sonorous; the left eye had become dim, and was copiously besmeared with a thick and adhesive mucus; the right was closed from erysipelatous swelling of the lids. These latter were no longer red, and the whole countenance had a dingy and death-like cast. The respiration had been thus affected about an hour; five or six griping and liquid motions had involuntarily passed from the bowels, and severe tormina were continuing every now and then; the contents of the bladder likewise escaped unconsciously. There was coldness of the extremities—in fact, he appeared to be sinking, and not slowly. Yet the pulse was not more depressed, nor irregular, nor numerically increased. He had not rejected any food, but eaten some meat about the middle of the day, and the medicines, wine, jelly, &c. were taken as before. Some brandy was now brought to him, diluted in warm water, and an enema of starch and laudanum thrown up the bowels. The wound is discharging freely of pus, and sloughs are detached with each poultice.

8th. After a repetition of three clysters, the two first being followed by loose and painful stools, the diarrhœa was checked and the abdominal pain subsided. He shortly became tranquil and slept, awaking at intervals, during which his wandering state was very manifest. His respiration is to-day nearly natural, greatly easier, and he has rallied in a manner quite astonishing. He is not in pain, but his sensations are so very depressed, that he cannot at all adequately explain them. Has occasional tormina, but there is no tenderness of belly. The erysipelas upon the abdomen seems to be of the erratic species; the redness is here very faint, and the affection, to the eye, involves nothing more than the epidermis. Pulse has been variable; it is now ample, at 130, and readily compressed. Skin of every part warm—tongue is tolerably clean, and whitish. Wound of the same appearance, and the beer-grounds poultice is continued to it.

Medicines, &c. with brandy, have been supplied as ordered. To repeat the starch injections, with xxx. drops of laudanum in each, if diarrhœa should persist.

9th. Two enemata have been given, on account of the diarrhœa, and each time relief was obtained. The patient is in just the same low state as yesterday, not having power to retain his urine, but has been rather easier. His mind has been often wandering, in the midst of a dozing and heavy condition. Respiration is becoming embarrassed—pulse full and regular, not at all stronger—deglutition quite perfect—Erysipelas is much diminished upon the trunk, and is scarcely worthy of the title.

10th. He continued no worse throughout yesterday, but, at two o'clock this morning, breathing grew more difficult, and his powers lower than ever. Increased purging also supervened, which was, for a time, alleviated by the starch and opium administered by the rectum. Up to the hour of his death (12 o'clock, noon), he was sensible, though occasional incoherency had existed in the night, as previously, and did not lose the ability of swallowing the stimuli which were constantly poured in. The erysipelas had disappeared from the body, and scarcely a vestige of it could be found about the head; but his sufferings were great, from pain in the wound.

*Dissection*, 26 *Hours after Death.* Superficially, the wound was sloughy, but the extent of it, compared with the space occupied by the tumour during life, was very much contracted. In the centre of the foul mass, there were three or four small fungoid growths, which, when cut into, displayed an internal spongy structure, of a dark purple colour, and from which blood of the same hue was even now expressible. We might say that the disease was included in the superior and posterior anatomical triangular division of the neck. At its lower part, it did not quite reach the upper border of the omohyoideus muscle; it also became more superficial inferiorly, nothing save the thin cyst here existing, upon which some of the external and descending branches of the cervical plexus were spread. Here, too, were found two bodies, presenting the appearance of common enlarged glands, though having a white investment, little larger than almonds, in which condition we are led to infer the malignant tumour originated. By detaching both the origins of the sterno-mastoid muscle, and turning it upwards, the limits of the diseased structure anteriorly were seen. It passed behind this muscle, opposite to the corner of the os hyoides, so far forwards as its anterior edge, and it was in this situation separated from the common carotid artery, where it bifurcates, merely by its condensed sheath. Its margin was tolerably defined, and the portion was easily disengaged from its cellular connexions. Behind, the morbid structure did not proceed beyond the anterior border of the trapezius. Great difficulty arose in making out the parts superiorly, from the loss of substance consequent upon the sloughing. Portions of dead cyst were discovered above and behind the angle of the jaw, quite contiguous to the parotid gland, which must have been thus forcibly pressed down, if not implicated with the sac itself. The posterior part of the mastoid process, and the occipital ridge beyond that projection, were for some distance exposed, so that the fingers could be placed directly in contact with their denuded surfaces for more than one inch, amidst the disorganized textures. The deeper muscles of the neck were slightly involved, more particularly the complexus and splenius capitis; the transverse process of the atlas was deprived partially of its coverings, and the recti postici and obliqui capitis were to be beheld on clearing away those parts deprived of vitality; but, with this exception, the disease had not penetrated deeper than the plane formed by the internal surface of the trapezius and mastoid muscle. It was covered by the latter in front, as we have before noticed, but, posteriorly, the trapezius did not overlap it. The omo-hyoideus was not in the least displaced; but, from the pressure exerted by the process in front, the internal jugular vein, carotid artery, and par vagum, had become matted together by adhesive depositions within their sheath. Beneath the cellular tissue of the neck, on the right side of the wound there was a plentiful infiltration of pus, the effect of the erysipelatous inflammation. Pus was likewise met with in the cellular texture between the pharynx and spine. Both lobes of the thyroid gland were enlarged and indurated, and no longer possessed of their flattened shape. When incised, the surfaces appeared semi-transparent and resinous. A remaining portion of the thymus gland, about the size of a horsebean, was discovered in the anterior mediastinum, also endowed with morbid qualities, in appearance the same as those which constituted the disease in the neck. Upon section of this body, a grumous substance was presented externally, whilst the interior was whitish and pulpy. In the central lobe of the right lung, extensive disorganization had taken place, in the form of white and medullary deposits, of the consistence of soft cheese, and about equal to walnuts. An unusual collection of adipose matter was visible on the external surface of the pericardium. Within the larynx, there was a reddened state of the epiglottis, and of the lining membrane of the arytenoid cartilages.

The stomach and large intestines were greatly distended with flatus, but healthy in texture. The liver was externally of a peculiarly dark livid tint, except at its anterior thin edge, which was natural. It was partially emphysematous underneath its peritoneal coat, produced by rapid decomposition; in the middle it was very soft, and of a dirty opaque cast, the natural granular structure being lost. The cortical substance of the right kidney was softened, and, within it, the same medullary growths had sprung up as in the lung. Some old adhesions were detected between different parts of the intestines and the peritoneum. Prostate gland somewhat enlarged. Pancreas, spleen, and mesentery were healthy.

Owing to the intense heat of the weather, putrefaction had been rapidly advancing, espeially in the neck and face. The odour exhaled from the body was extremely offensive, and this was augmented tenfold by the sloughy condition of the wound.

Beta.

---

### V.

### Dr. Ogden (of Sunderland) on Cholera.

The following highly valuable letter from Dr. Ogden, of Sunderland, to the editor of the Medical Gazette, we here republish, as it cannot fail to check a little the ultra-contagion mania which has too long prevailed, to the ruin of thousands and the loss of many lives.

"The first cases which were publicly made known as epidemic cholera were those of the Sproats, three in number, one of which proved fatal on the 26th, another on the 31st of October. But several sporadic cases, exactly similar, had occurred from the commencement of August.

1. The first case that I am aware of was that of a middle-aged man, a potter, living near the river on the north side, about three miles from the harbour mouth, and two from the town of Sunderland. The case fell under the care of Mr. Dixon, who, at the time, considered that it differed in nothing from the disease described by the Indian writers, and who has not since seen reason to alter his opinion. The man recovered slowly.

2. The second was that of — Arnott, a middle-aged labourer, living at Pallion, on the south bank of the river, about a quarter of a mile higher up than the first case. His residence was 50 or 60 feet above the level of the river, and about the distance of a stone's throw from it. He became ill on the 8th of August, and died in twelve hours. He also was under the charge of Mr. Dixon, with whom and Dr. Brown I had an opportunity of seeing the body the next day. I certainly have not since seen one in which the external appearances after death were more characteristic of malignant cholera. The collapsed face, sunk eyes, surrounded by a dark circle, blue hands and nails, contracted and shrivelled fingers, and great rigidity of the muscles, were most strongly exhibited. No examination was allowed.

3. The case of Robert Henry, æt. 43, a pilot, occurred next, and terminated fatally on the 14th of August. He lived in a row of cottages near the mouth of the harbour, at the east end of Sunderland, and in an airy situation.

4. — Pearson, a shipwright, middle-aged, living at the western extremity of Bishopwearmouth, was the next. He became ill on the 27th, and died on the 28th of August. He was a patient of the Dispensary, under the care of Dr. Haslewood, who assures me that every symptom of Indian cholera was present.

No connexion between any of the above cases could be traced, nor had they (with the exception of course of the pilot) any connexion with the shipping. They were regarded as cases of aggravated English cholera by the majority of the medical men, though it was strongly suspected by some that they were the forerunners of the epidemic.

Cholera continued very prevalent and severe during September, but I know of no deaths from it in that month. I have been informed by very good authority that a fatal case occurred in a middle-aged man, about the 12th of October; he had not been seen by a medical man, but he had spasms, and the characteristic vomiting and purging and died rapidly.

Mr. Holmes has obligingly furnished me with the following information concerning the Sproats, who were originally under his care :—

1. — Sproat, æt. 60, labourer, Long Bank, Sunderland; died 26th October.

2. — Sproat, æt 10, his grand-daughter, became ill half an hour after his death.

3. — Sproat, æt. 32, son of No. 1. father of No. 2, became ill on the 27th, Nos. 2 and 3 were removed to the Infirmary; No. 2 recovered; No. 3 died on the 31st. On the same day died,

4. — Wilson, a keelman, High-street Sunderland, and,

5. — Rodenby, a shoemaker, Monkwearmouth Shore; and, on the 1st of November died,

6. Elizabeth Turnbull, nurse at the Infirmary, who assisted in laying out the body of No. 3.

Between Nos. 1, 2, 3, and 6, there was an evident chain of connexion; 4 and 5 were distinct, and isolated from all known sources of contagion.

No deaths occurred between the 1st and the 6th of November; on the latter day there were several, on the 7th more, and on the 8th the cases were numerous, and the existence of the epidemic amongst us could no longer be doubted.

As to the origin of the disease, a subject involved in the utmost mystery, I am inclined to believe it to be as indigenous here as it was in Jessore in 1817. In hazarding this opinion, I am aware that I am in opposition to a mass of very powerful evidence, collected by the advocates of the doctrine of contagion; but whilst I firmly believe the disease to be contagious, I think also that too much has been ascribed to this principle. Contagion once granted, its supporters have attributed to it every case of cholera that occurs. Now if the disease arose spontaneously in one individual, it might in a hundred, or a million.

There has been great intercourse between this port and Riga, Petersburgh, and Archangel, during the existence of cholera at those places; and it was the mortality among the British sailors, of whom several were our townsmen, that occasioned the institution here, of, I believe, the first Board of Health in the kingdom. It has been supposed that the sailors, on their return from those ports, brought the disease with them; the quarantine which is a human institution, and incapable of subverting the laws of nature, being too short to extinguish the 'germs,' or the 'seeds,' figurative terms by which diseases are said to be propagated. But if the sailors brought it with them, it was much more likely that it should make its first appearance in some other port than this; for although many Sunderland vessels go every year to Petersburgh, &c. the intercourse is indirect, and an exceedingly small proportion return direct to this port. They return with cargoes to almost every port in the kingdom, from Aberdeen round by the south and west to Glasgow. Independent of the vessels which trade directly between this country from London, Liverpool, Bristol, Hull, and other places, there have been more Sunderland ships from Russia delivered at any one of the ports just named than at Sunderland.

It is to be recollected, however, that when the foreign voyages were over, and the ships returned to the coasting trade, there was a concentration, a *reunion*, in Sunderland, of a great number of persons who had been, at some period of the Summer, in the unhealthy towns. Every sailor too has a chest in which he keeps his clothes, and whose entire contents he would bring to his family in the town, for purification. Moreover, some of the chests which came home had belonged to persons who had died abroad, and had probably never been opened since the clothes of the deceased were put into them. The combination of these circumstances may perhaps be thought sufficient to account for the introduction of any contagious disease. But they do not explain the unusual prevalence of cholera during the Summer, nor the fatal cases of August; nor has it been observed that the families of sailors have been more visited by cholera than others. Sailors themselves have been wonderfully exempt during its prevalence here. Besides, the sailors of London and other places died abroad, and their clothes would doubtless be sent home in like manner; yet they have not brought cholera.

Whatever, therefore, were the facilities for the importation of cholera here, they were much greater in other places. So far from following the 'great routes of human intercourse,' it has chosen one of the least frequented paths.

Here I beg leave to correct an error which appears at page 238 of this volume, where it is stated that a vessel from Hamburgh performed quarantine in the Wear. No ship has entered the harbour from Hamburgh without a clean bill of health, which expresses not only that the crew, specifying their number, are in 'perfect health,' but that, up to 'that period, perfect health prevailed in the town and port of Hamburgh, and the country adjacent, which continued free from cholera morbus, or other contagious or infectious disease.' This bill of health is granted by the British Consul at the place. In addition to this, every vessel from Hamburgh, since June, has been examined by a medical man before being liberated. What have been styled suspected vessels, were ships from Holland detained under observance or precautions; and it is to be recollected that, up to this day, there is no suspicion of cholera in Holland.

That the spreading of the disease is the effect of some unknown power, very different from contagion, is, I think, evident from the phenomena which it has presented in this country. Newcastle, a large and populous town, within twelve miles of Sunderland, and holding daily intercourse with it, escaped the disease a whole month. Gateshead, another populous town, on the *hither* side of the former, and holding similar intercourse with both it and Sunderland, escaped for another month. London, holding constant intercourse with all these places by land and by sea (by sea it is said to travel most rapidly) yet remains free, nearly three months after the appearance of the epidemic on our shores. The contagionist says this only proves the inhabitants of these places not to be predisposed to the disease at the time the first emanations of contagion reached them. The reasoning is very convenient; it assumes that contagion is constantly travelling, and arriving at these places, but that it is not until the constitution is undermined by predisposition that the population falls a prey to the venom of contagion. A corollary of the same proposition is, that *predisposition travels*; for contagion is assumed to be constantly travelling, but it is only when accompanied by predisposition that it is rendered obvious, by its effects, to the senses. Another contagionist reverses the order of the causes, and alleges that contagion constantly spreading, predisposes all the districts surrounding an infected place; the disease then lies dormant, or latent, in the constitution, and all that is required to develop it is certain atmospheric vicissitudes, or debilitating causes, the peculiar nature of which it is not easy to comprehend, or is, perhaps, beyond our comprehension. But the former mode of reasoning is the most usual, namely, that contagion travels, and predisposition renders liable. If a populous town on one side of a river suffers from cholera, and another on the opposite side is free from it, it is because the people on the one side are predisposed, whilst those on the other are not. If the disease arises in Sunderland, and is presently found in various districts to a hundred miles north, and not to the distance of ten miles south, it is because the inhabitants to the north are more predisposed than those to the south. In short, it is a postulate with the contagionist, that, for the disease to prevail, the people must be predisposed; but what this predisposition consists in we are never told. We have certain general ideas about the predisposing effects of intemperance, exhaustion, privation, and the like; but these are not sufficient, for we find the people of one place just the same in these respects as those of another, but we never discover which of them were predisposed until the disease has attacked them. Hence, to say that people *must have been* predisposed, because we see that they have been attacked, conveys no information whatever. The term predisposition, in this loose acceptation, appears to me inadmissible in scientific language; it merely expresses an unknown antecedent state.

Even in a narrower circle, the effects of contagion are apt to be overrated. Nothing is more common than to find, where a death has occurred, that the rest of the family are affected with diarrhœa, which, if neglected, runs into fatal cholera in some of them. Hence a succession of deaths, giving the appearance of one person having taken the disease from another, whereas they were all affected with the precursory diarrhœa at the same time. In a family too there is generally a similarity of constitution, habits, clothing, diet, residence, and other circumstances; and it is peculiarly likely that the cause, whatever that may be, which produces cholera in one member, shall have the same effect on the others. Indeed, it is remarkable that it has so frequently attacked one member of a family, and spared the rest, who might be supposed more than ever liable to its visitations, from the fatigues of night watching, and the depressing effects of grief; and what was unfortunately too frequently the case, the sudden deprivation of the earnings of the deceased, who had been their main support.

Throughout the whole epidemic, cases were constantly occurring wherein no connexion with any source of contagion could be traced. This was most striking in the commencement, about the 6th, 7th, and 8th, of Nov., when the disease suddenly manifested itself in many separate points totally unconnected. I can hardly conceive it possible to account for this by contagion, unless we conceive the whole atmosphere filled with it. After so many foci of contagion had been established, it was not so easy to gainsay the possibility of a person's having been exposed to it; but the number of persons obviously exposed to it with perfect impunity, was very great. Medical men have been obliged to perform the duties of nurses, to assist in the application of warmth and friction, restore the patient to a proper position when he would not remain a moment at rest; to bleed, lift to the bath, raise the head, and administer medicine. After death, they have inspected the bodies, lifted them out of bed and replaced them, and imbued their hands in the animal fluids; yet no death, and perhaps scarcely a case of serious illness, has occurred among them. And I may here state that I have not heard of any medical man conveying the contagion of cholera to his other patients.

Having said that in many families cholera has attacked one person alone, I ought also to state that in others it has committed the most dreadful ravages, three, four, or more individuals being destroyed. In some cases there is much probability of their having taken it one from another by contagion; in others there is not this probability. Thus,

1. Robert Henry, pilot, died 14th August.

2. Margaret Henry (unmarried), cousin to No. 1, became ill Dec. 11th; died Dec. 15th or 16th.

3. John Parkin, ætat. 4, nephew of No. 2, became ill Dec. 12th.—Recovered.

4. Margaret Henry, æt. 13, daughter of No. 2, became ill Dec. 13th; died same day.

5. Mrs. Parkin sister of No. 2, mother of No. 3, became ill Dec. 16th; died Dec. 18th.

6. William Henry, alias Thompson, son of No. 2, became ill Dec. 17th.—Recovered.

No. 5 was in frequent attendance on No. 2 until No. 3 required her attention. Both 3 and 5 may have got it from 2.

Nos. 4 and 6 lived in the same house as 2, and may have had it from the same common cause.

Again,—

1. James Ellemore, æt. 60, died Nov. 6th.

2. Elizabeth Hopper, an elder sister, died Nov. 17th.

3. Jane Johnson, another sister, died Nov. 28th.

4. Thomas Ellemore, æt. 67, a brother, died Dec. 25th.

5. Ann, wife of No. 4, died Dec. 27th.

The first four persons lived in separate houses, from a hundred yards to half a mile distant from each other, and were ill at very different times. What the intercourse might be, I do not know.

On the important subject of cure, I fear there is little satisfactory to be said. The number of recoveries from *cold blue* cholera has borne a very small proportion to the deaths. Of the 330 recoveries reported, a great majority were not attended with the coldness and lividity of the extremities, great collapse, and loss of pulse, so characteristic of the dangerous cases.

The Indian rule of bleeding has been freely tried with various success; sometimes blood could be obtained, at others it could not; in the latter case, no harm was done by the attempt. When blood was obtained in the commencement, it was often productive of benefit, the circulation becoming equalized, the patient expressing great relief, the pulse improving and continuing perceptible to the termination, whether in death or recovery. Sometimes, again, it appeared to diminish the powers of life. On the whole, it is a doubtful remedy; a criterion is wanting to guide us in the employment of it. Dry external heat is of great importance; hot air baths have been tried, but the deadly coldness of the extremities is totally unaffected by them; the contact of hot solids, as bricks, bottles, bags of sand, is much better. Friction of the extremities with hot flannel should be next to incessant: it is one of the few means we possess of restoring the circulation; indeed there have been recoveries principally due to the assiduity with which it was employed. Brandy is always used, often rejected a few minutes after it is swallowed. Opium sometimes relieves the sickness, sometimes it does not. Calomel, in the majority of cases, appears to possess little or no efficacy. Viewing the mucous membrane and the skin as one continuous surface, and considering the suspension of their powers of absorption, we are led to the employment of such remedies externally and internally as act as mere stimulating applications. Sinapisms are to be applied to the epigastrium, to allay the vomiting, and to the calves and thighs, to cure the spasm. A mustard emetic has often succeeded in restoring the pulse. Oil of turpentine by mouth in ℥ss. doses, and by injection, is a powerful stimulant. Camphor and the essential oils of mint and cajeput are inferior to oil of turpentine in stimulating power. I have not seen any trials of the fixed alkalies, but have heard of their failing like every thing else.

The consecutive febrile state is to be treated like fever under any other circumstances. There is usually very great cerebral oppression, indicating blisters to the nucha and shaving of the head. Small doses of calomel are also beneficial. The greatest care is required in avoiding errors in diet during convalescence.

Dissection has hitherto revealed nothing which accounts for the formidable train of symptoms whose cause is sought. There is an accumulation of blood in all parts of the venous system, with the curious exception of the vena portæ and its branches. The engorged state of the lungs, and the oppressed respiration during life, point to the respiratory system as principally suffering, either idiopathically, or symptomatically of some altered condition of the nervous system.

*Prophylaxis.*—The attack is generally preceded by diarrhœa, varying in duration from six hours to three days: this is the critical time for successful treatment. I have found pills prepared according to the following formula very successful in arresting the diarrhœa:—

℞. Opii, gr. xv.

Hydrarg. Submur. ʒj.

Pulv. Bacc. Capsici, ʒiss. vel ʒij.

Confec. Rosæ, q. s. ut fiant pil. lx.

As they are in constant request when visiting the abodes of cholera patients, it is convenient to carry a box in the pocket. One or two may be given every three hours until relief is obtained. They produce a state of constipation, which continues two or three days, and which it is quite unnecessary to remedy by laxatives, as the bowels soon resume their natural functions. When there is much pain of abdomen or griping attending the diarrhœa, it is alleviated by hot fomentations or a sinapism.

During the prevalence of cholera, there are observed among persons otherwise healthy, various anomalous affections of the nervous system, as spasms and cold sensations of the hands, feet, and legs, peculiar thrilling sensations of the extremities of the fingers and toes, and a feeling of great anxiety in the præcordial region.

I am, Sir,
Your obedient servant,
HENRY OGDEN, M.D.
*Sunderland, Jan.* 14, 1832."

### VI.

### ABSTRACT OF CASES OF INSANITY BY DR. MACROBIN, WITH POST-MORTEM APPEARANCES.

[Aberdeen Lunatic Asylum.]

*Case* 1. Andrew Braid, ætat. 56, admitted 1816, with monomania elata, and partial dementia, and died October, 1830, with symptoms of pneumonia—having exhibited no marked physical symptoms during his confinement, excepting having had a very irregular and intermitting pulse, and some symptoms referrible to the cardiac region.

*Autopsy.* Head.—No increased vascularity of the membranes; substance of the brain remarkably firm; bloody points numerous and large; an ounce and a half or two ounces of serum in the ventricles.

Chest.—Recent lymph on left pleura; great engorgement of both lungs, especially the left, which was friable, granular, and infiltrated with sero-purulent fluid. Left ventricle of heart hypertrophied and dilated. Abdominal viscera healthy.

2. John Mason, ætat. 64, admitted 1818, with mania epileptica, which appeared at puberty. Was found dead in bed, having apparently expired during a fit of epilepsy, October, 1830.

*Autopsy.* Head.—Remarkable conformation of the cranium, being very full and prominent behind, and much compressed at the sides; left hemisphere of the brain much fuller than right; great venous turgescence; but no increased vascularity.

Chest.—Lungs gorged with a sero-sanguinolent fluid. Left ventricle of the heart hypertrophied.

3. George Thompson, ætat. 28, admitted 1823, in a state of fatuity, and died July, 1830, with symptoms of phthisis pulmonalis, having greatly recovered his intellectual powers a day or two before death.

*Autopsy.* Head.—Presented nothing unusual.

Chest.—Numerous tubercles in both lungs in different states of softening.

Abdomen.—Incipient thickening and ulceration of the mucous membrane of the ileum and colon.

4. Isabella Ross, ætat. 50, admitted July, 1829, with maniacal excitement, and in the course of a month lapsed into fatuity, and died April, 1830, with general dropsy and diarrhœa ; had been long addicted to indulgence in spirituous liquors before admission.

*Autopsy.* Head.—No appreciable lesion of the brain or its membranes.

Chest.—Tubercles and vomicæ in the superior lobes of both lungs, with serous infiltration.

Abdomen.—Liver exhibited the friable granular bright yellow degeneration ; ulcers of a small oval shape throughout the ileum ; the mucous membrane of the cæcum and colon was remarkably thickened, rugous, fungoid-looking, and of a dark colour, and presented here and there large, foul, irregular ulcers : the cells of the colon were obliterated.

5. Peter Keith, ætat. 66, formerly a seaman in the navy, admitted September, 1829, with monomania elata, and imperfect paralysis of all the voluntary muscles, which increased to complete paralysis sometime before his death, which happened January, 1831. He had been addicted to spirituous liquors.

*Autopsy.* Head.—Copious gelatinous deposit over the entire surface of the brain, forming a layer of some thickness over the anterior lobes. Between the middle and posterior lobes, and near the surface there was situated an irregular cavity with vascular parietes, containing the debris of softened cineritious substance. The ramollissement extended to the medullary substance, and there was a well-marked approach to ramollissement of the whole cerebral mass, especially of the cineritious part.

6. James Simpson, ætat. 38, a seaman, admitted February, 1831, with maniacal delirium, very much resembling delirium tremens in regard to the nature of the delusions, the profuse perspirations and the constant pervigilium which were present throughout, though the tremor was absent. After a period of nearly three months he died in the last degree of emaciation, uncontrollable diarrhœa having supervened. He suffered two attacks of epilepsy, followed by strabismus some time before his death. Was reported to have been *habitually* sober, but experienced the above attack immediately after a fit of intoxication.

*Autopsy.* Head.—Well-marked increased vascularity of the pia mater, with *great* effusion into the ventricles.

Abdomen.—Inflammation and ulceration of the colon.

7. George Dawson, ætat. 38, a shop porter, of plethoric and apoplectic tendency, having suffered a slight shock four months previously. Admitted November, 1830, under great maniacal excitement, which gradually arose to the most violent delirium, under which he sunk exhausted in the course of a month.

*Autopsy.* Head.—Slight albuminous effusion under arachnoid, confined to anterior and upper part of the hemispheres : much turgescence of veins of pia mater.

8. J.G., a female, ætat. 20, admitted July, 1830, with monomania depressa, and died 22d of the same month, apparently of exhaustion, consequent on inanition from abstinence, previous to admission.

*Autopsy.* Head.—No morbid appearance discernible ; vessels of mucous membrane of ileum arborescent.

9. M.M., a female, ætat 72, admitted in 1808 with monamania elata ;—died 1831, with symptoms of œdema pulmonum, having had anasarca of the lower extremities for some weeks.

*Autopsy.* The different cavities exhibited no morbid appearances, with exception of great condensation and hepatization of the whole of the right lung, with two quarts of serous fluid in the sac of the pleura of same side.

10. I. B., ætat. 83, admitted 1812, with dementia senilis—general paralysis having gradually supervened. Died 1831, with symptoms of pneumonia.

*Autopsy.* Head.—Very copious effusion of serum over the entire surface of the brain, and into ventricles and sheath of the spinal chord, amounting to at least five or six ounces.

Chest.—Lower lobe of left lung in a state of great engorgement and hepatization; passing into purulent infiltration. Hypertrophy of left ventricle of heart.

11. Margery Grant, ætat. 82, admitted 1816, with dementia senilis. Died September 1831, having suffered from occasional severe attacks of diarrhœa.

*Autopsy.* Head.—Nothing morbid observed.

Thorax.—Upper part of the right lung tuberculated.

Abdomen.—Ulceration of ileum and colon.

12. Alexander Robertson, ætat. 41, a plumber, admitted February 1830, labouring under a state of great fatuity and general paralysis, constant tremor of the hands, and loss of speech; all of which symptoms followed upon an attack of apoplexy which occurred three years previously. Had been much subject to head-aches, and was reported to have been habitually sober. Died Nov. 1831.

*Autopsy.* Head.—Highly vascular layer of dense but recent lymph under the dura mater, covering the whole of the right hemisphere, varying from three to fonr lines in thickness, and causing adhesion of the membranes. Great vascularity of the pia mater, extending to the base of the brain and between the convolutions. Ventricles *greatly distended* with serum. Thoracic and abdominal viscera quite healthy.

13. J. W. ætat. 50, admitted 28th October, 1831, with hypochondriacal monomania, and exhibiting great emaciation, complete anorexia, much uneasiness of the epigastrium and parched, smooth, florid tongue. Died on the 16th November following.

*Autopsy.* Head.—Pia mater rather vascular, but no effusion over the surface or into the ventricles of the brain.

Thoracic and abdominal viscera healthy—the liver only containing two small tubercular masses. The whole tract of the alimentary canal was particularly examined, and exhibited no vascularity or other lesion to correspond with the symptoms of epigastric uneasiness, the state of the tongue, &c. during life.

I have given the foregoing abstract without commentary, and the only remark I would now venture is, that the nervous system still presents a wide field for pathological research, and which must be carefully explored, before we can pretend to assign to each case of insanity its true relations, and to draw any thing like general conclusions regarding the nature of those lesions which give birth to this disease in its varied aspects.

It appears to me that too much has been ascribed by recent pathologists to *increased* vascularity of the membranes, an appearance often doubtful and difficult to decide upon, and at all events often incidental to the morbid actions immediately preceding death; and that too little is allowed to the probable lesions of the nervous mass itself. We know that the intellectual functions are sometimes restored shortly before death, and that the accession and removal of the disease is not unfrequently sudden; we ought not therefore to anticipate on all occasions well-marked structural derangements. Insanity, indeed, may originate in different pathological conditions of the encephalon. And these conditions are no doubt, in some instances, associated with increased vascularity of the membrane, giving rise, I suspect, in the end, to the immense collections of serum not unfrequently observed in the brain of those who unhappily so often pass by degrees from the state of maniacal excitement into that of fatuity, with paralysis, complete or partial—collections by the way which seem to be formed slowly, if we may judge from the increased firmness and density of the parietes of the ventricles, their not collapsing on being evacuated, &c. and which therefore do not prove suddenly fatal. In the one half of the above cases, however, no increased vascularity or effusion could be discovered though carefully investigated. Moreover the treatment which is recommended and usually found successful in cases of meningitis, when applied to insanity, even when the head symptoms appear best marked, is not in general followed by corresponding good effects, but, on the contrary, will sometimes prove highly prejudicial.

*Note.*—These examinations were conducted in the presence of professional friends—this I mention lest it should be supposed that my sense of vision was obscured in obedience to any theoretical bias.

*Aberdeen Lunatic Asylum,*

*9th January,* 1832.

---

### VII.

1. Essay on Cholera, founded on Observations of the Disease in various Parts of India, and in Sunderland, Newcastle, Gateshead, &c. By James Adair Lawrie, M.D. Professor of Surgery, Andersonian University. 8vo, pp. 93. 2d Edition. Feb. 1832.
2. Hints on the Practicability of contracting the Extension and greatly diminishing the Fatality of the Malignant Cholera, &c. &c. By David B. White, M.D. one of the Physicians to the Gateshead Dispensary, and Cholera Hospital. 8vo. pp. 40. Feb. 1832.

The Cholera has spent its force at the towns in the North, from which these pamphlets have emanated, and from which many others will probably spring. When the cholera was said to be coming, our tables groaned with works of all sizes and descriptions, some from men who had seen the disease, the majority from those who know practically nothing whatever about it. The latter were ever the most violent advocates of contagion, and settled its laws, and laid down charts of the march of the malady with most amusing and grave precision. But now, in some parts of the country, the pestilence may be said to have been and gone. We have therefore an opportunity of obtaining some real and sober information respecting this horrid fiend, this raw-head-and-bloody-bones of alarmists in the profession and out of it.

The first pamphlet on our list, is from the pen of Dr. Lawrie, who had an opportunity of seeing the Indian cholera in Bengal, and of comparing it with what he has witnessed in the North of England. Its introduction into Newcastle and Gateshead, and Kirkintilloch, and its actual characters in those towns, form the principal objects of his attention. The first point touched on is, the laws regulating the propagation of cholera. It appears that our author, though always a very cautious and moderate advocate of contagion, has found reason to moderate his sentiments on that head still further. He thinks there is every reason to believe that cholera "is not a very contagious disease." Not a single medical man has been affected with it in Sunderland, Newcastle, or Gateshead, nor have any of the professional visitors suffered, though circumstances would seem to have rendered them most exposed and even predisposed. Typhus, in Scotland, has been infinitely more destructive to our brethren, the larger majority of those dying in Glasgow being supposed by our author to be carried off by this scourge! "Can we avoid the conclusion," says Dr. Lawrie, "that cholera is a much less contagious disease than typhus?"

"Of the nurses and other attendants on the sick, only two have had the disease, and one died. Both cases occurred in Sunderland, and as the nurse who died was one of the very earliest seizures, it is highly probable that she caught the disease, in her own house, and not in the hospital. This remarkable immunity of the nurses strongly confirms the statement, that the disease is not actively contagious, because they sleep, (as I have daily seen them do) in the Sandgate Cholera Hospital, in the same room, and in the next bed to four or five patients labouring under the disease. It also casts a very strong doubt on the favourite doctrine of the predisposition of the poorer classes. The nurses are often taken from the very humblest of these classes, and are generally intemperate; and yet they escape. On the whole, the experience of England satisfactorily proves, that Cholera is a much less contagious disease than Typhus Fever, and that in this respect it bears no comparison with Scarlet Fever and Small-pox."*

This is a point of such importance, that we shall advert at once to Dr. White's opinions on it.

"The hand of charity has, however, been too often paralyzed by the terrors of contagion. What danger may arise amongst the poor, with all their predispositions from exposure to the Cholera, I will not pretend to determine; but of this I am certain, that amongst the class upon which the duties of benevolence must devolve, where the health is good and the mind firm, there is no peril. I have visited the Gateshead hospital during the time I had the honour of being physician to that institution, under all circumstances of physical depression; I have breathed the atmosphere of its apartments for hours together; yet I, the attendants, the nurses, all equally exposed, have equally escaped. Not a single individual in the profession has sustained an attack since the disorder has prevailed."

We shall here make a leap to the concluding part of Mr. Lawrie's pamphlet, in order that all the evidence and opinion respecting contagion may be disposed of in this place. We pass then to the Appendix, in which the mode of origin of the cholera in Kirkintilloch is discussed. Kirkintilloch is a small borough, containing about 4000 inhabitants, situate eight miles North-east of Glasgow, on the North bank of the great canal. Hillhead is a village containing 330 inhabitants, about half a mile from the centre of Kirkintilloch, towards the East, situate on the verge of the South side of the canal.

"Previous to Sunday the 22d January, no peculiar disease prevailed in Hillhead; on that day a boy named M'Millan was taken ill while in church, with symptoms of Cholera, and died early on Monday. Three other persons, Mrs. Semple, Mrs. Kinniburgh, and a boy (Morrison,) were taken ill in rapid succession, and all died before Wednesday. Up to Friday, 3d February, upwards of thirty have been attacked, and eighteen have died. The boy M'Millan had not been on board the vessels on the canal nor come in contact with any part of their cargoes; neither had he committed any error in diet.

The origin of the disease in Kirkintilloch has excited some discussion. At first it was confidently attributed by the authorities and people on the spot, *to animal matters brought by ships from Hull, Edinburgh, and London, for the use of a manufactory in the neighbourhood.* From inspection of the Harbour Master's books, I found that on December 22d, 1831, the Sibilla, Wm. Cowan, from Hull, discharged on the wharf at Hillhead, 3 tons 12 cwt. of horn-shavings, loose; *supposed to have come from St. Petersburgh.* A portion of these was carried to Port Dundas, and in a few days re-shipped to Hillhead, in the Lady Augusta, Sharp. They were soon afterwards removed. On January 16th, the Delight, Primrose, brought *hoofs in hogsheads from London,* which were not emptied on the quay, and were removed on the 18th, four days before the disease broke out. When we consider that the supposed offensive matter from the last vessel was not emptied on the quay, that it came from London, and that the men employed in landing and conveying it to the manufactory, and working it there, have remained in health, there seems no reason for attributing the introduction of the disease into Hillhead to the horn shavings. By some it has been said that Cholera may have been brought from Newcastle, by the regular traders between that town and Port Dundas. On this point I have obtained the following information. The Anne left Newcastle, December 31st, 1831,

* "Before visiting Sunderland, Newcastle, and Gateshead, I considered Cholera to be nearly on a par with Typhus Fever in point of Contagion.—Vide *Glasgow Med. Jour.*"

underwent ten days' quarantine, and arrived at Glasgow on the 14th January, leaving no cargo at Hillhead. She was the latest vessel from Newcastle before the 22d; *the Dulcinea passed on the 29th.* No goods from Newcastle have been discharged on the wharf during the month of January. *The Harbour Master's books show no other source from which the disease could have been introduced.*"

Such is the evidence for contagion and importation in the case of this town and village. What desperate shifts its advocates and the deluded populace are driven to, when they fasten on a cargo of hoofs from London, where the cholera was not, for the importation of the nova pestis into Scotland. But it is of a piece with the other statements of this description.

"If, as there is every reason to believe, the disease was not imported, whence came it? I think we cannot avoid the conclusion that the poison producing it was generated on the spot, by causes similar to those which produced it at Jessore in Bengal, in 1817; adding another proof to the many already before the public, that Cholera can be produced by other causes than a specific contagious virus. If asked why it never appeared before, I can only say that the same causes could never have been in operation, and may retort by asking why it appeared at Jessore (in India) in 1817, instead of 1816, or any previous year. This is in truth confessing our ignorance of the precise cause of Cholera; at the same time there is no want of the general causes of disease at Hillhead. There has long been a disposition in the West of Scotland to bowel-complaints, the weather has been very different from that of ordinary seasons, Hillhead is situated within a few yards of the canal, is damp and dirty, and the people are poor and in want of the comforts and many of the necessaries of life. There is every reason to believe that a combination of causes analogous to these, generated Cholera in India, and why not at Hillhead? Add to this, that the wind for some time has blown steadily across the canal on the village."

The thick-and-thin contagionists scout this reasonable argument; they make the disease originate in one place, but cannot permit it to spring up in another. In the village of Hillhead it was singularly confined to one locality, commencing in the house of a weaver, near the centre of a row which fronts the canal, and being confined to eight houses, all within eighty yards of the first. In these houses sixteen persons died—two others perished who were exposed to their atmosphere—and several within them recovered from severe attacks. To render it probable that this limited locality of the malady is to be explained by endemic causes rather than by contagion, Dr. Lawrie adduces the following facts.

"1st. No communication could be traced between many of the persons who died, and those previously labouring under the disease. Major Berrie's gardener assured me, a few hours before his death, that he had not seen any of the people who had been attacked, nor been in their houses. The man Watson was afraid of the disease, and sedulously avoided those who were labouring under it. 2d, There has been constant communication between Hillhead and Kirkintilloch, but the disease has not, hitherto, appeared in a decided form in the latter place. 3d, Patients labouring under the disease, have been carried from Hillhead to Kirkintilloch, and have died there, but the disease has not spread in the neighbourhood. Of this, there were two examples. After the death of Robert Gardiner, and his daughter, his wife removed, with four children, into the house of Mrs. Horn, her mother, which is situate in the centre of Kirkintilloch. This abode consists of one room, not twelve feet square, in which three or four persons resided, previous to the arrival of Mrs. Gardiner and family. Three of Mrs. G.'s children were labouring under Cholera. The child first attacked died; the two others passed through a severe form of the disease, but not an individual of those residing in the room, or neighbourhood, were infected. I cannot help thinking that the lives of the two children who recovered, were saved by removing them from the house in which their father and sister died. The second instance is that of Mary Brown who went from the centre of Kirkintilloch, to wash the clothes and house of Mrs. Dunn, who had died of Cholera. She remained some hours about the premises, returned to her own house, took Cholera, and died; but not one of her neighbours have been affected. Except the two last mentioned, no death from Cholera has occurred in the town of Kirkintilloch."

These facts seem to Dr. Lawrie to point strongly to a local cause, and to prove that cholera is only contagious within certain limits, well expressed by the name "cholera atmosphere." He thinks that the causes of cholera consist, 1st, Of this peculiar atmosphere: 2d, Of miasmata generated in certain localities, which, under the influence of the atmospherical constitution, produce the disease: 3d, That probably the bodies of those labouring under the disease, or who have died of it, may generate a poison, which, within the range of this atmosphere, may become a cause of cholera. This amounts to mighty little in favour of contagion. By the way, we may here advert to what is asserted by Dr. Lawrie and others, respecting exposure to this atmosphere. "Mendicants from Edinburgh visited places in the neighbourhood, imbibed the local poison, returned and died, but did not communicate the disease to those around them." Thus a small dose of the choleric atmosphere appears to have done for these unfortunate beggars, whilst doctors and nurses have lived and slept with cholera patients, exposed, we must suppose, to the atmosphere, bad as it might be: the medical men have also been in close communion with them *after* death, when their bodies are thought to be capable of generating a poison, and yet neither nurses nor doctors, so exposed and so situated, have suffered from cholera. So difficult is it to decide on the *laws* which regulate its propagation and subject men to its influence. From the opinions and facts already stated, Dr. Lawrie draws the following practical conclusions.

"1st. When Cholera shews itself in any locality, instead of drawing a cordon around it, cutting off all communication with the inhabitants, and confining them to the contaminated spot, *they should be encouraged to leave it and disperse themselves in healthy districts;* their houses should be shut up for two months, and afterwards washed and fumigated."

2d. Dr. L. strongly recommends the formation of cholera hospitals.

"3d. It is exceedingly improbable that the poison of cholera can be carried on the persons or clothes of individuals passing from a contaminated to a healthy atmosphere. From theory, I should say it is *impossible,* and I am not acquainted with a single unexceptionable instance, which can be adduced in support of the opposite opinion.

4th. There is no reason to believe that the poison can be carried in manufactured goods. I call on those who dispute this conclusion to adduce a single well authenticated instance in support of their opinion.

5th. Local quarantines are at once unnecessary and injurious. I trust I have already shown that they are unnecessary, and, surely, I do not require to prove to the manufacturing population of the west of Scotland (in the hope of benefitting whom I have assiduously studied this subject,) that quarantine must be ruinous to the places subjected to its rigor, and injurious to the whole community. Theory tells us that cholera cannot be conveyed in fresh goods, and experience teaches us that quarantines and cordons are powerless in restraining its eccentric, but persevering and uninterrupted march. Why, then, should Governments, proceeding on false principles, inflict on their subjects the unspeakable calamities of local cordons? or why should communities, startled by unfounded fears, injure themselves and their neighbours, by interrupting that free intercourse between districts, on which the well-being of the whole so essentially depends? This subject is highly interesting to all maratime and manufacturing countries, and, at the present crisis, is unfortunately, peculiarly so to the populous districts of the west of Scotland. In refusing to receive goods manufactured in any village in their vicinity, the parties inflict additional privations on the already suffering operatives, and acknowledge a principle, which, by extension, will impose a quarantine on all vessels from the navigable rivers of Scotland. Who can calculate the misery,—the destruction of life,—which such a measure must entail? or could any means be imagined better calculated to multiply the victims of that terrible pestilence, in the hope of checking whose progress, it is likely to be enforced. Restrictions, in every quarter of the globe, have been futile and injurious. What reason have we to hope that they will now prove efficacious? In truth they cannot. I do not ask enlightened mercantile communities to subscribe to the theories of any professional individual; but surely it is their duty, carefully to investigate principles, on whose truth or falsehood the welfare of millions depends."

With the foregoing sentiments we entirely coincide. But we do not expect nor imagine that governments can or will abolish external quarantine; it is an effort of firmness and liberality if they shun internal cordons. Can we fairly look for so much intelligence and courage in governments, when members of our own profession denounce the removal of every shackle as visionary and destructive, and urge on the rabble, high and low, to clamour and persecution against the devoted advocates of common sense? When will the spirit be extinct that animated the torturers of Galileo? Never, whilst sordid jealousy and grovelling ambition are qualities of vulgar minds. The age is not yet sufficiently enlightened for a calm and dispassionate consideration of the question of quarantine.

Having thus disposed of the question of contagion, so far as our present authors touch upon it, we return to the commencement of Dr. Lawrie's Essay, and proceed with our summary of his opinions. The second law which regulates the propagation of cholera, is that it possesses an epidemic character. On this we need not dwell. We may mention some facts respecting that sudden and virulent onset of the disease at Gateshead, which excited attention and surprise.

"Gateshead lies on the right bank of the Tyne, opposite to Newcastle, with which it is connected by a bridge. The population of the town of Gateshead is probably 10,000, and of the whole parish 15,000. The communication between the towns is necessarily very great. Up to the 25th December only two cases of cholera had occurred in Gateshead, although the disease had prevailed in Newcastle for eighteen days. On the evening of the 25th December cases began to be frequent, and by ten o'clock on Monday morning, forty had been seized, and ten had died. At ten o'clock on the 27th, (Tuesday,) there had been ninety-nine cases and forty-two deaths from the Sunday evening. A most intelligent man, a member of the Gateshead Board of Health, assured me, that he had ascertained by personal research, that the disease *had shown itself almost simultaneously in a tract of country three miles and a half around Gateshead.* I visited patients in Gateshead-fell, three miles from the town, on Tuesday morning. *In one small village there had been ten cases and five deaths.* The sudden appearance of the disease in Gateshead was attributed to various causes by those who derided atmospherical influence. The favourite theory was, the dissipation of Christmas-day. Was Gateshead the only dissipated place in England on that day? The low part of Newcastle was equally so, and yet a population of 70,000, presented on the morning of the 27th December sixteen cases, a population of 10,000, forty. It was alleged by many, that the wind suddenly changed on Sunday, and blew during the night from Newcastle. How far this is accurate, I am unable to say; if true, it is possible, that the contagious effluvia may have been borne on the wind from Newcastle, and spread in Gateshead, a soil prepared for their reception. At the same time, when we consider that the alleged change of wind has not been accurately ascertained; that the disease was by no means virulent in Newcastle at the time; and that cholera has frequently spread in opposition to prevailing winds, the conclusion seems quite legitimate, that it affords an instance of its epidemic character."

3d. Our author assigns to cholera a temporary endemic character; i. e. a temporary residence in certain localities to the exclusion of others. Thus, Sunderland, Newcastle, and Gateshead are all situated on the banks of a river, and the poorer inhabitants, who were chiefly affected, dwell in narrow, filthy streets, on the same low banks. Dr. L. contends that the poor and dissipated are not so exclusively chosen by the disease for its victims as is supposed.

"A viewer's family, in every way highly respectable, and comparatively wealthy, was the first attacked at Seghill, and suffered severely. The viewer's house is exceedingly good, of one or two stories, and remarkably neat—but placed close on the bank of the rivulet already alluded to. The pitmen, as I have already stated, are not poor, and not very dissipated; and I met in Gateshead with the disease in young and most respectable females, in whom no personal predisposing cause could be discovered. 2dly. It is a remarkable feature of the Newcastle and Gateshead disease, that very many children, several at the breast, and numbers under six years were attacked with the disease, and that the vast majority of the cases were females. In every locality the females were as two or three to one male, and in some as high as six to one. If the abuse of ardent spirits is to account for the prevalence of the disease among the poor, the proportions ought to have been the reverse. 3dly. The fact already stated regarding nurses, militates strongly against predisposition. 4thly. One of the localities of the disease in Gateshead bears on this subject. Pipewellgate, is a street running along the right bank of the river upwards from the bridge, one side consists of houses placed next the river, the other of houses built against a damp steep bank. Many cases occurred in this street, and almost all of them on that side built against the bank and farthest from the river, — there were very few on the opposite side of the street. 5thly. The greater number of seizures took place during the night and towards morning, a fact more corroborative of endemic than personal predisposition."

4th. Cholera is capable of being borne for distances by the wind. Capt. Dunlop communicated the following fact to our author.

"In Madras Roads, in November 1821, while I was in command of H. M. Ship Curlew, we were lying with several other vessels, well out in the roadstead, which was filled with shipping. In the course of the night, the wind blowing obliquely from the western end of the anchorage, all the vessels in the centre were visited with cholera of the most malignant character, and lost several of their hands, while those ships in shore, and those in the outer line sustained no injury. Cholera was at the time partially on the coast."*

4th. Predisposition occupies Dr. L.'s attention. He thinks that confusion has arisen from susceptibility and predisposition not having been properly distinguished. Susceptibility is that state which renders the individual liable to small-pox, scarlet fever, &c. and is destroyed by one invasion of the disease. This is independent of any perceptible derangement of organs or functions. Predisposition implies such derangement, rendering the body liable to certain diseases, by which it is not only not destroyed, but frequently greatly increased. Dr. L. thinks that, with this understanding of the foregoing terms, neither is strictly applicable to the liability of many of mankind to cholera; a kind of negative piece of information which renders us little wiser than we were. The following will afford a commentary on our text.

"One family in Gateshead consisted of four young women, apparently of sound constitution, and highly respectable; the eldest was in perfect health

* "Letter from Capt. Dunlop, R. N. to the author."

—the second was feeble, in consequence of a late severe illness—the two youngest, the one thirteen, the other fifteen, were in perfect health on the 25th December. The two youngest were seized with cholera on the 26th, the two eldest living and sleeping in the same house escaped. The history of this family is that of a thousand others. Susceptibility and predisposition alike fail us. The explanation must be sought for, by supposing that the two youngest had imbibed a dose of the poison of Cholera, sufficient to produce the disease, while the two eldest had not been exposed to it, or had not imbibed it. At Gateshead-fell, I found in one family the father recovering from an attack of Cholera, one of the family in his coffin, the mother and daughter in bed, ill of the disease. The father told me that he had the night previous been suddenly seized with diarrhœa—he left the house for a few minutes, and on his return three of the family were almost instantaneously seized with Cholera. Before morning one was dead. In a second house, I found two dead and two in bed. The probable explanation is, that all were equally exposed to the same local cause, and that the attacks were independent of predisposition."

One would conclude, from this passage, that Dr. L. conceives that those take it who receive a sufficient dose, and those who do not receive that dose escape. But then how are we to explain the circumstance of the disease being nearly confined to certain classes, and the immunity of medical men and nurses in the north of England? Our author, however, appears to abandon his own position, for, in the next paragraph, he makes predisposition consist in two causes: 1st, The indirect, whatever weakens or injures the health—dissipation, poverty, &c. 2d, Whatever impairs the actions and functions of the organs implicated in this disease, as errors in diet, saline purges, extreme cold. He also conjectures that disease of the mucous membrane may be a predisposing cause. But it is evident to us that, independent of the application of predisposing and exciting causes, there must also be in mankind a degree of susceptibility and insusceptibility, else the disease would not be checked at the limits we have seen, amongst the poor, the wretched, and debauched.

"Does fear predispose to this disease? I do not believe that fear ever gave a man Cholera, or ever will. To the production of Cholera a regular chain of causes and effects is as necessary as to poisoning by prussic acid. The inhabitants of Gateshead fell asleep on the 25th December, in perfect security and devoid of panic, but before the sun rose on the 26th, fifty-five individuals had been seized, thirty-two of whom were destined not to see it set. For several days subsequent to the 27th, the panic of the inhabitants was greater than I have ever witnessed under any pestilence, while the new cases decreased, and on the 30th were as low as 20."

This does not appear to us to be very good reasoning. Dr. L. makes debilitating causes predisponents, yet terror, which is the most enervating of all, is proscribed. Fear, says he, never gave man the cholera. That is not the question. Does it render man more liable to it? If it does not, and that it does we have had on many sides ample testimony, we may venture to affirm that cholera is the first epidemic, in the history of the world, in which the passion of fear has exerted no influence. Dr. L. en passant, alludes to the filth and wretchedness of the English towns which have suffered, and to the greater filth and wretchedness of the Scotch, whose turn is perhaps to come.

Dr. Lawrie makes a few judicious observations on civil police. The aim of Government should be, to open the dense buildings of large cities, prohibit the habitation of cellars, enjoin cleanliness and ventilation, and improve the moral condition of the poor. Aye, there's the rub. How can Government compel landlords to build commodious houses for those who cannot pay for them; how can it prevent over-crowding, and consequent filth, in the abodes of those whose squalid pauperism prevents them from faring otherwise than hogs in styes? Public nuisances, and open exhibitions of filth and nastiness, a Government may prevent by an active police, but private property and private misery have too deep roots in this land of aristocracy and starvation, to be reached by all the powers of Crown and Parliament.

Dr. L. strongly recommends the formation of a hospital *before* the disease is established, for if delayed till then, it is created in panic and confusion, and is productive of little benefit.

" Every town, in which there is a sufficient number of medical men, should be divided into districts, and medical men put in charge of them; and an hospital or hospitals, according to the size of the town, provided in healthy situations. A private dwelling, schoolroom, or dry barn may speedily be converted into a commodious place for the reception of Cholera patients. If possible, it should have two sets of wards, one for the reception of those in the state of collapse, a second for those in the febrile stage. The furnishings should consist of wooden bedsteads three feet wide, mattresses made of coarse cloth stuffed with kiln-dried straw, sheets, and abundance of blankets. When a patient dies, the straw should be burnt and all the bedding washed. Each ward for the reception of patients in the stage of collapse, should have a stove and a large fire-place to keep up a high temperature, and enable the nurses to procure with facility the means of applying external heat. In this disease, assiduity will often supply the place of skill, and proper measures are more likely to be adopted, when the means of applying them are at hand, than when they require to be sent for. If possible, each hospital should contain accommodation for a resident house-surgeon."

The observations on the proximate cause of the disease must be passed over; in fact, we fear we have devoted already too much space to this pamphlet. We proceed to the consideration of the symptoms and treatment, on which we shall be as brief as possible. Cholera is divided by Dr. L. into common, spasmodic, and malignant, all belonging to the same order of disease, met with in the same epidemic, differing only in degree. Passing over the two first, we find that Dr. L. divides the malignant cholera, as he saw it in the north of England, into five stages; 1, The premonitory: 2, The acute: 3, The collapsed: 4, The rallying: 5, The febrile.

The premonitory consists essentially of diarrhœa, with a slight degree of the cold stage, malaise, and perhaps spasmodic twitchings of the limbs. This stage varies from a very few hours to two, four, or even six days. The acute stage is always rapid, though varying in its symptoms. In some, it consists of a very sudden increase of the preceding symptoms, and the stage of collapse speedily supervenes. In others, there is vomiting as well as purging, acute spasms, thirst, often acute spasm at the pit of the stomach, and almost constantly a burning, unquenchable, gnawing sensation; the pulse continues good, and the surface is not very cold. The face becomes more sunken and anxious, and the patient is very restless. Respiration is rather slow, the secretions are diminished, that of the urine suspended, the skin clammy, sometimes perspiring profusely, the voice husky. A vein opened yields blood freely. This stage may be very short, or some hours may elapse before collapse be fairly established. In some instances, the attack is instantaneous, the patient falling senseless, and recovering to be plunged into more prolonged agony. The fact is, that the modes of attack are various, and the symptoms differ in different cases. Our author gives a powerful and graphic description of the cold stage; we regret that we cannot convey it to our readers, but our space is so limited, and this stage has already received so much and such general attention, that it is unnecessary to dwell upon it.

" The majority of medical men affirm that the mind is always unimpaired. I can only say that it appeared to me in many cases affected, to a degree little short of what we find it in concussion of the brain, probably owing to congestion in the vessels of the head. I remarked this particularly in several children. When undisturbed they lay in a dull sleepy semi-comatose state, breathing with perfect calmness. When roused, they thought only of relieving their distressing sensations, and vociferated for cold water. I had at one time five of the same family in one bed, all calling loudly for fluid to allay their burning thirst. So urgent were they, that they could be bribed to swallow any medicine, by the promise of a mouthful of cold water. When appeased, they relapsed into their previous sleepy condition.

It is hardly necessary to say, that confirmed collapse is a state of extreme danger, and that it is it which destroys the vast majority of Cholera patients. The cause of death, in many cases, is a failure of the functions of animal life alone; in others, affection of the brain, induced either by venous congestion or the absence of arterial blood, hastens the fatal termination.

I am borne out in this last view of the cause of death, by the fact, that, although the powers of animal life are first affected, they are in some cases the last destroyed. Convulsive movements are common after mental life is quite extinct; and, as I formerly remarked, it is almost impossible to say at what moment the vital motions have ceased to vibrate. A body lies apparently lifeless, suddenly a convulsive shudder shakes it; its hands are clenched, if you put your own within them, and force them open, they shut again with a spasmodic catch. It is not to be wondered at if these appearances have a powerful effect on the superstitious feelings of the uneducated, and make them averse to very early interments."

The 4th stage is that of rallying. Sometimes, but these are the exceptions, it is complete and rapid; usually it is of some hours' duration, nay, even of two or three days, and the animal powers seemed placed in a delicate balance. In some of the most rapidly fatal cases, Dr. L. has seen the heat partially return to the surface, the pulse flicker, and sensation slightly restored, immediately before death.

"5th. *Febrile Stage.* A patient who has entered the stage of collapse, is rarely restored to health, without passing through a fever closely resembling typhus. My residence in Newcastle was too short to enable me to say from personal observation, what is its usual duration, but I understood that it varied from seven to fourteen days. The ordinary cases present no peculiarity of symptoms, which could distinguish them from those of common continued fever. At first I thought the tongue cleaner, and the pulse less rapid; but more extended observation showed, that these, especially the clean reddish tongue, are occasional, but by no means invariable symptoms.

Congestion of particular organs is met with at the commencement or during the progress of the febrile stage. By far the most common is that of the vessels of the brain, marked by apathy, listlessness, dilatation of the pupils, suffused eyeballs, low muttering delirium, or total insensibility. The fauces, in some cases, in which the voice had been much affected, inflamed, and suppurated. Inflammation of the brain followed one case of inflamed throat. The chest I rarely found affected. The respiration in the febrile stage is usually healthy, and the lungs free from congestion. I do not pretend to say that affections of the chest have no place among the sequelæ of Cholera, but that I have not met with them.

"Congestions and inflammatory affections of the abdominal viscera are very frequent; indeed it seldom happens that the functions of these organs are speedily restored. Under proper treatment the liver is not very obstinate. I thought affections of the stomach, probably its mucous surface, as indicated by troublesome retching and vomiting, and of the bowels, in the shape of constipation, fulness, and pain on pressure, much more common. The secretion of urine is restored, the skin assumes its ordinary hue, and attains a febrile heat. The recovery is progressive, as from continued fever."

Dr. L. considers the disease to be the same as the Indian cholera, but characterised by certain peculiarities which he enumerates.

"First, The premonitory stage occurs in a much larger proportion of cases in the English than in the Indian disease. In the latter it was the exception, in the former it is the general rule.

Second, The febrile stage is incomparably more frequent. I have met with it in India, but it generally appeared to me to be connected with derangements of the abdominal viscera. In Sunderland and Newcastle the experience seems to be, that very few who have fairly entered the collapsed stage, escape the secondary fever.

Third, I think the head is more frequently affected in the British disease than in the Indian—as indicated by greater mental oppression and insensibility during the collapse, and more congestion in the febrile stage. In this last particular, I believe I differ from some other observers. I speak, however, of my own experience only.

Fourth, My observations would lead me to say that the frequent discoloration of the surface constitutes a fourth peculiarity. I neither met with it so frequently, nor to the same extent in India."

We pass to the treatment. Both Dr. Lawrie and Dr. White insist on the necessity of attending to the premonitory, or diarrhœal stage. Dr. L. recommends the patient to be put to bed, kept warm, and to have a flannel band round his abdomen—then to take half an ounce of castor oil with fifteen or twenty drops of laudanum, and, after this has operated, thirty drops of laudanum, or one grain of opium. Saline and drastic purgatives are strongly deprecated. If the discharges continue, the opiates must be repeated with caution, and if two or three grains of opium fail, Dr. L. would open a vein, and in every case apply a sinapism or blister to the abdomen. If the above measures fail, and collapse threaten, he advises a mustard emetic, to be followed up by twenty grains of calomel, and as much laudanum or opium as the patient will bear. Opiate enemata deserve trial, and if opium has been pushed as far as is safe, mucilaginous enemata should be thrown up with a Jukes' syringe.

Dr. White remarks that in some cases, not diarrhœa, but obstinate costiveness prevails, after a longer or shorter duration of which, the disease suddenly appears. For this costiveness he seems to recommend a pill of scammony, jalap, calomel, and aloes. In this stage Dr. W. also advises the washing of the body with warm water, and subsequent friction with coarse cloths. He seems to think that personal filth is a great predisposing cause. He orders his patients, on their retiring to bed, the lavation and rubbing—then the pediluvium and a pill of cal. prep. gr. vi. opii, gr. j.—in the morning castor-oil and laudanum, or pulv. rhei, ʒss. zinzib. pulv. gr. viij. to be repeated if necessary.

*Treatment of the Acute Stage.* Dr. Lawrie recommends a mustard or salt emetic, the former not too strong; he doubts their efficacy or safety when profuse vomiting of colourless fluid is a prominent symptom. As soon as the emetic has been swallowed a vein should be opened. His experience is in favour of bleeding, and, as a general rule, the earlier the better. In some cases it undoubtedly appears to do harm, and it is difficult to explain this discrepancy of effects. We may say that the earlier the better, that it is more safe in the robust, probably more safe after collapse than when it is just on the point of being established, and in the moderate than in the very severe cases. Such are Dr. L.'s notions. After these means we are to give a large dose of calomel and opium, to a stout man thirty or forty grains of the former within two hours. Of opium a large dose should be given at first with the calomel, and its repetition should be regulated by circumstances. Dr. L. strongly recommends counter-irritants to the throat, neck, spine, to the latter indeed the cauterizing-iron. Stimuli should also be given internally, if the stomach will retain them.

Dr. White thinks bloodletting has been too universally recommended. When vascular debility is very apparent he would not employ it, but when the pulse retains its force or rises during the operation, it is advantageous. Bleeding too he thinks an admirable sedative, when great mental excitement prevails. He recommends the exhibition of cold water slightly coloured with brandy. He speaks well of cupping, external warmth, frictions for the spasms. He has found nothing but disappointment from the exhibition of stimulants; but speaks very highly of large injections of warm water.

*Stage of Collapse.* Dr. L. recommends warmth—an emetic as before—the abstraction of a few ounces of blood while the pulse can be felt in the brachial artery—then warm injections of beef-tea with brandy and laudanum—counter-irritation—diffusible stimuli—cold drink in its most agreeable form, for which, and for which only, the patient craves. All fluid should be given in small quantities at a time; thin arrow-root or beef-tea often remain on the stomach when nothing else will. External warmth is to be maintained by stoves or large fires in the room.

"A very simple method of heating a room, is an iron tube bent at its centre, the bent part converted into a small reservoir or worm. The reservoir is put into the fire and heated red hot; to the one end of the iron tube another of tin or of patent cloth is attached, and to its extremity a pair of large bellows. Air is thrown into the reservoir or worm, becomes heated, escapes at the opposite end, and is diffused through the room. One of this kind has been described to me; the principle might easily be improved into a very efficacious means of raising the temperature of a room or bed, to any required height. The air-bath is another means of applying heat to the surface generally, but air being a bad conductor must not be trusted to alone. Tin cases containing hot water, light and shaped to apply to any part of the body, are very useful for applying heat locally; to the same class belong bricks, bags of heated salt or sand, hot plates, and many others. My friend, Mr. Brady, at Gateshead, was in the habit of using bran mashes, for communicating heat to the surface, with good effect. A quantity of bran is put into a tub, and boiling water poured into it in such quantity as merely to moisten it. It is then thinly spread in a bag sufficiently large to cover the whole body, and applied from the neck to the feet. It must not be applied too hot. This mode has the advantage of retaining the heat long, and is one of the best forms of moist heat which I know. If we wish to excite the surface powerfully, we may add spirits of turpentine to the bran. It is exceedingly difficult to prevent the patient from tossing in bed, withdrawing himself from the heated bags, and throwing off the bran and bed-clothes. A nurse must be constantly beside him to prevent this as much as possible."

When the perspiration is profuse and cold, it should be carefully wiped off with dry hot towels, and afterwards camphorated or ammoniated oils should be long and diligently rubbed on those parts where irritants are not applied. When the head and face remain deadly cold while the trunk and limbs are heated, it is a very bad symptom. It is singular that the patient dislikes the application of heated cloths, and begs for free *cool* air. Dr. Lawrie recommends the employment of galvanism, the agent to which we ourselves directed attention in our last number. He advises the application of the wire along the course of the eighth pair of nerves in the neck, and over the region of the heart and stomach. Dr. L. would be inclined to give calomel in moderate doses along with stimuli. Other means were employed with doubtful results Oxygen gas was given both by the mouth and by injection, but with very little benefit. It was recommended to throw large quantities of heated air into the bowels, but Dr. L. does not seem to have seen it attempted. Stimulating enemata of sp. terebinth. and mustard were used by some; they frequently produced irritation of the rectum and bloody stools; Mr. Baird, of Newcastle, used the tobacco enemata in one case of collapse with seeming benefit. Dr. L. tried it in two cases: in one it did no good, in the other it appears to have done harm. We think it a dangerous remedy, not warranted by principle and reasoning. When dogs are poisoned by tobacco injections the symptoms are precisely those of cholera:—purging, vomiting, cessation of the pulse, complete collapse. How such an agent should be likely to prove beneficial in cholera, we cannot so much as imagine.

Dr. Lawrie observes, that in the stage of collapse nature does and will do nothing; perhaps the expression is too strong. If the following fact be one, it is instructive. A medical man left a cholera patient, saying that he could do no more for him, and that he must die; another surgeon passing by accident, was asked to see him, worked hard for four hours, and saved him!

Dr. White's remarks on the treatment of this stage are brief.

"I have found the simplest, the most successful treatment;—the calomel and opium, the carbonate of ammonia, warm injections, stimulants as cordials, friction where necessary, and warmth, especially of a dry kind; worsted stockings filled with hot sand, as I learnt from the practice of Mr. Knaggs of Gateshead, is admirably adapted for this purpose.

To allay the vomiting, as in the former instance, effervescent draughts, &c. should be used. A most salutary and speedy application, is by laying over the stomach pledgets of linen, dipped in boiling water. Whatever may be the plan adopted, all unnecessary disturbance to the patient should be avoided. The death-bed is often rendered more agonizing by the mistaken, but well-intentioned, zeal, the benevolent officiousness, of inexperienced practitioners. An enema, consisting of a warm infusion of tobacco, half a dram to a pint, has been recommended by Mr. Baird. I have seen recovery in two apparently hopeless cases, in which this remedy had been employed."

Dr. W. makes a remark which is highly deserving of attention. When cholera first rages its inveteracy is greater than at a subsequent period, and remedies which utterly fail in the first instance, acquire a renown and celebrity when nature herself is at work to assist them. Dr. White speaks slightingly of mustard emetics; he has never seen them serviceable in the collapsed stage, whilst in some instances they were incontrovertibly mischievous.

"Prior to death, when it takes place during this stage, the disease sometimes assumes a form, well calculated to deceive those not acquainted with its character. The pulse rises, the skin becomes warm and covered with perspiration, the patient expresses his relief, and, perhaps, his full anticipation of recovery: in an hour or two all is over. The blueness, so peculiar to this disease in other countries, has not been, by any means, a general characteristic in this. In not one instance in ten, has it assumed that form. The skin of the hands and face often becomes of a brownish hue. In the most deadly form of cholera, there is a tone of voice, a wail, which once heard, can never be mistaken; to him, upon whose ear it has fallen in the accents of anguish, it can never be forgotten; I have always found it the certain prognostic of death."

Here we see that the blue colour, the true blue, as the papers sarcastically term it, is not present in one case in ten. Yet this true blue is one of the pathognomonic symptoms that the ultra contagionists, that is, the thick-and-thin importation and nova pestis party, hold by as their sheet anchor. But what signify facts? At the time we are writing, the 25th of February, the epidemic is stealing over this metropolis. We have seen many, indeed most of the cases that have hitherto occurred, but in no one instance could our eyes, unassisted by the spectacles of quarantine notions, and the mists of party feeling, discern any shade of blueness. That colour is seen in its deepest dye in the visages of contagionists and sycophants.

*Treatment of the Rallying Stage.* In this the surgeon is to watch nature; to prevent the recurrence of collapse on the one hand, and ward off congestions of the head and viscera on the other. The first is avoided by external warmth and by giving warm arrow-root in moderate quantities; the second by small doses of calomel, taking care not to push it too far. The head should be shaved; when the stomach continues very irritable, blisters and effervescing draughts with small quantities of laudanum are very beneficial.

*Treatment of the Febrile Stage.* When congestions do not occur, a few grains of calomel and small doses of castor oil and acidulated drinks are generally sufficient. In common cases the lancet is unnecessary, and when congestion threatens it is a hazardous remedy. Dr. L. mentions a case in which it appeared to re-induce collapse. When congestion threatens the head, which is the chief danger, blisters to the scalp or nape of the neck, preceded by leeches and cold lotions, and followed by calomel and mild purgatives are proper. The same principle applies to abdominal congestions. The patient often dies with the worst form of typhus.

Dr. White remarks in this stage the redness of the tongue; he thinks this is the most proper time for the employment of the lancet with proper precautions; when there is obstinate greenish sickness he has seen great benefit from the salt emetic, repeated till nothing but the solution is rejected. He draws attention to the peculiar tendency to affection of the head, insidious in its progress, and fatal in its tendency.

Dr. Lawrie concludes with a sort of summary of the history of the cholera as it has hitherto appeared in this country, and of the mortality relatively to other countries.

"Great variety has obtained in different parts of the world, in the proportion which those attacked with cholera bore to the population, and those who died to those who were seized. In some towns of Syria it destroyed one half of the population; in Tripoli only 1 in 3,000! In India, the proportion of the troops exposed to those attacked, varied from 1 in 10, to 1 in 20; and the deaths, from 1 in 3, to 1 in 6 of those seized. Among the native population, many of whom were devoid of the necessaries of life, and all of medical aid, the mortality ran as high as one half or two thirds. In Russia, according to Moreau de Jonnés, in five months, 1 in 210 of the population exposed were attacked, and 1 in 350 died. The deaths amounted to three-fifths of the seizures.

In England and Scotland, from October 26th, 1831, to February 4th, 1832, 3,500 have been afflicted with cholera, and 1080, (less than one-third) have died.

In Sunderland, from which town the disease has now disappeared, 533 have been seized, and 210 died, affording a mortality of upwards of one-third. I have found some difficulty in ascertaining the amount of the population of Sunderland, but conceive that the town itself does not contain more than 20,000. On this calculation the seizures are as 1 in 37$\frac{1}{3}$, and the deaths 1 in 95, of the population. We cannot arrive at any accurate conclusions regarding Newcastle, because the disease still prevails there; up to the 12th January, however, the deaths are less than one-third of those attacked.

In Gateshead, from December 25th to February 3d, 399 have had cholera, and 142 have died; the deaths continuing steadily more than one-third of the seizures. The parish contains 15,000 inhabitants; consequently the seizures are more than 1 in 38, and the deaths rather less than 1 in 105 of the whole population.

From these data we may draw the following conclusions:—

*First.* The numbers attacked, in proportion to the population of the places infected, are more than five times greater in Sunderland and Gateshead than in the Russian dominions; and the deaths to the population more than three times. The seizures are nearly one half less than among our troops in India.

*Second.* The proportion of deaths to seizures is 1$\frac{1}{2}$ higher than among our Indian troops, and considerably lower than among the Russian peasantry.

*Third.* When we consider that this disease is limited to the inhabitants of certain localities, its effects on families must be fearful. It is no uncommon occurrence to meet with two, six, or even eight of the same family stricken with it, within a few hours. The picture is aggravated when we recollect that the disease selects its victims from that class of the community, who are least able to sustain any addition to their misery.

*Fourth.* The statement, that cholera does not increase the bills of mortality, is not correct. The average number of deaths in Gateshead was nearly eighteen monthly. In that parish the deaths from cholera alone, in nineteen days, were $7\frac{1}{3}$ times that amount."

Here we must bring our analysis of these pamphlets to a termination. We have given a faithful account of the opinions and conclusions at which their authors have arrived. They are valuable, because they are the result of experience on our own shores, because they are the inferences drawn from the phenomena exhibited by the disease as it exists among ourselves, and because they present us with the sentiments of candid and unbiassed men. To say that they support the opinions we have consistently and conscientiously advanced is nothing; but it is more to be enabled to assert, that they give weight and confirmation to the doctrines of moderate men. They contribute to establish the idea, that cholera is far from being so contagious a disease as to warrant those internal and external restrictions on commerce, which the Continental nations have hitherto universally adopted. They also support the doctrine of contingent contagion, that is, of contagion fostered, if not generated, in personal filth, unwholesome localities, and over-crowding of the famished, the miserable, and the dissolute. To assert that the disease is absolutely and unconditionally confined to such persons, is proved to be incorrect, but the general rule which makes it select such for its victims is no less fully established.

Men, sincere, perhaps, but ill-judging, have ventured to connect the extension of this too destructive malady with the special interference of that all-powerful Being, whose decisions are as wise as they are inscrutable. Far be it from us to speak lightly on a matter so serious, or on a subject referring to a source so awful. But we may be permitted to observe that it is presumptuous, at the least, for any man, or any set of men, to attribute this or that infliction of evil to the particular wrath and interposition of the Deity. In the present instance, it is peculiarly dangerous thus—

To deal damnation round the land
On each we judge His foe!

The classes chiefly afflicted by God, are those already ground to the dust by man. The wretched, starving artisan: the blighted, shankless labourer: and their famished, half-clad families: these whose food consists of bad bread and potatoes—whose drink, of solutions of herbs which pass for tea, scorched grain for coffee, and of gin, when they can procure it; these, and such as these, are the victims selected by man for Heaven's displeasure. Alas for the oppressed and pauper population of this Empire! They are marked by misery and disease for their own—and the rich man wraps himself in his furs, and hugs himself that it is not he whom the Divinity has selected for extermination.

Before this article has seen the press, the practitioners of London will have had the opportunity of comparing their own experience with that of their brethren in the North. The epidemic is upon us; the modern Babylon, with its fifteen hundred thousand inhabitants, its squalid poor, its wretched alleys, its filth and splendour, misery and magnificence, is shadowed by the wing of the Angel of Death, and under the ban of the rulers of kingdoms. The cholera is in the mightiest city ever visited by pestilence; years have rolled away since its inhabitants were decimated by plague and fire—the affluent have found new comforts, but have the poor been gainers? Events will determine the lot of the metropolis of the world.

---

### VIII.

AN ACCOUNT OF THE BEULAH SALINE SPA, AT NORWOOD, SURREY, &C. By GEORGE HUME WEATHERHEAD, M.D. Octavo, pp. 39. 1832.

IT appears, by this little brochure, that the citizens of London have a good substitute for Cheltenham and Leamington within seven miles of Modern Babylon, above the fogs of the metropolis, where the air is pure, and the water highly medicinal. The Spa lies embosomed in a wood of oaks, open to the south-west, on a gentle acclivity, and commanding a very extensive and variegated prospect. The following extract will give our readers a knowledge of the chemical characters of the Beulah Spa, as compared with the Cheltenham pure saline springs.

"The water drawn fresh from the well is beautifully transparent and sparkling. Innumerable bubbles of fixed air are seen rising to the surface, when allowed to stand. Its taste is distinctly bitter, without being at all disagreeable, leaving on the palate the peculiar flavour of its predominant saline ingredient, the sulphate of magnesia. The temperature of the water, at the bottom of the well, is 52° of Fahrenheit; its specific gravity 1011; and, by an analysis of its composition by those distinguished scientific chemists, Messrs. Faraday and Hume, the following are the solid contents of a quart of the water:—

BEULAH SALINE.

| | |
|---|---|
| Sulphate of magnesia...... | 123 |
| Sulphate of soda and magnesia ................ | 32 |
| Muriate of soda .......... | 19 |
| Muriate of magnesia . .... | 18½ |
| Carbonate of lime ........ | 15 |
| Carbonate of soda ........ | 3 |
| Grains. .. | 210½ |

CHELTENHAM PURE SALINE.

| | |
|---|---|
| Sulphate of magnesia ...... | 22 |
| Sulphate of soda .......... | 30 |
| Muriate of soda............ | 100 |
| Sulphate of lime .......... | 9 |
| Grains.. . | 161 |

As a mean of comparison, the saline contents of a quart of the Cheltenham pure saline, as analysed by Mr. Brande, the predecessor of Mr. Faraday in the professorship at the Royal Institution, is placed opposite to the Beulah Spring, to enable the reader to judge how much superior, as an aperient water, the latter is to that of Cheltenham. And, first, it may be observed, that the gross amount of the several salts, in the same quantity of the waters, is much greater in the Beulah than in the Cheltenham Spring, the difference being forty-nine grains and a half of solid saline matter in a quart—that is, the impregnation is nearly one-third stronger; and, secondly, the nature of the saline ingredients also merit observation. One hundred grains, out of one hundred and sixty-one, consist, as we see, in the Cheltenham, of muriate of soda, or common table-salt. Now, this substance, when perfectly freed from other salts adhering to it, possesses comparatively very feeble aperient properties; whereas the mass of the ingredients in the Beulah Spa is composed of two powerful saline substances, the sulphate of magnesia, and that peculiar double salt, the sulphate of soda and magnesia, constituting three-fourths of the whole saline impregnation."

We understand that a commodious hotel is erecting, in the neighbourhood of the Spa, for the accommodation of invalids: and we should not wonder if the Beulah waters attain a considerable celebrity, on account of their vicinity to the metropolis.

---

### IX.

CHOLERA, AS IT HAS RECENTLY APPEARED IN THE TOWNS OF NEWCASTLE AND GATESHEAD; INCLUDING CASES ILLUSTRATIVE OF ITS PHYSIOLOGY AND PATHOLOGY, WITH A VIEW TO THE ESTABLISHMENT OF SOUND PRINCIPLES OF PRACTICE. By T. M. GREENHOW, (of Newcastle-upon-Tyne,) Member of the Royal College of Surgeons in London, &c. &c. &c. Octavo, pp. 162. London, 1832.

THE Cholera is in this city, and if any thing can compensate for this pest of the poor, it is that the fire-side travellers who prated so complacently of its whereabouts, have been the first to be attacked with the cold tongue, blueness, shrunken features, and other characteristics of its stage of collapse. The ultra-contagionists are in a stage of congestion too deep, we fear, to be remedied by any stimulants. Time was, and the weeks that have slid away since that halcyon period are but few, when we saw them confident and triumphant in the public places. The societies then were their's, and the press was their's, and the public voice was with them, and the public panic was a pleasant thing to see, and who so elated as the contagionists then! The great men were very great, and their dependants were very happy, and in the fulness of their hearts they did what all other zealots have done, they denounced man's vengeance and heaven's displeasure on those whose creed was not fashioned by their own. The moderate party, if a party moderate men can ever form, were held up to scorn and derision and insult, and their opponents openly thanked God, as the Pharisee did, that they were not like to them. But the plague was in the land, and men used their senses in deciphering its characters, and the mist began to clear, and reason and truth were more distinctly seen. But ah! it was a sorry sight to look upon the ultra-contagionists, and a sore change it was that came upon their fortunes. They shunned the societies where victory had been their's, and the press, to which they had heretofore flown with such glee, was a sealed thing to them, and they loathed the light of the day because it was too bright, and they wrapped themselves in the dignified and deep obscurity of their closets.

The leaders gave the cue, and their followers, like the sheep of Panurgus, caught it. It was criminal and wrong for men to investigate the malady that was scourging nations, it was right and proper to shut their eyes and hold their tongues. This was the new creed, this was the novel and aristocratic method of deciding disputed or clearing dubious points, in a disease confessedly mysterious. It must be owned that the mode was not wholly original, an ancient and homely adage on opening the mouth and shutting the eyes, having probably furnished a hint to those who shewed themselves apt in taking it. But the bile, which poor wretches with cholera cannot shew, was amply secreted by the ultra-contagionists, nay, it was not only secreted but discharged, and very green it seemed to the bye-standers to be. Again was their virtuous indignation excited by their opponents, again were the latter derided and insulted, again was their foolish zeal contrasted with the eminent placidity and quietism of men who took fees or could not get them regardless of cholera, again were the vials of wrath of man and Deity vented openly on their heads, again was it lamented that a mob could not be raised to wreak on them perchance what a mob once wreaked on Priestley. Some foreign reader may think this exaggeration and caricature. Alas! it is not. We blush for our profession, when we are necessitated to confess that every tittle of what we have merely hinted, is graven in characters too black to be effaced. But we leave this painful subject for the present.

To return then to the march of cholera. Wonderful to relate, those who traced it, inch by inch, from Jessore to Hamburgh, with as much precision as a courier's route could be traced by the changes of horses and the "traveller's book" at the hotels, have all at once lost their clue, and found themselves unable to track the monster, even in a single instance, between the Thames and the Clyde! We, indeed, find no difficulty in the solution of this problem, from a firm conviction that nine-tenths of the facts and records by which our literary practitioners tracked the cholera, are not merely unintentional errors, but, in a great proportion, wilful and deliberate falsehoods, based on political creeds or hypothetical prejudices! The present number of our Journal will, we fear, contain some strong proof that, even in our own country, where a line of policy is to be supported, or a doctrine to be maintained, truth is voted a bore, and no facts or arguments listened to, except when on one side of a question.

In this land of freedom, however, there is yet some independence of spirit—some immunity from the thraldom of authority—and some medical practitioners capable of observing for themselves, and resisting the dictates of medical coteries, when in opposition to the evidence of their senses. With joy, therefore, do we abandon the mephitism of literary compilers, and turn to the results of experience in our own island. Instead of the horrible and unspeakable names, Russian and German, whom we have been consulting for a year or two past, respecting cholera, together with the dressed-up and poetical (query political) portraits presented to us, we have now the plain unvarnished tales of English practitioners who have witnessed the epidemic on our own shores—and we have the evidence of our own senses, as tests for both foreign and domestic statements. Truth will now come out—and delusion has just cause to fear. But of this more anon.

Mr. Greenhow's book is one of the most interesting that we have perused since that of Mr. Bell. It is distinguished for modesty, (almost always an attendant on talent,) clear conception, and accuracy of judgment. We regret exceedingly, that the late period at which we received the publication and the multiplicity of subjects on which we were engaged at the time, will prevent us from doing justice to the work. We trust, however, that no practitioner who has any zeal for his profession, or interest for his own success, will fail to possess himself of such a cheap and valuable book. It is worth all the *literary* effusions of the medical press put together on the subject of cholera.

Mr. G. demurs (and well he may) to the name of the disease. If it had any thing to do with bile at all, it ought to be called *acholia* instead of cholera. He thinks that "malignant cholera" is the least objectionable term. We think the term "acholeric fever" would be better. Mr. G. confirms the observations of others that the asphyxial stage is generally preceded by gastro-enteric irritation—usually in the form of diarrhœa—and that a fever succeeds in proportion to the intensity of the asphyxial paroxysm. It is in the second stage only that the disease can be recognized from common diarrhœa and fever. The following is an analysis of the symptoms.

"1. Great irritation of the minute nervous expansion of the stomach and bowels, whereby they are impelled to a frequent discharge of their contents.

2. A peculiar condition of the mucous membrane of these organs, whereby their proper absorbent and secretory functions are entirely suspended, and they permit the profuse serous discharges to drain through them without resistance.

3. When the serous or watery discharges from the stomach and bowels have become once fairly established, a complete suspension of the biliary and urinary secretions takes place.

4. A failure of the action of the heart and arteries, often so complete as to amount to a total stagnation of the circulating mass, not only in the capillary but in the larger vessels; while the blood is changed in quality by the failure of the process of oxygenation and the removal of its thinner parts, and diminished in quantity by the profuse serous or watery discharges from the stomach and bowels.

5. Consequent and dependent on the arrested circulation, a complete failure of animal heat, as evinced by the cold extremities, and the extraordinary coldness of the tongue and breath.

6. Spasmodic actions, more or less severe, of the muscular apparatus—probably dependant on sympathy with the irritable condition of the alimentary canal."

Mr. Greenhow enters on an ingenious physiological examination of the phenomena of the disease. We will not affirm that such is useless, but although we cannot altogether agree with our zealous author, we cannot afford space for either his opinions or our own comments. The following extract is more immediately practical. It comprises a good abstract of the cadaveric changes from cholera.

"No alterations either in the organization of the neuralgic, or visceral structures, has been discovered to account in a satisfactory manner for the remarkable train of symptoms which characterizes this extraordinary disease, and any morbid appearances which have presented themselves must be considered rather as arising from, than as having occasioned, the functional derangements which were observed during life. The truth of this remark, I think, will be corroborated by the writings of every author on the subject, and certainly my own observations have tended entirely to confirm it. In point of fact, with the exception of a distended gall-bladder, and an empty shrunk condition of the urinary bladder, which have very constantly been observed, no morbid appearance whatever has been discovered, but what might fairly be anticipated as necessarily arising from the arrest of the circulation during life, and altered condition of the blood already noticed. The blood is found dark and viscid, and collected in the great vessels, congested in the liver and lungs, and probably injecting the vessels of the mesentery and intestines, of their mucous membrane more especially, but occasionally of their serous membrane also. The vessels of the brain and spinal cord have sometimes been found in the same injected condition. We can easily understand how such a vascular condition of viscera should, in case of re-action being established, *lead to extensive and acute inflammations, as so frequently happens when the patient passes into the third stage of the disease; and if re-action have fully or partially taken place at the time of death, we shall in all probability find ample proofs of the existence of such inflammation.*"

The following are the indications laid down by our author in the treatment of the malady.

"1. The necessity of allaying irritation in the nervous expansion of the stomach and bowels.

2. To excite the vascular system, and to restore animal heat.

3. To restore the suppressed secretions.

4. To obtain healthy evacuations from the bowels and kidneys.

5. To moderate re-action, and obviate congestions, local determinations, or organic inflammation.

The first indication may be considered as common to the first and second stages of the disease, the three succeeding ones relate principally to the second stage, and the last indication is peculiar to the third stage of cholera. We will consider them in the order of their arrangement."

In our notice of these works on cholera we have rather departed from our ordinary practice of analysis in preference to quotation, because it is a disease which is not more characterised by the peculiar symptoms well known and often described, than by the epidemic tendency to falsehood with which it seems to infect many of those who write upon it. If we did not give the very words of an author, ten to one that we should be accused by the ultra party of misrepresentation. In the first stage Mr. G. recommends emetics and bleeding, under the following circumstances.

"If the stage of collapse have not yet established itself, and if with bilious diarrhœa the patient complains much of nausea and occasional retching, the matter rejected consisting principally of undigested food, we shall probably find a dose of ipecacuanha, with or without antimony, answer the purpose, or even copious draughts of warm water will suffice to wash out thoroughly the contents of the stomach. Should the patient complain at this time of vertigo, head-ache, or pain in the abdomen, with an accelerated pulse, the greatest relief will arise from a full bleeding, proportioned, however, to the strength of the constitution, the effect produced upon the pulse, and the urgency of the symptoms. As the blood flows the pain in the abdomen and head will be relieved, and the pulse will become slower and softer; a manifest diminution of gastric irritation will also result from it."

When the stage of collapse has set in we require stimulants—the mustard emetic—after this doses of calomel and opium, the latter not in too large quantities, grain doses being generally sufficient — internal stimulants, brandy perhaps the best—copious warm stimulating injections into the intestines—external heat and friction. Mr. Greenhow makes the following remarks on the application of the latter.

"For the accomplishment of this purpose, one of the first things to be attended to, is to clothe the person of the patient in a large body-dress or shirt of thick flannel, and to envelop him, when laid in bed, in an ample supply of warm blankets; heated bodies of any convenient description should also be constantly applied to the extremities, to the spine, and to the pit of the stomach and abdomen. Various contrivances have been made use of for applying heat to the surface. To the employment of the warm or hot bath it has been objected, that the exertion and fatigue attendant on its use, are likely to be more injurious by the exhaustion they would occasion, than could be compensated by any benefit arising from the general application of heat that would be obtained; and although warm baths have been found beneficial in India and on the Continent, in this place, they have not been resorted to in the treatment of Cholera. Nevertheless, I know a gentleman who suffered from Cholera at Archangel, during the last Summer, and who was restored from a state of complete asphyxia by being kept in a warm bath, of high temperature, for an hour and a half. I am sufficiently sensible of the necessity of preventing the patient from using any voluntary exertion, and more especially of strictly preserving the horizontal position during arrested circulation, having witnessed the fatal effects of a departure from this rule in the almost immediate death of the patient; but I am yet inclined to believe, that the prejudice against the use of the warm bath is greater than necessary. The patient might surely be placed in, and removed from it, with such quickness and so little disturbance, as to obviate the objections that have been made to it. It is, however, for the most part in hospital practice only, that the employment of this means of restoring the defective heat of the system could be available since the houses of the poor are seldom provided with the requisite conveniences for preparing a heated bath. Various machines have been invented for introducing heated air into the bed of the patient, but the experience of our hospitals has proved their inefficiency, I believe, without an exception, and their use has been almost entirely laid aside. Mr. Wood, a gentleman of considerable ingenuity in this place, has contrived an apparatus consisting of several thin bottles of tinned iron, which are adapted to the extremities and other parts of the body in such a manner, that when filled with warm water, and cased in bags of flannel, they have been found to answer in a satisfactory manner the purpose of the external application of heat; but on ordinary occasions, we must content ourselves with heated irons, bricks, bags of sand, flannels, &c."

On large warm injections, and the tobacco-enema, we have spoken in our notice of Dr. Lawrie's pamphlet. Mr. Greenhow appears to take much the same view of the tobacco injection as we have done. Friction is the best application to parts affected with spasms, for which, also, a ligature round the limb is often serviceable. If these means have been productive of benefit, the secretions are probably in some degree restored. Mr. G. now recommends calomel and purgatives.

"Unless a considerable quantity (of the calomel) have been already taken, a full dose, varying from five grains to a scruple (in truth I believe it is a matter of much indifference which) may be given; and to fulfil the fourth indication—to obtain healthy evacuations from the bowels and kidnies—the exhibition of a purgative may soon succeed. Castor oil, I am disposed to believe, will be found the most effective and least irritating medicine of this class, but should it fail, other means ought soon to be resorted to, and injections may be employed to assist their operation. Calomel and jalap, or a purgative infusion with carb. and sulph. of magnesia, may be given at intervals until discharges are obtained from the bowels; these will probably be found feculent and bilious, and, for the present, we may consider our patient safe, especially, if, as usually happens, urine be discharged at the same time."

It is in this as in most other violent diseases, what we do not ourselves superintend is inefficiently performed by nurses. If the medical man has any care for his patient in the stage of collapse, he will hardly quit him till he is either recovered from, or past all earthly hope. When the stage of fever has set in, the farther use of stimulants is most pernicious, "the analogy between this and the various forms of continued or inflammatory fever may be considered as complete," and the treatment must be of a corresponding description. Its leading feature is usually organic inflammation, and the treatment must be conducted on general principles. It is at the commencement of this stage that Mr. G. warmly recommends bleeding, "a powerful remedy--powerful when employed at the auspicious moment—powerless when used at a later or an earlier period."

"When we are fortunate enough to be called to a patient before the pulse fails, still more before the serous evacuations commence, when he is suffering from the symptoms which so frequently occur in the first stage—nausea or vomiting, purging of bilious matter, vertigo, head-ache, probably injected conjunctivæ, pain in the abdomen or at the pit of the stomach, with a quick, sharp, or oppressed pulse, and probably occasional cramps in the legs—a full bleeding will be found of the greatest benefit, not only in relieving the existing symptoms, but in averting the impending horrors of the second stage of the disease; this effect may perhaps yet be produced, although the pulse have become feeble and still more oppressed, but not when imperceptible. In such cases it expands and increases in strength and freedom as the blood flows. If, however, asphyxia, coldness and blueness of the extremities have fairly established themselves, the attempt to obtain blood is vain; thickened and stagnant as it is in the vessels, it cannot be made to flow, and if a few ounces be squeezed from the orifices, it hangs from them in long strings, accumulating like stalactites, without producing any beneficial effects. On the contrary, it fatigues the patient, exposes him to the prejudicial influence of cold, and suspends for a time more efficient means of relief. I must, therefore hold bleeding in these circumstances to be inadmissible, principally because it cannot be accomplished; and the attempt injurious, since it diverts attention from measures of less doubtful utility, because they are really practicable."

From what we have seen of the disease now in London, from the results of examination after death, from what we have read of the practice in the North, from the conclusions derived from reasoning on general principles, and from our experience in India, we do most conscientiously believe, that the remedy which we first proposed and employed for the cholera of Indostan, we mean bloodletting, is after all one of the safest and best, when judiciously timed and applied. Thirty-three cases of the disease are related by Mr. Greenhow; but these we must pass over. We may give the results of his observations on the subject of prognosis, that is, on the chances possessed by different individuals when attacked by the disease.

"The chances of recovery in cases of Cholera must depend upon the stage of the disease; the duration of the attack before the employment of judicious remedies; its severity; and upon the age, constitution, and previous state of health of the patient.

It has been seen that when the patient comes under treatment in the early stage of the disease, before the pulse and animal heat fail, his recovery may be calculated upon with much certainty. The abstraction of blood if indicated, a single dose of Calomel and Opium, succeeded by Castor Oil, will frequently restore him to health in a few hours.

When the more formidable symptoms of the second stage have set in with great severity, the chances of recovery are, in all cases, very precarious; and if the constitution be enfeebled by previous disease, or old age, the case may be considered as nearly hopeless. In childhood, youth, and the vigour of life, an active and diligent use of remedies will often be attended with complete success; and it is in these cases that we are stimulated in our exertions by the satisfaction of witnessing their utility. The warmth of the body is, in the first place, restored; the pulse becomes perceptible, it encreases in strength and volume, and the natural colour of the skin gradually returns; the secretions become re-established, and the patient is brought into a state of safety; for, with vigilance, the succeeding symptoms may, with much certainty, be obviated. All this, however, does not go on uninterruptedly. The patient has many uneasy sensations during his recovery from the stage of asphyxia; he becomes restless, complains of pain at the stomach, with occasional nausea, or even vomiting. Still there is restored action of the heart and arteries, the functions of life are going forward, and we may fairly anticipate that, under judicious direction, they will lead to the entire restoration of the patient. But when the restorative efforts are opposed by previous organic disease, general feebleness of constitution, or the worn-out energies of age, we must not calculate too confidently upon any imperfect re-action that may be induced. Heat and pulse may return, the former perfectly, the latter in degree only; the spasms and watery discharges may cease; something approaching to natural excretions may even take place, and yet the patient will not unfrequently sink; not from violent reaction, or the development of local inflammation, but from want of energy in the vital powers to carry forward the attempts at restoration which seem so happily commenced.

The danger of the case is by no means dependant upon the quantity of matter discharged from the stomach and bowels. In some of the worst cases, this is not very considerable, and in some of the most successful, it has been very great. Neither is cramp or spasm a certain criterion. Many fatal cases have occurred, wherein it was nearly entirely absent, or soon ceased. The great danger appears to arise from imperfect or suspended circulation. Let this be restored fully, and the rest is within our control. On this circumstance mainly, then, will rest our judgment as to the probable result of our efforts, though all the concomitant circumstances of the case must be taken into the account."

We have arrived at a question of vital importance to the community—the cause or causes of cholera. There must be two sets of causes, the predisposing and the exciting. But a limited number of any community are attacked, though that number will vary, in the gross or in the detail, with the varying circumstances of the mass, or of a portion of the population; in other words, with the general or partial action of the predisposing causes. The predisposing causes may be natural or acquired, or, as Dr. Lawrie words it, there may be susceptibility and acquired predisposition. In our notice of that gentleman's work, we stated our belief that both were in operation, and Mr. Greenhow is of our opinion. On susceptibility, then, we need say no more, than that it is a property of the human being beyond our ken—something implanted in and mixed up with its organic construction, which does not admit of mechanical or chemical analysis, which can only be known from its effects, and cannot be predicted from any certain data, before those effects are manifest. But *acquired* predisposition is more easily recognized. It is to be found in whatever debilitates generally, and affects the organs involved in cholera particularly. The needy, filthy, ragged, starving, crowded population of the worst parts of Newcastle, and Gateshead, and Glasgow: the inhabitants of the lanes of Rotherhithe, St. Giles's, Chelsea, St. Mary-la-bonne: these it is who have hitherto suffered in this country, these it is who display, in a language too plain to be mistaken, what acquired predisposition consists in. One of the first cases of cholera that we saw in the cholera hospital was a miserable prostitute, an inhabitant of the purlieus of Kent-street, who, according to the statement of herself and of those who knew her, had been famishing with her paramour for a fortnight. The little that a precarious trade brought in, had probably passed to the till of a gin shop. The very first case that occurred in Chelsea, which case we saw with Mr. Gaskill, was in Augusta Court, a miserable alley by the side of the river. It was a girl who was one of five that inhabited one room; the father was nearly blind, the mother bed-ridden, the whole had had nothing to subsist on for a very long period but two shillings per week from the parish, with what little assistance they could procure from relations nearly as destitute as themselves. They scarcely knew the taste of animal food, but had long subsisted on potatoes and what they called coffee. The child attacked had just recovered from small-pox, following scarlet fever. She had gone before her house to play at shuttlecock, in one of those biting days with the wind at North-East, of which we have had so long and curious a continuance in the metropolis; she returned home, complaining of cold and pain in the epigastrium, and at 2, a. m., next morning was attacked with cholera. She not only had not, but could not have had any communication with the parts of the town where the epidemic had just broken out, nor on the strictest inquiry could we find that even any of her family or relations had left the neighbourhood. But this by the way. The place where these poor wretches lived, or rather starved, is a palace compared with many that we have visited in search of the pestilence.

"Of the powerful influence of fear, in particular in predisposing to an attack of Cholera, some remarkable instances have taken place, particularly in the nurse who died at the Sunderland Infirmary, and the Rev. Mr. Scott, of that town. That the same affection of the mind, combined with grief for the loss of a husband, wife, or child, and probably assisted by other predisposing causes, more especially poverty and its concomitants, *has induced the attack in successive members of the same family, admits, I think, of every thing short of absolute proof; for the succession of cases has, for the most part, been more rapid than could be accounted for on any principle of contagion, if, indeed, such an agent exists.* The testimony of Mr. Fife, of Gateshead, a gentleman of much intelligence, whose experience of the disease since its appearance in that town, has been very extensive, entirely confirms this opinion. 'Cholera,' says he, 'is certainly not communicable, for though it frequently attacks several persons in one family, it is either simultaneously, or in such quick succession, as to preclude the idea of their having received it from each other;' and Mr. Brady, of the same place, observes, that 'no principle of contagion could account for such a sudden spread of the disease.' We can only conclude then, that powerful predisposing causes, common to all, were in active operation at the time the efficient cause was called into action.

It has been generally remarked that wherever cholera has hitherto prevailed, it has principally attacked the broken-down in constitution, the dissolute, the abject poor, those devoid of proper bodily comforts, whether in lodging, clothing, or diet, those enfeebled by age, and the inhabitants of low, dirty, crowded, and ill-ventilated situations; and, with few exceptions, such has been the case in Newcastle. These, then, must be considered as the general predisposing causes of an acquired physical nature; we may add to them exposure to great fatigue, damp, cold, or dietetic excess. A diligent enquiry would, I am satisfied, enable us to discover instances of all these circumstances having led, more or less, directly to the attack; but of the effect of the latter cause in particular—dietetic excess—the extraordinary irruption of the disease at Gateshead, in the midst of the Christmas feastings, offers a most remarkable example. It is true that Mr. Fife, of that place, has been led to doubt the effect of habits of intoxication in producing predisposition to cholera, as will be seen in his excellent communication on the subject, wherein he observes that 'from the great proportion of orderly, sober persons and children of all ages, among the patients, I cannot consider drunkenness a powerful predisposing cause, though, for the sake of morality, it is well to favour the opinion.' But, after all, this only proves that such habits are not the only predisposing causes. Innumerable instances might be brought forward wherein the attack supervened either during the continuance of, or immediately subsequent to, excessive indulgence in ardent spirits. Such was the case in two of the earliest instances that occurred in Newcastle, those of Eddy and Mills, and others have come under my own observation. Nor will it admit of a question that their habitual use greatly diminishes the healthy tone of the stomach and bowels, and induces an irritable condition of their mucous lining."

Fear then is a cause, and poverty is a cause, and common sense would whisper, nay, she would thunder in men's ears, if they were not closed against all such artillery, that the removal of those causes would be necessary and proper. Clothe the poor, if you can, and feed them, and give them ventilation, and above all cease to terrify them. No, no, we are mistaken! Fright them to the very top of the bent—din in their ears contagion, contagion, contagion—shun them as you would perdition—drive the rich to their palaces, treble barred, and to their country seats surrounded with cordons of fat menials, armed constables and gamekeepers—cry shame and denounce execration on all who would inspire confidence, calm terror, and make the bad seem better rather than worse—do all this and then deplore the evil, and weep over the misery, and appeal to heaven to avert its judgment. Do all this and call yourself a philosopher and a philanthropist, and damn in this world and the next those who are not like you.

The efficient, that is exciting cause of cholera, or rather the question of its nature, next presents itself. We cannot avoid concluding that there is no one exclusive cause, which makes the disease break out in the predisposed. What that epidemic influence may be which makes cholera prevail, we do not profess to know, nor do we know the causes of any epidemic. They have baffled all inquiries hitherto, and the epidemic cholera is not likely to prove an exception to the general law deduced from the mystic character of its predecessors. But the mind of man is ever ready to catch at aught that will sever a difficulty baffling its solution; hence the vague theories of the ancients, hence the bold generalization of the moderns. When the causes of cholera baffled investigation, the doctrine of contagion was raised, and as it always proves acceptable to governments it was taken up and supported by them, because it afforded excuse for raising armies, and strengthening the executive, and forming boards and bestowing places. It was also a favourite with book-worms, because the evidence supporting it has more of an exact and mathematic character, than the record of circumstances not obviously connected or apparently contradictory. Thus, A. has left a place where cholera was, and comes to B. where cholera is not. Neither A. nor B. perhaps catch it, but B.'s cousin, or some one in the street, or in the town, or in the province is affected, and so contagion is proved, and the question is settled. Some persons to be sure were sceptical of many of the most unanswerable facts, and could explain others without resorting to the doctrines of importation and contagion. But this signified nothing; the cholera was to come and did come by contagion, and all that the practitioners of England had to do, was to watch its progress. Fortunately they have done more: they have looked and thought for themselves.

"The assumed capability of cholera being conveyed by shipping from one country to another, on which our system of quarantine is founded, very naturally gave rise to the suspicion, when it first appeared in the port of Sunderland, of its having been imported from some place on the Continent, where it was known to prevail; and several stories were in circulation descriptive of the manner in which it had thus been introduced. I shall not here repeat any of these tales, suffice it to say that none of them have been in any degree authenticated. That the ships which were blamed for having committed the mischief, were found to have been from uninfected ports, their bills of health clean, and their crews healthy; in point of fact they were fairly acquitted of the charge; and I believe the conviction is now almost universally entertained by the inhabitants of Sunderland, medical and non-medical, that the disease did not reach that place from any foreign source whatever.—It may be further stated that the first case of cholera which took place in this part of the country, was at a considerable distance from Sunderland, having been at a small village called Team, about two miles south-west of Newcastle. This case occurred to Dr. Alexander, of Newcastle, on the 4th of August, 1831. The details are given in the Appendix, No. 1.; other cases occurred at Newcastle simultaneously, if not before the regular appearance of the disease at Sunderland; although want of experience of its true characteristics, and unwillingness to believe in the fact, induced medical gentlemen to endeavour to prove that these were not cases of the new disease; yet subsequent observation has sufficiently proved their identity, and, I believe, it is now generally admitted. Such were the cases of Oswald Reay, which occurred in October, of William Armstrong in the beginning, and of Robert Jordan towards the end of November. On the 7th December the next case occurred, that of Maria Mills, with which commenced the official reports of the Board of Health of this place. The strictest enquiries respecting the origin of these cases have failed to obtain the slightest evidence of their having arisen from any infected source, and seem to prove, in the most satisfactory manner, that, however the disease may have since extended itself, its commencement in the country was spontaneous, upon whatever causes it may have depended."

If, as most believe, the disease sprung up in Asia, and has spread to us, taking fourteen years to march from the Ganges to the Thames, there must be some general cause at work, laughing to utter scorn all boards and quarantines. Had it depended on contagion chiefly, if not solely, as plague may be supposed to do, then the quarantine code might have done for cholera what it has for plague, might have kept the disease from many a city and country which it has desolated. But with cholera it has been otherwise, and the most violent contagionist deplores the inefficiency of his cordons and guarda-costas. What the cause may be we have said that we do not pretend to determine; ignorance is better than false knowledge. It seems that atmospheric irregularities are frequently remarked during its prevalence and progress. The Winter in this town, has been a strange one, the last month's weather remarkable. It appears that in the Autumn preceding the eruption of the cholera at Sunderland, an unusual quantity of sheep had suffered from the rot. The meteorological journal of Mr. Losh demonstrates the unseasonableness and variations of the temperature, &c. Mr. L. observes—

"I have not had much opportunity to observe the birds and insects. But fewer birds of passage have shown themselves, and the wild fruits have remained longer upon the trees than is usual. Flies of all kinds, particularly the common horse fly, have been very frequent; and, in digging and planting, I think I have met with more (and in a more perfect state) torpid insects, such as moths, bumble-bees, &c. than I ever observed before."

Mr. Greenhow concludes that the seasons have been peculiar, and their influence over animal and vegetable life peculiar also. To be sure this will not explain all, but the observation of facts can never be productive of mischief, provided an undue weight is not attached to them. We fear we know little or nothing of the nature of that epidemic influence, which has brought cholera to our shores, or raised it in the land.

"That the atmospheric condition, be it what it may, on which depends the efficient cause of Cholera, has been gradually forming itself in the course of the Summer, is rendered yet more probable, when we review the character of the diseases which have principally prevailed, in the neighbourhood, during that period. They have consisted, in great part, of diarrhœa, common Cholera, dysentery, and a form of continued fever, often commencing with these several forms of gastric and intestinal disease. It has been a general remark, amongst medical men, that the ordinary complaints of the season all tended to resolve themselves into the prevalent febrile affection. Throughout the epidemic, a marked determination has been observed to the mucous membrane of the intestines, showing the irritable condition of that tissue, and in many cases discharges of blood, sometimes to an extraordinary extent, have taken place. It is worthy of remark that this form of fever has been most prevalent among, if not entirely confined to, the more elevated parts of the town, and to the families of a different class of the community, from those in whom Cholera has taken place; that while the dwellings of the superior grades were visited, very generally, with continued fever, the lower and poorer districts enjoyed an unusual degree of good health; and that since Cholera became prevalent, the former type of disease has nearly disappeared, except when it occurs as the sequel, or third stage, of its more formidable successor. These are circumstances well worthy of consideration, and naturally suggest many interesting reflections. Supposing, as seems probable, that considerable analogy exists between the efficient causes of the two forms of disease, we are led to inquire whether the difference is in kind, or in degree only? and why, if they differ in degree only, should the less intense degree affect the better and more comfortable classes, leaving the poorer untouched; and *vice versâ?*

Whatever may have been the cause of this singular distinction, it has not depended upon a mere accident; the line has been far too distinctly drawn to admit of such a supposition, and it must evidently have arisen out of some well-defined principle, though it has hitherto eluded our powers of discrimination. I cannot hope to elucidate the mystery, but would again revert to the fact that, throughout the season, the mucous membrane of the alimentary tube has been especially susceptible of disordered function. The several forms of diarrhœa, dysentery, and common Cholera occurred in greater frequency and severity than in ordinary seasons, and every now and then a case occurred, marked with such unwonted characters, as to lead to a suspicion, at least, of its being the true 'Cholera,' to which the attention of Europe has been of late years so earnestly directed; nor has this been the case in this district only; it is well known that in various parts of England and Scotland similar cases have occurred, which have generally ended in death. Surely there is some general cause for all this. It is impossible not to connect it in the mind, however vaguely, with the morbid cloud which has settled upon our shores; a cloud which, however baneful its effects when resting upon a particular locality, seems to satisfy itself with a determinate number of victims, and then to pass forward a stage in its uninterrupted career. Sunderland has already, after a visitation of a few weeks, emerged from its influence; and, at Newcastle, its work is probably nearly completed; but, in its onward journey, all must expect to add their portion to the accumulating number of its victims."

We need not tell our London brethren that such has also been the case, in a great measure, with us. Bowel-complaints and cholera, to a remarkable and very unusual amount, has been observed during the entire year. We never saw so much of it among the higher classes, nor have we hitherto seen it so severe. It is well known that many have died, and that with alarming rapidity, from the complaint. The cholera constitution seemed forming itself here, as in the North, and the bowel-complaints and cholera of the Summer, Autumn, and Winter of last year may be said, with us, to bear the same relation to the epidemic of this Spring, as the premonitory diarrhœa does to an actual seizure of an individual patient with the malady. Our author examines the postulates of the contagionists in the following order of their affirmation.

1. "That Cholera was introduced into the port of Sunderland by means of ships from some infected place in the north of Europe.

2. That its course could afterwards be frequently traced from one individual to another.

3. That persons employed in the necessary offices about the dead, in placing them in their coffins, and in attending their funerals, have frequently been immediately afterwards affected with the disease.

4. That the disease frequently attacks several members of the same family.

5. That it has frequently been introduced into other places by persons from infected situations.

The answers to some of these assertions have been partially anticipated by what has been already stated. But to shew the uncertain data upon which the supposed introduction of the disease by importation is founded, I shall introduce an extract from a letter from a medical gentleman at Sunderland, who is an advocate for contagion, together with its refutation from his own pen. 'First, then,' says Mr. Green, 'with regard to the origin of Cholera in England (say Sunderland, for it certainly made its first appearance in this place), I shall explain to you two or three facts on this head, and then you may draw your own conclusions. In the month of August last (I believe on the 14th day), a ship from a suspected port, and which had undergone quarantine, passed the Sunderland Roads; two strong and healthy pilots went on board thereof, expecting she was coming into our port, which, however, she did not. The men, in a very short time, descended into their coble to return to the shore. One of them, whose name was Henry (and whose case you may probably have heard something of before this time), was taken ill of Cholera in the boat, before he had time to return to the shore, with every symptom of the disease which has lately been denominated the Asiatic or Spasmodic Cholera, and died in a few hours. The other man also was very soon taken ill in a similar manner, and had a very narrow escape with his life. These, I believe, were the first cases which occurred in this neighbourhood of the disease which has of late produced such consternation throughout the Empire.' What follows is, nevertheless, worthy of especial notice. 'It is, however, only fair to state, that a fatal case of Cholera occurred about a mile from this town a few days previous to the cases above mentioned, attended by a medical man of some information, who informs me that his case bore great analogy to, if it was not identical with, the late epidemic which prevailed here, and is at present prevailing in Newcastle. And one or two other cases, of a very suspicious nature, fell under my own immediate notice, which, however, did not prove fatal, and which, I concluded, were rather aggravated examples of the ordinary Cholera of this country. Subsequent to the date of the two pilots taking ill, a few scattered cases occurred, with an interval of a week or ten days betwixt each, till four cases happened in one day, and then it broke out in full force. I have been informed that there was no case of sickness on board the vessel alluded to above when the pilots went on board, and therefore they could not receive any infection. This argument, I am of opinion, will not hold good. Granted, there was no case of Cholera on board of the ship at the time, if I may be allowed to judge from facts which have fallen under my immediate observation, I am led to believe that the presence of Cholera on board the vessel at the time, was not essentially necessary for the communication of the disease to healthy persons.' I readily acknowledge my obligation to Mr. Green for his communication, and would pay the utmost respect to his opinions, but it cannot be denied, I think, that he is sufficiently credulous as regards the all-pervading power of contagion. At his own request, however, I must exonerate him from the responsibility of the above *facts*. A few days after the former, I was favored with a second letter, of which the following is a passage. It will shew, clearly enough, how much credit is due to the tale of the pilots :—' Since my last communication to you, a further investigation has taken place relative to the case of one of the pilots (Henry), and I find the widow and friends of the deceased tell two stories about it, totally at variance with each other, one of which corresponds with what I stated to you in my former letter, the other is that he got the infection a fortnight before, on board a vessel some distance from this place.' From such evidence as this it is clear that no conclusion can be drawn in favour of the importation of the disease into Sunderland."

Such is the rotten evidence for importation. But supposing one of the stories true, and like the Kilkenny cats they eat each other, how account for "the fatal case of cholera which occurred, about a mile from Sunderland, a few days previous to the cases of the pilots?" In the neighbourhood of Newcastle, too, a similar case had already taken place. And then the ship came from a *suspected port*. Pray what port was that ? Our readers may start when we tell them, that all the ports of Holland were *suspected* ports—of that Holland and those Dutchmen, who now bar us out with forty days' quarantine! The next is a long quotation, but it is a good one.

"The second argument in favour of contagion—that its course could be frequently traced from one individual to another, has also been partly anticipated. It is impossible to examine into all the alleged facts of this description. In many cases I am satisfied, from ample experience, that the persons from whom information is sought will not unfrequently make their information agree with what they suppose to be the wishes of the enquirer—and very naturally so, without imputing to them intentional deceit, when they have no very certain information to communicate. To prove satisfactorily that the disease has actually been communicated from one person to another, it appears necessary to show two things: 1. That communication with the sick has really taken place; and 2. That no other causes were in operation adequate to the production of the disease without the supposition of contagion. Now, that some persons who have had communication with the sick have become ill cannot be denied—to prove the contrary were it practicable, would be too much, it would be to prove that there is *increased safety* instead of *danger* in such communication. But the true question is, does such communication *always* or *more frequently* induce disease, or does the disease *never* take place without it ? To tell us that two nurses died of Cholera, in the hospital at Sunderland, is nothing. How many escaped ? and might not the same women have died from the same cause, if they had never been in the hospitals at all ? Were they free from predisposing causes, especially *fear* or *inebriety*, or from the general exciting cause, the epidemic state of the atmosphere ? for even the advocates of contagion generally believe that the disease is *epidemic* as well as contagious. But what has occurred at the four hospitals in Newcastle and Gateshead ? They have been well supplied with nurses ; they have each had a resident medical man, whose whole time has been spent in the chambers of the patients, directing and assisting in all that was done for their relief; and they have been visited by myriads of medical gentlemen, both residents, and visitors from a distance, none of whom have shrunk from the closest and most frequent contact with the sick ; but in no instance has the natural consequence of contagion ensued; neither medical person nor nurse, nor any individual employed about any of these hospitals has become ill. If contact with the sick will produce illness out of the hospitals, why has it not the same effect within their walls also ? If contagion pervades the bedding and dresses of Cholera patients out of doors, what charm is there about an hospital to prevent the same from being the case in its wards also? But in no case have those employed in removing and washing such articles become ill in consequence. These are the accredited experimental tests by which the contagious nature of Cholera has been put to the proof, and what is the result ? Why with *one*, or at most *two*

exceptions, they have all told against it. But in cases of alleged contagion it is necessary to prove that no other causes were in operation adequate to the production of the disease, without the supposition of contagion. In no instance have I been able to discover that such was the case; however sure it was asserted to be, that the patient had been near to, or in frequent communication with one previously ill, it was equally certain, that they were exposed to the generally prevailing epidemic cause, and that one or more of the predisposing causes were also in existence. This sort of negative evidence may possibly be thought conclusive; it is, perhaps, as much as the opponents of contagion could be expected to adduce against it; but by a rare accident I am enabled to do more. I am enabled to bring forward an insulated case, occurring in a solitary village, which has neither been preceded nor succeeded by other cases, which amounts to the most positive proof. The case to which I refer is that of Margaret Walker. She had been exposed to no source of contagion; but probably an original organic predisposition, and previous delicacy of health, assisted by the depressing influence of grief occasioned by the intelligence of the sudden death of her sister, were the powerful predisposing causes which enabled the efficient cause diffused in the atmosphere (though in too mild a form to affect others in the same locality) to produce its specific effect, and the result was an attack of cholera of the most malignant kind, of which she died in a few hours. Her husband and nine children resided with her in a single apartment; her funeral was well attended; the usual honours of eating and drinking were not omitted, but no one became ill in consequence. Why? because there was not a sufficient concurrence of predisposing and efficient causes to produce disease; but if contagion had been the efficient cause, could this have been the case?—in so malignant an instance it must surely have been in ample force; but the event proved otherwise. There is yet another argument to be deduced from this poor woman's case. Her sister died at a distance of eight miles from her, and she had no communication with her during her illness; if, however, it had happened otherwise—if she had been taken ill an hour after leaving the *death-bed* of her sister, instead of an hour after *hearing* only of her death, what would the advocates of contagion have inferred from such a fortuitous occurrence? Would they not, and with some appearance of reason, have declared that she manifestly received the disease from her dying or dead sister? It cannot be doubted that such would have been their argument, nor would it have been easy to deny its plausibility. In reference to the question of contagion, then, I must consider Margaret Walker's case of the utmost value, as proving — first, the inconclusiveness of the facts brought forward in support of that doctrine, and—secondly, as affording positive evidence against it."

The third argument for the contagion of cholera is the alleged frequency of persons being attacked, who were engaged in assisting at funerals. For this argument to be worth much, we must know how many such seizures there have been in a town, if they are well authenticated, and so forth. For a man who was at a funeral, or engaged in one, to be himself attacked, is no ways wonderful during an epidemic. Persons have moral depression, particularly great at such a period—are exposed to cold—commit excesses — are crowded in the chamber of the dead—in short, exposed to all the ordinary predisposing and exciting causes of the distemper. We have always been struck with one extraordinary inconsistency in the arguments of the contagionists. Not one of them dares to avow that the contagion is of a virulent character, like that of plague; he knows that if he did he would probably find the dimensions of his ears very sensibly increased. Yet many, if not most of the instances adduced by them, are proofs of a contagion of a most violent description. Thus a person enters a ship from a suspected port, cholera not being in the vessel: he descends into his boat, and dies in a twinkling—another merely meets a person who has been where the cholera was, and *his* fate is sealed—a third removes a piece of furniture, and is slain—and so on. Yet in practice and in the gross we find none of this frightful contagiousness. Hospitals are erected, and medical men visit them, and nurses live in them, and clothes go in and out, and furniture travels backwards and forwards, and not a case of contagion can be got for love or money. 'Tis strange. Can there be such things as lies?

"The fourth argument in favour of contagion—'That the disease frequently attacks several members of the same family'—refers to a fact of which there is sufficient proof. But the question naturally arises—under what circumstances did this plurality of cases occur? Did the patients become ill at the same time, or at intervals of sufficient length to admit of a reasonable belief that contagion, communicated from one individual, had time to produce its effect on another? With very few exceptions, I have found that the attack has been either actually simultaneous, or with so short an interval, as to forbid the idea of the intervention of contagion. In the cases of a man and his wife, who died at Walker, the man was seized at 3, and the woman at 7 o'clock on the same morning; their child was attacked in the course of the same day, and also died. In cases which I saw with Mr. Knagg's, of Gateshead, although the most severe symptoms occurred in the daughter, subsequent to their having shown themselves in the father, I found that both had been affected with diarrhœa at the same time, from whence we must conclude that the same cause was brought to operate on both at the same period. It would be easy to multiply such instances. The testimony of Mr. T. K. Fife, of Gateshead, already referred to, appears conclusive. He observes, 'Cholera is certainly not communicable, for although it frequently attacks several persons in one family, it is either simultaneously, or in such quick succession, as to preclude the idea of their having received it from each other; at any rate the progress is much more rapid than that of any other disease propagated by contagion. Among the first 53 cases I attended since the 25th ult., 32 were members of different families; and although few efficient means of prevention were employed, the disease, so far as I know, has not spread in any of them.'"

The last point adverted to is the introduction or importation of the disease. On this we have plenty of slippery evidence abroad, in favour of contagion; at home, where we can use our own eyes, facts go the other way.

"But although many instances are vaguely spoken of, I have not found it possible to verify any in a satisfactory manner. There is some equivocation or doubt in the nature of the evidence, or, at about the same time, other cases occur in the neighbourhood which cannot be traced either to communication with the suspected case or with any other imputed source of contagion.* We have seen how imperfect was the evidence on which the introduction of the disease into the port of Sunderland, by means of ships from abroad, was attempted to be established. At Newcastle not even a suspicion of the kind has arisen. And though at Gateshead the first patient was said to have been lately in that part of Newcastle where the disease prevailed, it yet had no connexion with the fearful number of cases which occurred simultaneously, ten days afterwards, in every part of the town; in nearly fifty different points, cases occurred almost at the same instant. 'On the 25th, about one o'clock,' says Mr. Brady, 'we were assailed by a

* Since this was written three cases have taken place within the walls of the prison, a building constructed upon the most approved principles, and in which the prisoners have been completely insulated, the strictest discipline having been observed, and all communication from without carefully guarded against. For a memorandum of these cases I am indebted to my friend, Mr. Fife, of this town, to whose care the charge of the health of the inmates of the prison is committed.—See Appendix, No. 5.

third and fourth example of the disease, and before the next morning at ten o'clock very considerable numbers had fallen sacrifices to its pestilential ravages.

'Within a space of twelve hours it spread itself over a diameter of two miles, and appeared to pay but very little distinction to altitude of situation, for the higher parts of the town were laid under its stroke in an equal degree, or nearly so, with the lower. Pipewellgate, Hillgate, the banks above Pipewellgate, Oakwellgate, the lanes leading from it, Jackson's Chare, Nun's Lane, Wreckinton, Gateshead Low Fell, Low Team, situations as different in their external characters as can well be conceived, were all indiscriminately exposed to its fury, and I do not think the cases were one whit milder in the more elevated than in the lower parts of the town,' and he afterwards adds, 'That it is virulently epidemic, a glaring proof has been afforded to my mind by the way in which we have been here visited by the disorder in question'—'no principles of contagion could account for such a sudden spread of the disease;' and, in point of fact, such has been the case in every other place where the disease has shown itself. Attempts are made to trace the first case from some suspected source; proofs of it are assumed upon the most uncertain evidence, but the further progress of the disease baffles all calculation, and the contagionist is obliged to seek shelter in the admission that it is both *epidemic* and *contagious*."

Here we must quit the subject of contagion, and pass to that of medical police and prevention. Mr. Greenhow shews the actual and comparative mortality at many places, the erratic course of the disease, its sudden out-breakings when not expected, its general tendency to follow the course of the rivers. We all know the triumphant piece of reasoning founded on this fact. Rivers said the book-men are the great channels of human intercourse; the disease chiefly travelled on their banks; therefore it was chiefly propagated by human intercourse; therefore it was disseminated by contagion. But are rivers the great channels in this country? and are there no other causes near the banks of rivers for the prevalence of cholera. It is by those banks that we find the filthiest lanes, the most abject population, the worst air; it is by rivers we chiefly have malaria, and by rivers we chiefly have cholera. It has gone up the Tyne and down it, but the other "great channels" have hitherto proved no channels for it.

Mr. Greenhow recommends better constructed villages, or at least residences for the poor. It appears that it is intended to destroy the entire village of Newburn, with a view to its re-construction on better principles. Had this been done before, how many lives might have been saved! Will wisdom be learnt in other places?

Hospitals should be erected in airy situations, yet as near the centre of infected districts as possible. A litter carried by means of poles on men's shoulders is the best conveyance, the patient being laid horizontal and perfectly passive. When placed in the litter he should be enveloped in an ample supply of warm blankets, and the heat should be increased about his person by the application of Mr. Wood's vessels of warm water.

"In confirmation of the importance of attending to this subject, I may adduce the opinion of Mr. Glenton who has had the principal charge of the patients admitted into the hospital in Sandgate. It is given in a note to Mr. Fife, and refers to the case of the boy removed from the prison, who afterwards died in that hospital. 'If Mr. Fife has any power to prevent future cases from being removed in the *car*, Mr. Glenton will feel obliged by his doing so. The *conveyance is deadly*, and the boy himself, on reaching the hospital, said, *it had destroyed him*.'"

If comforts can be procured at home Mr. G. strongly discountenances removal. If the stage of collapse has set in, if the weather is cold, or the hospital distant, the danger from removal is considerable.

"On the whole, it is well worthy the attention of Boards of Health, and Committees appointed to visit the houses of the poor, to aim rather at supplying the proper comforts and nursing at home, than to remove the sick to hospitals. If they are enabled to accomplish this with any degree of perfection, they will certainly contribute in a much greater degree to the preservation of life, than by the best hospital arrangements that can be devised. It is an undoubted fact, that the relative mortality in hospitals has been greater than in private houses, and I apprehend it distinctly arises from the disturbance, fatigue, and exposure attendant on the removal of patients, and the consequent delay in the use of remedies."

"The cases admitted into our hospitals, perhaps, have not been sufficiently numerous to warrant us in drawing any general conclusions from them, and it is extremely difficult to obtain accurate information from other sources. Nevertheless I am disposed to believe that the chances of recovery at the different periods of life, as shewn in the following tables, may be considered as consistent with general experience. There were admitted into the four hospitals—

19 cases under 10 years of age, of whom 9 died, or 47⅓ per cent.

79 cases from 10 to 50 years of age, of whom 35 died, or 44⅓ per cent.

20 cases above 50 years of age, of whom 16 died, or 80 per cent.

SANDGATE HOSPITAL.

| | | MALES. | | | FEMALES. | | |
|---|---|---|---|---|---|---|---|
| | | Under 10. | Under 50. | Above 50. | Under 10. | Under 50. | Above 50. |
| Died ............. | 23 | 1 | 4 | 4 | — | 10 | 4 |
| Recovered ........ | 32 | 4 | 7 | 1 | 3 | 15 | 2 |
| Total ............ | 55 | 5 | 11 | 5 | 3 | 25 | 6 |

CASTLE HOSPITAL.

| | | MALES. | | | FEMALES. | | |
|---|---|---|---|---|---|---|---|
| | | Under 10. | Under 50. | Above 50. | Under 10. | Under 50. | Above 50. |
| Died.............. | 8 | — | 3 | 1 | — | 3 | 1 |
| Recovered ........ | 4 | — | 2 | 0 | — | 2 | 0 |
| Total ............ | 12 | — | 5 | 1 | — | 5 | 1 |

ST. JOHN'S AND ST. ANDREW'S HOSPITAL.

| | | MALES. | | | FEMALES. | | |
|---|---|---|---|---|---|---|---|
| | | Under 10. | Under 50. | Above 50. | Under 10. | Under 50. | Above 50. |
| Died.............. | 8 | — | 4 | 1 | — | 3 | — |
| Recoved .......... | 7 | — | 4 | — | — | 3 | — |
| Total ............ | 15 | — | 8 | 1 | — | 6 | — |

GATESHEAD HOSPITAL.

| | | MALES. | | | FEMALES. | | |
|---|---|---|---|---|---|---|---|
| | | Under 10. | Under 50. | Above 50. | Under 10. | Under 50. | Above 50. |
| Died.............. | 21 | 6 | 7 | 1 | 2 | 1 | 4 |
| Recovered ........ | 15 | 2 | 4 | — | 1 | 7 | 1 |
| Total ........... | 36 | 8 | 11 | 1 | 3 | 8 | 5 |

The relative chances of recovery in patients admitted into hospitals, compared with those treated at home, may be estimated, I believe, pretty accurately, from the following facts :—

Of the entire number admitted into hospital, amounting to 118, 60 died, or $50\frac{5}{6}$ per cent.

Of the entire number of cases reported in the towns of Newcastle and Gateshead, up to this day, February 9th, amounting to 1330, 437 died, or $32\frac{9}{10}$ per cent.

It is important that the greater mortality in the hospitals should be attributed to the right causes. There can be no doubt that, as regards the proper comforts, nursing, and medical superintendance, the hospitals, for the most part, have the advantage over the private dwellings of the poor. It appears unquestionable, therefore, that to the delay in the use of remedies, and the unavoidable disturbance and fatigue attendant on the removal of patients, must we attribute the less favourable results of hospital treatment. This ought to excite serious consideration. If the averages be correct, of the 118 patients received into the hospitals, instead of 60, 39 only would have been lost, had their cases been attended to at home."

It appears that in the villages the want of medical assistance was severely felt, many patients dying, without even receiving a professional visit. Many interesting facts are communicated in an Appendix to this interesting and really instructive volume. We are sorry that our want of space compels us to refrain from doing more than compliment Mr. Greenhow on the zeal, activity and candour that he has evinced.

X.

PRACTICAL OBSERVATIONS ON THE MALIGNANT CHOLERA, AS THAT DISEASE IS NOW EXHIBITING ITSELF IN SCOTLAND. By D. M. MOIR, Surgeon. Octavo, stitched, pp. 71. Edinburgh, 1832.

THE Edinburgh folks are stark mad on contagion; they out-Herod Herod in their belief of its omnipotence, and fool, liar, idiot, are profusely showered by its press on all who entertain a contrary opinion. They seem determined to be not only twice as religious, but twice as contagious as other people; they will have *two* fasts, and they will have contagion of double strength. Our readers are aware that Musselburgh has suffered most severely. We find by the report from the Central Board, of the 27th Feb. of the present year, that in this place there had occurred 438 cases, and 193 deaths; whereas, at Hetton, there are reported 476 cases, and only 96 deaths. Now it seems that Mr. Moir, the author of the present pamphlet, is secretary to the Musselburgh Board of Health, to which he dedicates his production. We shall soon see what a stiff contagionist he proves; in fact, he is the hottest champion we have ever been sufficiently unwary to assault. Mr. M. has got the absurd, and now universally laughed-at article in the Quarterly at his fingers' ends. Whether this formed his creed or confirmed it we are not told, and cannot say; neither can we determine whether he was made secretary to the Board of Musselburgh, because he was so stanch on the orthodox side; or, not having yet collected facts enough, whether that appointment itself contributed to strengthen his contagious doctrines, by supplying cart loads of the peculiar evidence which boards appear to have so keen a nose for. Mr. Moir is the author of an Ancient History of Medicine; we shall soon see what sort of stuff a Modern History might be made of. Lawyers know that, to bolster a bad cause, there is nothing to be compared to a stout swearer; there must be no squeamishness—no stickling. To bolster a bad theory, we equally need a profound credulity on one side, as profound a scepticism on the other. The following extract will shew what we mean.

"Whatever Cholera may have been in India, surely the mind must be strangely constituted which can shut itself against the fact of *its being virulently contagious, at least in this climate.* That it ever shews itself sporadically, I am very much inclined to doubt; for in the instances which have been adduced to support that supposition, *the disease has uniformly stopped with the person attacked; whereas, in the Malignant Cholera, no sooner does a case shew itself in any particular street or district, than, if entire isolation be not immediately effected, a second and a third follow, and at length the whole neighbourhood is subjected to its influence. Not a single case has occurred, where communication with the infected could not be readily traced out.* While, on the contrary, where intercourse has been completely cut off, the progress of the disease has been, to a certainty, arrested. What more is wanted than the corroboration of this fact—or call it, in the present state of our experience, assertion, if you will—that the disease is one communicable by personal contact? Wherever Malignant Cholera appears, ample opportunities will be afforded of substantiating its truth."

To be sure some people may stare at this, after reading the paper of Dr. Ogden, and our notice of the works of Dr. Lawrie and Mr. Greenhow, or, better still, those works themselves; but let them stare! We have long ceased to wonder at any thing, save common sense and common candour.

"Indeed the consideration of the subject reduces itself ultimately to the simple question, 'Does Cholera Asphyxia propagate itself by some contagious quality, or does it not?' If it does, how is a sporadic case to be accounted for?"

Exactly so. How does Mr. M. account for the sporadic cases described at Sunderland and Newcastle? We should like to know.

"If it does not, why are the inhabitants of one village, hour after hour, falling victims to it, while another, perhaps not a mile distant, remains perfectly uninfected? How does it pass from house to house along a street, yet stop at the termination of it, where a family have shut themselves up within their garden walls? Were it an epidemic arising from atmospherical causes alone, no such phenomena would, or could, present themselves."

There's the rub. *Does* it stop when a family shut themselves within their garden walls? We should be happy to hear of such instances, and, having heard them, we should then like to ask the questions, how does it stop when families *don't* shut themselves up? how did it stop at the gates of the affluent in Newcastle, Sunderland, Gateshead, &c. and only enter the miserable hovels of the poor? how does it avoid affecting two villages in close communication with one another? how does it stop in families after cutting off one member? how is it confined, as at Hillhead, to a circle of eight houses? how does it stop at doctors' doors and nurses' parlours, sparing even Mr. Moir himself? how does it do all this, and ten times more, in the face of the contagionists? Indeed we cannot tell.

"No stronger corroboration of this position could be any where looked for, than the circumstances connected with the third case which appeared at Musselburgh, and which happened to fall under my charge. This was a girl, whose mother kept a lodging-house, and whom I found in a state of complete collapse on the morning of Thursday, the 19th January—the day after the first appearance of the pestilence. *She died on that afternoon, between five*

*and six, and was buried by moonlight the same evening, in order that the fomes of the contagion might be more effectually counteracted, by having the body removed, and the house properly washed and fumigated.* Notwithstanding every precaution, however, the mother, during the night of Saturday, was also similarly seized, and fell a victim on the following noon. Her sister, who had walked from Leith on the same morning, to condole with her in her family distress, *was immediately affected on entering the house ;* but her symptoms being overlooked in the misery around her, medical assistance was not called in, *until, on the return of the nieces from the interment, their aunt was discovered dead on the floor of the dwelling.* Her husband, Baxter, a man of intemperate habits, *came out to enquire into her fate ; and, immediately on his return home to Leith, was seized with the distemper, and died.* By the vigilance of the Board of Health there, the contagion, so far as this individual was concerned, was, by active measures, successfully checked, and Cholera thus, for a while, held in abeyance."

Was there ever such a fanfaronade, in the way of philosophical evidence and induction ? The third case in Musselburgh was our author's first, but we are not told how that patient caught the cholera. Mr. M. has thus committed the extraordinary blunder, of making his man of straw without a foot to stand on. But to proceed. The Musselburgh contagionists immediately begin to fumigate, and bury by moonlight (for contagionists dread the day), and to wash and to scrub. Well, is the disease put out ? The deuce-a-bit. The mother dies in two days afterwards, and we suppose she is buried by moonlight also. The sister is immediately seized *on entering the house*, and is found dead after the burial, we suppose a very short period indeed. The husband comes out to inquire, goes home, and dies also. But in Leith, where the last person lived, the Board are more fortunate in their fumigations than the Musselburgh folks were, and the disease appears no more. Evidence can be carried no farther. No ! we are mistaken, it might. Mr. Moir, in his next chain of instances, should crown all by an instance of the cholera going to a yet more distant relative per post, in the black edging of the funeral letter. Seriously, cannot men unriddle this dark history ? Can they not see, in the moonlight burial, and the fumigations, and the grave looks of the Board, and the averted footsteps of the neighbours, and the preachings and the fastings which are afflicting Scotland, can they not see, we say, in this every cause for terror in its intensity, and for the consequences of that terror, excessive predisposition amongst the unfortunate relatives? Will any man make us believe, from what we have *seen* now and *read*, that the contagion of cholera is so virulent as to kill at this fearful rate, to strike a woman down on her entering an apartment (one fumigated, too, by the worshipful Board), and kill her ere the termination of a hurried funeral rite!

"Taking another illustration from the first six in the schedule of District First, I find a boy, W. B., aged seven, to whom I was called *at six on the morning of the following day, Friday, the the 20th. At eleven at night, a man lodging in the same house was also attacked ;* and on the following the father of the boy. Within a week after, his youngest child, aged only three years, became also violently affected. *Eight or ten other cases almost immediately shewed themselves, within a compass of thirty yards from this house ; and this at a time when whole districts of the parish remained totally uninfected.* Indeed, the whole history of the disease among us, from first to last, is nothing more than a consecutive series of examples, strikingly illustrative of the personal communicability of the contagion. That it can, besides, be conveyed by inanimate substances, is also very probable ; but the testimony on this subject is more difficult of attainment, while it is less capable of proof."

One patient we see, is attacked within 18 hours of another, and twelve or thirteen cases occur within a compass of thirty yards, whole districts remaining totally unaffected. Did the whole districts shut themselves up in their gardens, or did the persons beyond the pale of the thirty yards, vote the sufferers from cholera nuisances, and draw a cordon around them? Why was the disease limited to so small a sphere—was there no communication save within it—were Mr. Moir and others washed and fumigated on leaving their patients within the thirty yards, to visit friends, patients, &c. without them? These are queries that present themselves to our weak fancies, which can hardly be answered by the doctrines of contagion, but may on the grounds which we and others have advocated.

"It is quite a mistake to suppose that the disease is either so erratic or whimsical, in its wanderings from district to district, as some would lead us to suppose; or that it will suddenly disappear, after having wreaked a desultory violence. This idea is founded on the erroneous supposition of its non-contagious nature, and a number of apparent illustrations have been brought forward in its support. The fact is, that cholera will nowhere abate, or pass away, while a victim susceptible of its influence remains, provided such be exposed so its contagion; and no doctrine more dangerous or destructive to the safety of society can be promulgated. Until all are made aware of the risk to which they are exposed—adopt every means of prevention—and keep beyond the pale of contagion, the disease will propagate itself—and re-appear—and again re-appear with renewed force. The necessity is therefore urgent for continuing every preventive restriction and regulation in full force; to isolate every case that can be so managed; and to render the concentration of the pestilential fomes less virulent, by feeding and clothing the poor, as well as attending to the purification and cleanliness of their dwellings. The fear has been suggested, that public panic may become so universal regarding the infectious nature of the disease, that it may be found impossible to procure the necessary attendants for the sick; but hire every where—or the nobler incentive of moral obligation in a Christian land—will ever be able to more than compass this end. But even were it to act in a degree sufficient to be somewhat felt—better, perhaps, that it should be so, than that thousands should be running themselves, without caution or warning, into the vortex of death—and not only becoming uselessly self-sacrificers, but the very vehicle for scattering the seeds of destruction every where around them."

If it be a mistake to suppose the cholera erratic, it is one into which abler men than we have fallen. But look at it now. It is in Edinburgh, Glasgow, many parts around: it came a little south of Sunderland, to Durham, but there it vanished: and now it is in London. This looks a little erratic. The only other evidence on the subject of contagion is to be found in the Appendix, for all works on cholera must have that. We have the old story of the Mauritius, and the Topaze frigate, and Moreau de Jonnés trumped up, as if men could now be employed better in looking carefully and without prejudice for themselves than in hunting for contagion in foreign chronicles and compilations.

"As far as my personal observation extends, truth is entirely enlisted on the same side. From the appearance of the disease in Mid-Lothian, to the present moment, there has not been a single break in the chain of contagion—not a single instance in which its source was not to be traced. Since the illustrations I have noted were marked down, a third more remarkable sorites has occurred in the family of Mr. M——, a gentleman of extensive connexions in the mercantile world, and whose loss has been very generally regretted. Underneath his counting-room lived an aged dependent, Jeanie Findlay, who was taken ill on the morning of Saturday, 28th January, and died on the evening of the same day. *No suspicion of contagion attached to her death, although thus sudden, being in the eighty-fourth year of her age;* but on the Tuesday Mr. M.'s third son was seized with cholera,—himself, and eldest son on the Wednesday,—and Mrs. M. on the Thursday. All died from the immediate attack of the disease except the first, who lingered on till the seventh day; and it was found that they had severally been sitting by the bedside of their old servant. As a still farther proof, however, that the contagion was thus engendered, two neighbours, Mr. and Mrs. H., who had gone in to see the old woman, also sickened and died; and on Monday, 30th, her son, R. Stewart, a shoemaker, having carried from her house to his own some articles of furniture, was shortly after taken ill. When called in to him, I found him in a state of collapse, from which he could not be resuscitated; and he died on the following morning. Within 24 hours of his death, his wife was also taken ill, and was sinking into the cold stage when seen. This, however, she was brought through; but being feeble and somewhat advanced in years, she fell a victim to the consecutive fever, five days afterwards. By unremitted exertions as to fumigations, the contagion has been checked, in so far as regards the other branches of the family."

Mr. Moir always leaves one link out in his contagious chain. How was the first old woman contaminated? We are told that no *suspicion* of contagion attached to her case. If there did not, the case gives Mr. M. a death blow at once; if there did, he should at least let us have it; if neither supposition is correct, but the English is at fault, we cannot help it. For the rest of the instances we refer to what we have already said, to what Mr. Lawrie and Mr. Greenhow say, and to our readers' common sense. But we have not quite done.

There can be no doubt at all that the mortality at Musselburgh has been fearful. We also think there can be little doubt that the secretary of the Board is a contagionist of the first water, and that probably he and the quorum do not greatly differ. Here then is a contagious board, acting up to their professions and belief, burying by moonlight, fumigating, promulgating notions of contagion without hindrance, inspiring panic to their heart's content. And what do we see? Victims are dying around them. The blessed seeds of contagion have been planted in the church-yard of Musselburgh, and they have ripened, not like the dragon's teeth, into warriors, but into tombs. Happy Scotland, your physicians are taking all human means to dispatch you to the heaven, which your ministers are using all holy measures to prepare you for.

---

### XI.

### ON MALIGNANT CHOLERA IN ENGLAND. By MR. DODD, OF HOUGHTON-LE-SPRING.

[Lancet, March 3, 1832.]

THE progress of truth is now more marked than the progress of cholera, in the northern parts of the isle. It will form a subject of astonishment to future medical historians that the nearer the epidemic approached to the "Modern Athens"—the "intellectual city"—the more did common sense desert the human mind, and superstitious terror usurp the place of reason! The cordons of the Russian Bear were scarcely less imperative and tyrannical than the police videttes of auld Reekie's magistrates to keep out the cholera of Sunderland and Haddington! As for contagion—Pooh! The Musselburgh Secretary demonstrated that cholera could not possibly be propagated in any other way than from man to man. Do our Caledonian confrères jump at the conclusion that, because a certain *national malady* is highly contagious, the cholera must also be so? We have no other way of solving the problem respecting the prevalence of contagious doctrines in the Land of Cakes. It was probably fortunate for science and for humanity, that the epidemic broke out on the banks of the THAMES, where a host of intelligent practitioners were instantly on the watch to mark its track. It was only this day (March 2,) that one of the most erudite and influential advocates of contagion declared his conviction, after a keen examination of the cases in Southwark, that the doctrine which he so strenuously upheld, must be given up, or greatly remodelled. This declaration does him infinite credit, and it is greatly to be wished that medical men would abandon preconceived notions, and permit their judgments to be directed by the evidence of their senses. But we must now introduce an extract from a paper of Mr. Dodd's, recently published in our contemporary, the Lancet.

"I feel some hesitation in advancing any thing in disproof of the now very prevalent opinion of the contagious nature of cholera; perhaps future contact with it, and a more dispassionate inspection of its workings and progress, should it ever again become a visitant amongst us, may elucidate this undecided question. Few of the continental writers, and amongst the rest Broussais, believe it to be a contagious disorder; and though the terms infection and contagion are, by the bulk of the profession, used synonymously, yet I am here inclined to give to each a distinctive signification. Cholera, in my opinion, is an infectious disease, inasmuch as it is received from a column of pestilent matter arising in, or borne to, certain districts, where it is attracted or detained by local causes hitherto unknown and undefined. I say undefined, as there has been so little regularity as to the kind of place where it has raged, contrasting it with those naturally productive of other endemic complaints; and cholera has been equally fatal amidst the isolated hamlets on the banks of the Tyne and Wear, as amongst the idle, depraved, and dissolute inhabitants of the crowded alleys of the sea-port town. As to contagion, using it in the sense of its derivative, I believe it to be totally inapplicable to cholera. Staggering instances have been adduced, where I have thought it difficult to withhold my conviction of its transmission by human intercommunication; but I have, in every case, been able to satisfy my own mind, that the patient had received the virus from an infected atmosphere, subjected to its operation at some distant point, whilst labouring under one or more of the predisposing causes which I am about to define.

The *disposition* to accept the choleric poison may be either *natural* and *hereditary*, or it may be *accidental*. That there is a natural predisposition in some families to be infected with this disease, I have very little doubt; for I have seen many examples of the father, mother, and successively their children, succumbing to the overwhelming malignancy of its course, whilst other families living in their immediate vicinity, and having unchecked intercourse with them and other infected persons, have escaped. The individuals so attacked have always been of a leucophlegmatic appearance; indeed, I have never yet seen one of a healthy, sanguine, or florid complexion, die of cholera, and as I know many who have been attended by other practitioners, the same remark holds good.

The *accidental* predisposing causes are—grief, watching, fasting, want of cleanliness, innutricious and irregular diet, the depression which succeeds the excitement from drinking ardent spirits, utero-gestation and parturition; in short, whatever produces diminished energy of the nervous system, and lessens vascular action to the surfaces. Almost every case which I have been called on to treat or visit in conjunction with others, I have been able to trace to the intermediate operation of some of these causes, without appealing to the theory of contagion, when it is but assumed to exist.

The mode of an attack of cholera is not always the same, though its specific action tends, in every case, to depress the powers of life; but at what point or condition this resistive force is sufficient to oppose itself to the acceptation of the virus, I am unprepared to define. Those of a sanguine temperament, I assume, possess more of this resistive power than those of a pale complexion and languid circulation—*i. e.* putting aside the consideration of accidental causes which may affect them; but should any one, although naturally constituted and defended against the insidious approach of the disease, fall, through the operation of any of the accidental causes before enumerated, below *par*, whilst breathing a tainted atmosphere, he will, in all probability, be infected with cholera; but, on the other hand, as long as his body retains sufficient energy—as long as the tone of his system rises superior to the depressive agency of cholera, he may breathe this pestilential matter, without danger of becoming one of its many victims.

In districts where cholera has raged as an epidemic, and I may mention particularly the High Downs, a colliery village in this neighbourhood, scarcely one individual has escaped being affected with some of its milder premonitory symptoms; this I attribute to the varying constitutions of the persons forming this little community, and the *actual* capabilities of resistance which each possessed; some succumbing at once to the more violent form of the complaint, whilst others remained uninfected, till some accidental cause, very often the effects of inebriety, rendered them capable of being so. We are all alike subject to the influence of this pestilential vapour, but the labourers of this district are placed under disadvantages from which the operatives of other classes are to a certain degree exempt; and toiling as they do, from early youth, breathing vitiated air at an excited temperature, are more liable to an attack of cholera than he who lives in well-ventilated rooms, and enjoys better means of restoring the waste, which the comparatively less exhausting nature of his employments has produced."

External heat and friction were employed first, and, as soon as possible, a vein was opened and blood drawn, The next remedy administered was croton oil, which abated the spasms and restored the secretions. More recently calomel was employed, and with decided advantage. When vomiting and purging were excessive, the mustard emetic proved very beneficial. Mr. Dodd concludes with the observation, that VENESECTION is our sheet anchor in this disease. The paper is very creditable to the author.

---

XII.

NITRIC ACID IN TOOTHACHE.

*Pembroke Dock,* 1*st Dec.* 1831.

SIR,—In the Periscopic department of your highly-estimable Journal for October last, there is an extract from a paper published by Dr. Ryan, in a cotemporary periodical, on the utility of nitric acid in toothache, which the Doctor states was found out by a Mr. Myers. Now although he may, by accident, as it is observed, have fallen upon the remedy, yet I trust you will do me the justice to insert this letter in your forthcoming Number, as laying claim to its prior discovery. During the Autumn of 1820 (eight years before Mr. Myers knew of its employment), I suffered greatly from toothache; and after adopting every known means of relief, short of extraction, I was induced to try the application of nitric acid, upon the principle of its destroying the sensibility of the exposed nerve; it had the desired effect in that instance. The tooth is still in its place, and has never ached since. From that period, I have been in the habit of using it pretty freely, and with considerable success.

Knowing that "est natura hominum novitatis avida," I should, ere now, have published the efficacy of this plan, but have waited hitherto for the test of experience, which alone, like Ariadne's silken clue, can lead us out of the Cretan labyrinth of theory and speculation. I have found the acid most serviceable when the affected tooth has been situated in the lower jaw, from the greater facility of its introduction into the cavity. When in the upper, the difficulty is greater and the success less certain. A tumbler of cold water should always be at hand, into which the finger is to be instantly dipped, and applied to any part of the tongue, cheek, or gum touched by the acid.

With the other remarks of Dr. R. I bear testimony and fully coincide; and have the honor to be,

Sir,

Your obedient Servant,

W. THOMAS, M.R.C.S.

XIII.

Parts II. and III. of the Principles and Practice of Obstetric Medicine, &c. By David D. Davis, M.D. &c.

In the second fasciculus Dr. Davis gets through the anatomy of the pelvis, and takes up the subject of diseases of the same, as distortion from rickets, from mollities ossium, &c. In the 3d chapter of this part, the Doctor examines, with his usual ability, the structure and diseases of the external genitals. From this chapter we shall extract the narrative of a curious case of pruritus occasioned, as Dr. D. supposes, by exuberance of hair.

"A case of severe pruritus of the genitals occurred in the author's practice about two years ago, in the instance of a young married lady, who was the mother of one child. She was not the subject of ascarides, nor of any other intestinal worms. The uterus was healthy in structure and functions. There was no eruptive affection either of the vulva or vagina. The contents of the bladder were voided without pain or difficulty. It became a matter of suspicion, that the mons veneris might be the seat of a pedicular affection. This was not the case. But the opportunity of examination conceded for ascertaining that point, led to the discovery that the entire pudendum, together with its neighbouring surfaces even to distant portions of the common integument, the posterior perinæum, the whole of the hypogastrium, and even parts of the abdomen intermediate between the umbilicus and scrobiculus cordis inclusive, were covered with a most extraordinary quantity of hair. The subject of this history having been more or less harassed with the pruritus, which she was now suffering, since the commencement of her fourteenth year, when she menstruated for the first time; it did not seem quite foreign to the probabilities of the case, to conjecture that this superabundance of the hairy covering of the pubes might, by promoting the evolution and retention of an undue phlogosis in the uterine system, act as an indirect cause of the distressing symptom. This presumption was, at all events, acted upon, and the patient was furnished with an unguent consisting of two parts of quick-lime, and six of strongly-scented pomatum, accompanied with directions to apply it by friction to the mons veneris and contiguous integumental surfaces twice a day. These operations were more than once obliged to be suspended in consequence of the irritation, amounting to a slight fretting and inflammation of the cutis, which they occasioned. In about two months, a very large proportion of the hairy covering was removed; and as the process of depilation advanced, the author had the pleasure of observing, that the patient experienced a sensible abatement of her pruritus, whilst about the middle of the third month, subsequently to the commencement of the practice, the symptom had entirely ceased. To leave no room for misapprehension or miscalculation of effects, the author deems it his duty to add that, inasmuch as this treatment was adopted as a matter of experiment, to which he attached no very eager expectation of success, it was throughout accompanied by the exhibition of alterative and aperient medicines, and of lotions and injections of various kinds to the vulva, vagina, and uterus. The subject of the case has never experienced a return of her complaint."

The plica of Poland is briefly alluded to, as also a peculiar conformation observed about the pubes of Egyptian women.

In the third part or fasciculus, Dr. Davis continues the diseases of the external organs, and the whole is very well handled by this industrious and experienced Professor. The plates accompanying these parts are very creditable. We shall have more and better opportunities, hereafter, of noticing the parts as they come forth. Meantime, we may venture safely to recommend the work to all our obstetric readers, and especially to the aspirant for professional reputation in that department of medical science.

XIV.

LETTERS UPON CHOLERA MORBUS, WITH OBSERVATIONS UPON CONTAGION, QUARANTINE, AND DISINFECTING, FUMIGATIONS. By WILLIAM FERGUSSON, M.D. Inspector General of Hospitals. 8vo. Stitched, pp. 39, London, 1832.

Those who know Dr. Fergusson need not be informed by us that the excellence of his heart and the goodness of his understanding have alike prompted him to come forward on the present occasion, and to lend his powerful aid in dispelling the panic so injurious to this country in every point of view. It is indeed to be wished that every honest man should do his best to promote so useful, nay we may say so holy a purpose, as *unfrighting* the isle from the paroxysm of terror into which it has been thrown. The mischiefs from excess of confidence are not to be compared for a moment with those from excess of fear, and though every term of obloquy and reproach has been heaped upon the heads of those who are conscientiously engaged in endeavouring to dispel injurious alarm, we do repeat our exhortation for all members of our profession, who aspire to be good citizens and philosophic men, to labour in private and in public in that honourable cause. Supposing the doctrine of contagion to be true, and taking it in the sense and to the extent to which the contagionists carry it we firmly believe it is not, yet supposing for the sake of argument that it is so, we do conceive and contend that infinitely more evil than advantage would accrue from dinning it into the public ear. Some men never seem so happy, as when they are penning articles to prove the disease most malignant and terrific, most infectious and pestilential; they dwell with an obvious satisfaction on the lists of the dying and the dead; rub their hands and chuckle on its irruption into some new village, town, or district; and wear a mortified and peevish air if you hint that the daily numbers of the victims have decreased. No crime is so heinous in the eyes of these gentlemen, as a disposition to look at the fair side of the picture, no offence so monstrous as an attempt to make the public easy. It is quackery, and humbug, and folly, and what not; in short these Mr. Surlys are determined to be miserable themselves, and, so far as their power goes, to render the remainder of the world so. If indeed any good could come from fear one might feel disposed to excuse them, but as they admit themselves that panic is injurious, we can conceive no earthly reason why they should wilfully and wantonly persist in provoking it. But it is hard to kick against the pricks, and still more difficult to persuade people out of violent and obstinate party feeling. Nothing will make them cease to cry wolf, save a consciousness that the world is not inclined to listen to them.

In reference to the contagiousness of the disease, Dr. Fergusson alludes to the fact now notorious, of the perfect impunity with which crowds of medical men from all nations visited the north of England and hunted for cases of cholera. He also alludes to an absurdity of the contagionists, which no evidence will ever make them willing to abandon, we allude to their assertion that cholera always follows the great channels of human intercourse. Thus it has been for a month (we write on the 4th of March) in the Borough, and we ourselves saw it some two weeks ago, at the very foot of the Southwark and of Blackfriar's bridge, yet it has not hitherto contrived to cross the London Bridge, or effect a lodgement in the city, whilst it is at Lambeth, Chelsea, Newington Butts, &c. But the contagionists will probably say that London and Blackfriar's bridges, cannot properly be considered as examples of "the great channels of human intercourse." They need not be afraid to utter such opinion; it is as good as most, and better than some of the logic they do venture on.

"So far from always following the stream and line of population as has been attempted to be shown, it has often run directly counter to both, seldom or never desolating the large cities of Europe, like the plague and other true contagions, but rather wasting its fury upon encampments of troops, as in the east, or the villages and hamlets of thickly-peopled rural districts.

That it could have been descried on no other than the above line must be self-evident, but to say that it has followed it in the manner that a contagious disease ought to have done, in our own country for instance, is at variance with the fact. From Sunderland and Newcastle to the south, the ways were open, the stream of population dense and continuous, the conveyances innumerable, the communications uninterrupted and constant. Towards the thinly-peopled north how different the aspect,—townships rare, the country often high, cold, and dreary, in many parts of the line without inhabitants or the dwellings of man for many miles together, yet does the disease suddenly alight at Haddington, a hundred miles off, without having touched the towns of Berwick, Dunbar, or any of the intermediate places. It is said to have been carried there by vagrant paupers from Sunderland. Can this be true? Could any such with the disease upon them in any shape, have encountered such a winter journey without leaving traces of it in their course?* or, if they carried it in their clothing, the winds of the hills must have disinfected these *fomites* long before their arrival. No contagionist, however unscrupulous and enthusiastic, nor quarantine authority however vigilant, can pretend to say how the disease has been introduced at the different points of Sunderland, Haddington, and Kirkintilloch,—no more than he can tell why it has appeared at Doncaster, Portsmouth, and an infinity of other places without spreading. Even now, it lingers at the gates of the great open cities of Edinburgh and Glasgow, as if like a malarious disease, (which I by no means say that it is) it better found its food in the hamlet and the tent, in fact, amongst the inhabitants of ground tenements, than in paved towns and stone buildings. We must go farther and acknowledge, that for many months past our atmosphere has been tainted with the miasm or poison of Cholera Morbus, as manifested by unusual cases of the disease almost everywhere, and that these harbingers of the pestilence only wanted such an ally as the drunken jubilee at Gateshead, or atmospherical conditions and changes of which we know nothing, to give it current and power. That the epidemic current of disease wherever men exist and congregate together, must, in the first instance, resemble the contagious so strongly as to make it impossible to distinguish the one from the other, must be self-evident; and it is only after the touchstone has been applied, and proof of non-communicability been obtained, as at Sunderland, that the impartial observer can be enabled to discern the difference.—Still, however, must he be puzzled with the inexplicable phenomena of this strange pestilence, but if he feel himself at a loss for an argument against contagion, he has only to turn to one of the most recent communications from the Central Board of Health, where he will find that "That the subsidiary force under Col. Adams, which arrived in perfect health *in the neighbourhood* of a village of India infected with Cholera, had seventy cases of the disease the night of its arrival, and twenty deaths the next day," as if the march under a tropical sun, and the encampment upon malarious ground, or beneath a poisoned atmosphere, were all to go for nothing; and that the neighbourhood of an infected village, with which it is not stated that they held communication, had in that instantaneous manner alone, produced the disease. This is surely drawing too largely upon our credulity, and practising upon our fears beyond the mark."

As disinfecting agents, Dr. F. recommends heat, light, water, and air, and they are infinitely better than all the chemical compounds that ever were issued from a laboratory. Can any thing be better than the high, we had almost

* The Cholera in this country would appear always to travel with the pedestrian, and to eschew the stage-coach even as an outside passenger.

said chivalrous, tone of the following passage, and when such are the sentiments which animate the bosoms of all who have come forward as "anti-contagionists," in the proper sense of the term, can any thing be more dastardly, and more impotently absurd, than the attempt to stigmatize such men as bent on injuring society, for the paltry personal advantage of notoriety. So far from its being the interest of medical men, in London, to take a prominent part on this side of the question, it is quite the reverse. By so doing, they array themselves, in some measure, against authority—make public enemies, and too certainly offend many private friends—expose themselves to present derision and abuse—and should circumstances in any way run counter to their hopes or their expectations, are assailed with every engine of vituperation, which malevolence and furious party vengeance can employ. There is every thing to lose and nothing to gain, in a personal point of view, and only a professional zeal and enthusiasm, which cold-blooded calculators cannot comprehend and therefore malign, has tempted men to venture, as they have done, before the public, in order to serve it in a trying moment.

"It may then be asked, have we no protection against this fearful plague? No means of warding it off? Certainly none against its visitation! It will come—it will go; we can neither keep it out, or retain it, if we wished, amongst us. The region of its influence is above us and beyond our control; and we might as well pretend to arrest the influx of the swallows in summer, and the woodcocks in the winter season, by cordons of troops and quarantine regulations, as by such means to stay the influence of an atmospheric poison; but *in our moral courage*, in our improved civilization, in the perfecting of our medical and health police, in the generous charitable spirit of the higher orders, assisting the poorer classes of the community, in the better condition of those classes themselves, compared with the poor of other countries, and in the devoted courage and assistance of the medical profession every where, we shall have the best resources. Trusting to these, it has been found that, in countries far less favoured than ours, *wherever the impending pestilence has only threatened a visitation, there the panic has been terrible, and people have even died of fear;* but when it actually arrived, and they were obliged to look it in the face, *they found, that by putting their trust in what I have just laid down, they were in comparative safety;* that the destitute, the uncleanly, above all, the intemperate and the debauched, were almost its only victims; that the epidemic poison, whatever it might be, had strength to prevail only against those *who had been previously unnerved by fear*, or weakened by debauchery; and that moral courage, generous but temperate living, and regularity of habits in every respect, proved nearly a certain safeguard. They found further, *that quarantine regulations were worse than useless*—that the gigantic military organization of Russia—the rigorous military despotism of Prussia—and the all-searching police of Austria, with their walled towns, and guards and gates, and cordons of troops, were powerless against this unseen pestilence, and that as soon as the quarantine laws were relaxed, and free communication allowed, the disease assumed a milder character, and speedily disappeared.

I say, then, confidently, that Cholera Morbus never will commit ravages in this country, beyond the bounds of the worst purlieus of society, unless it be fostered into infectious, pestilential activity, by the absurd, however well-meant, measures of the conservative boards of health, such as have been just recommended in what has always been esteemed the most influential, best-informed journal of England, I mean the QUARTERLY REVIEW."

We do not believe that the quarantine that actually was established in this country, contributed to protract the establishment of the epidemic among us for a single day; nor that, if their turns are to come, will the cordons and coast-guards of foreign governments achieve more than ours have done. The following facts are most important; they would open the eyes of Bigotry, if

Bigotry's eyes could be opened. They are sealed with a sleep ten million times deeper than Nourjahad's.

"My friend, Doctor Stanford, of the Medical Staff, now settled here, has given me the following valuable information, which my own observation confirms, regarding the agency of panic, in promoting the diffusion of epidemic disease. He happened to be serving with part of the British army, at Cadiz, when an eruption of yellow fever took place there, in the Autumn of 1813, and as usually happens amongst medical men, the first time they have seen that fever, some of them were stanch contagionists, and impressed that belief upon the corps to which they belonged. *In all these the disease was most fatal to great numbers.* The men being half dead with fear, before they were taken ill, speedily became its victims, to the great terror and danger of their surviving comrades; *but in the other regiments, where no alarm had been sounded, the soldiers took the chances of the epidemic with the same steady courage they would have faced the bullets of the enemy, in the lottery of battle;* escaping an attack for the most part altogether, or if seized, recovering from it in a large proportion. From this picture let us take a lesson, in case the impending epidemic should ever come to spread in the populous towns of England, and the cry of contagion be proclaimed in their streets. The very word will spread terror and dismay throughout the people, causing multitudes to be infected, who would otherwise, in all probability, have escaped an attack, and afterwards consign them to death in despair, when they find themselves the marked and fated victims of a new plague. Whatever they see around them, must confirm and aggravate their despair, for desertion and excommunication in all dangerous diseases, too certainly seal the fate of the patient. It will be vain to tell them that hireling attendance has been provided,—the life of the Choleraic depends upon the instant aid—the able-bodied willing aid of affectionate friends, who will devote themselves to the task, and persevere indefatigably to the last. If these be driven from his bed, his last stay is gone, for without their active co-operation, the best prescription of the physician is only so much waste paper. What, let me ask must have been the fate of the patient, and what the consequent panic, if the case of Cholera that occurred in London a month ago, at the Barracks of the Foot Guards, had been proclaimed, and treated as a contagion? The poor fellow was promptly surrounded by his fearless comrades, who with their kind hands recalled and preserved the vital heat on the surface, by persevering in the affectionate duty of rubbing him for many hours; but had the Medical Staff of the regiment been true contagionists, they must, as in duty bound, have commanded, and compelled every one of them to fly the infection. It depended upon them, to have spread around a far wilder and more dangerous contagion than that of Cholera Morbus, or any other disease,—the contagion of fear—and from what occurred at Cadiz, as above related, it is to be hoped our medical men will now see how much they will have it in their power, when Cholera comes, to pronounce, or to withhold sentence of desolation upon a community. The word Contagion will be the word of doom, for then the healthy will fly their homes, and the sick be deserted: but a countenance and bearing, devoid of that groundless fear, will at once command the aid, and inspire the hopes that are powerful to save in the most desperate diseases.

It is stated, in a Scotch newspaper, that two poor travellers, passing from Kirkintilloch to Falkirk, ran the risque of being stoned to death by the populace of the latter place, and were saved from the immolation only by escaping into a house; and in an Irish one, that some shipwrecked sailors incurred a similar danger. Such barbarities must, in the nature of things, be practised every where under a reign of terror, however humane or christianized the people may be—even the fatalism of the Turk would not be proof against it. In Spain they have been enacted in all their horrors (thanks to the quarantine laws) upon the unfortunate victims of yellow fever: and we shall soon see them repeated amongst ourselves, unless the plain truth be promulgated by authority to the people. Let them be told, if such be the pleasure of our rulers (for it is not worth while disputing the point), that Cholera Morbus is a contagion, but of so safe a nature in regard to communicability, that not one in a hundred, or even a thousand, take the disease,—that in this country, besides being a transient passing disease, which according to certain laws and peculiarities of its own, will assuredly take its departure in no long time; it is limited almost always to particular spots and localities—that it is in their own power, while it remains, to correct the infectious atmosphere of these spots, by attention to health police—that they may fearlessly approach their sick friends with impunity, for that the danger resides in the above atmosphere, and not in the person of the patient; and that in all situations they may defy it, for as long as they observe sobriety of life and regularity of habits. Thus will public confidence be restored, and thus be verified the homely adage of, 'honesty, in all human affairs, being ever the best policy;' for the concealment, or perversion of the truth, however much it may be made to serve the purposes of the passing day, can never ultimately promote the ends of good government and true humanity, but must lead, sooner or later, to the exposure of the delusion, or what would be far worse, to the perpetuation of error and prejudice, and grossest abuse of the people, in regard to those interests committed to our charge."

Dr. Fergusson deserves the thanks of all friends to mankind for these Letters.

---

XV.

A LETTER TO THE LONDON BOARD OF HEALTH, OFFERING A NEW, CONCISE, AND INTELLIGIBLE VIEW OF THE LEADING PHENOMENA OF THE PRESENT PESTILENTIAL CHOLERA, BY WHICH A RATIONAL, SIMPLE, AND SUCCESSFUL METHOD OF TREATMENT IS ATTAINED. By THOMAS BROWN, Senior Medical Practitioner, &c. &c. Musselburgh. Octavo, stitched, pp. 40. London, 1832.

IT appears to be admitted on all hands, by contagionists and anti-contagionists, ultras and moderates, journalists blue, green, and yellow, in fact by all men of all parties, that nothing is so conducive to health, nor so powerful in warding off cholera, as a cheerful indeed jocose state of mind. It is on this account that we notice the pamphlet before us; and we venture to indulge the hope, that such of our readers as may honour the present article by their perusal, will find themselves steeled against the pestilence for at least the succeeding twenty-four hours. Mr. Brown is the partner of Mr. Moir, who is secretary to the Musselburgh Board of Health, who wrote a pamphlet to prove the cholera contagious, and who has found a niche in our columns which we trust he will like. Mr. B. addresses his Letter to the London Central Board, a compliment of which we have no doubt the members of that body will feel proud. The mortality at Musselburgh has been so great, that both the profession and the public have been anxious to ascertain what peculiar and local circumstances could have made it so. In our notice of Mr. Moir's production, we have hinted at one not impossible cause of that fatality, the ultra-contagiousness of the Board and the doctors. We shall see that this idea derives fresh strength from Mr. Brown's letter. Mr. B.'s experience differs from that of the Indian practitioners, and he thinks it necessary to let the world and the Board of Health know why.

"I must also be permitted to add, that I consider myself to be bound to do so, from having had an ample opportunity of trying my opinions by the test of experience, *which has confirmed them by the success of our practice*; and I flatter myself it will be ultimately found, that although I do not enter minutely into the subject, still I shall make my opinions sufficiently plain and intelligible, and will have contributed my mite to the public advantage.

Before proceeding further, it may be proper to notice, that Mussleburgh is situated on the river Esk, close by the sea; and the town, as well as the parish of Inveresk, is nearly equally divided *through its whole extent by the river.* The parish contains a population of somewhat above 9100, and the town of Mussleburgh and its immediate connexions about 7500 of this number. The town is open and well aired, and its soil in general dry, and is nearly equal in warmth, and has less rain, than perhaps any parish in Great Britain. Notwithstanding these advantages, our population may be described *as vicious, immoral, and miserable;* a full half being liable to the vice of all others the most destructive to religion and morals,—I mean drunkenness. This state, however, it must be observed, has not been produced by any innate viciousness or folly, but has arisen entirely from the unparalleled continuance of the last war, whereby Mussleburgh, being a favourite station for the army, was inundated with 1500 soldiers, and all their unpleasant concomitants, for the space of twenty-two years. As a proof of the healthy situation of the whole town and parish, I may state from personal experience, during forty-four years, that I have never had a single case of Ague occurring periodically; and before the improvement in agriculture, I have known all agricultural labours at a complete stand, from the farmers' servants being laid up by Ague;—and only when crowded with the military, together with high-priced provisions, and other causes of misery, were liable to Typhus. For these last twelve months, poverty, conjoined with the vice of drinking, and wretched accommodation, has been again operating very extensively; *and we were suffering considerably from Typhus, when this horrible pestilence appeared,* and which has, in the short period of little more than five weeks, carried off nearly 250 of the inhabitants, being about one in every thirty-five of the parish, and nearly equal to twenty months of the usual mortality; and before we finally get rid of this dreadful scourge, our deaths, I have no doubt, will amount to 300, being nearly one in thirty of the whole population."

Thus it seems that Musselburgh is divided by a river—that its population is vicious, immoral, and miserable—that at the time when cholera appeared, it was suffering from typhus—that Mr. Brown is its senior surgeon, and that his partner is Mr. Moir—and last, not least, that its board and its doctors are contagionists to the back-bone. We need not be surprised at the havoc which the cholera has made in this fortunate locality.

"*It is also deserving of particular notice, that there has not been one individual affected with the Cholera in Mussleburgh, who lived in a front house, or was unconnected with closes or alleys.*"

Oh, ho! So none but those connected with closes or alleys were affected even in Musselburgh. Were none but those so situated exposed to contagion? Let Mr. Brown be permitted to answer.

"I must here avail myself of the publication of my friend and partner Mr. Moir, and refer you to it for a complete refutation of the doctrine of the anti-contagionists; I shall, however, observe, that when I have enquired what these gentlemen meant, in answer to the *notorious fact, of every one infecting another, from absolute contact, either in their persons, apartments, or clothes, or with some one or some thing with which they had had connexion,* you really can obtain nothing but the most vague, indefinite, and wild answer, which it is impossible for any one accustomed to the examination of data really to comprehend, or put into the shape of probability. It is as clear as noon-day that it is contagious; but these gentlemen insist that it is also epidemic, without offering any reason, but that it cannot be proved in *every* instance to have been transmitted by contact,—a request deserving of not the very least attention; because whoever attends to the communications existing in the present state of society must be at once satisfied, *that there can be no want of opportunities of contact always being afforded;* and although these gentlemen were accidentally to lay their hands upon one, who will not confess that they have been in contact with the disease, I should be glad to know the value of such, when it is well known to us all, with what difficulty we confess any fault, and especially, at present, the fault of transmitting Cholera; particularly, too, when this charge attaches to those who would have no great delicacy about the matter at any time."

So the disease is not epidemic nor endemic, but always contagious. Yet this same Mr. Brown informs us that it never appeared but in those connected with closes or alleys, whilst he is kind enough also to shew, or rather to admit, for all must know it, that *every one* is exposed more or less to the influence of contagion. To ask Mr. Brown to explain this or to explain himself would be unfair, as it would be nothing less than asking an impossibility. Some charitable persons may think that we have been hard on the Musselburgh Board, and suspect that we have accused them of more folly in their contagiousnesses than they deserve. Let the following extract form our defence.

"The means which the profession have employed here, for preventing the infection spreading in the houses and apartments of those labouring under the disease, are, fumigating, with the means already mentioned, with the addition of the fumes of the nitric and muriatic acids, burning tar, turpentine, vinegar, fumes of tobacco, flashing gunpowder, throwing vinegar and spirits of camphor throughout the apartment, and paying great attention to keeping the bed-clothes and surface of the body wholesome by cleanliness, and sprinkling of vinegar mixed with spirit. When death has ensued, and the family removed, or whether or not that can be obtained, the house is whitewashed with hot lime. *From the steady use of these means, we have, in general, succeeded in checking the farther spread of the pestilence in the immediate dwellings; and have also found them of great benefit in further securing the public safety.* With regard to myself, for the first five or six days of the Cholera raging here, I was quite sensible of its exerting an influence over me, such as headach, sickness, coldness of the surface, and pulse about 96, with a disordered state of the stomach and bowels; but by taking care never to visit any one before swallowing some breakfast, *with half a glass of the very best brandy after it,* again taking *a glass of the very best port or sherry about one o'clock,* with a biscuit, and dining regularly at, or as soon after four as possible, upon a plain but *rather solid diet,* and with *two or three glasses of port wine,* if soup or fish made no part of my diet; but if I ate of several dishes, I preferred the good old Scottish custom *of a dram to the wine,* and found myself always *much lighter* after this last practice. I never used tobacco, although I have recommended it to general use, in any form; but I washed my temples and forehead *with aromatic vinegar,* and spirits of wine and camphor, mixed with a little water, every time I returned home, and also applied the mixture to my clothes, and these, together with *chewing cinnamon,* and keeping a regular state of the bowels, formed the whole of my defence; and in eight days I got the better of my different ailments; my pulse became regular, and I now enjoy better health than before the scourge came amongst us."

So the tar, turpentine, gunpowder, camphor and the cart loads of other rubbish were found of great benefit in securing the public safety! Mark that; the PUBLIC SAFETY. Is it not laughable, or rather abominable, that we should be coolly told that the public safety was secured in a place rendered famous by mortality only. The public safety indeed! The passage should be written thus: "From the steady use of these means, we have, in general succeeded in promoting the farther spread of the pestilence in the immediate dwellings; and have also found them of great benefit in farther securing the public destruction."

We are not a little struck at the different manner in which Mr. Brown proceeded in his own case. The public may imagine that contagionists practise what they preach—that they fumigate themselves so many times a day—sprinkle their clothes with nitric and muriatic acids—put gunpowder in their waistcoat pockets—and burning tar in their inexpressibles. No such thing; these gentlemen take care to apply the spirits to the inside rather than the out. Mr. Brown's mode of treating himself gives a lively and graphic picture of the hygiene of one of this school. Let us see; there was half a glass of the *very best* brandy in the morning to start with—a glass of the *best* port or sherry at one P. M.—two *or three* glasses of port at four P. M.—and, oh luxury, if *several* dishes were eaten of, an additional *dram* to the wine. The singular part of this diary of an invalid consists in the fact, that he was *lighter* after these superlatively good things. However disposed to cavil at mere doctrinal points, we are forced to confess, that when we come to practice we can take no reasonable exception against Mr. Brown. We must only regret that himself, Mr. Moir, and the Board, did not treat their neighbours as themselves, and, instead of arointing the poor with burning tar and gunpowder, that they did not distribute a little of that very best brandy and port which they poured so liberally into their own stomachs. We have great pleasure in drawing the attention of the Londoners to the following caution.

"*No female servant should be allowed to go out of doors, and nobody's servant allowed to come into the house. Bakers and butchers, &c. should be dismissed instantly upon delivering their errand;* in short, high and low should assist and forward the grand object of public safety, *by cheerfully submitting to every subordination which those in authority think necessary in the present emergency.* They should also conduct themselves with moderation and propriety, and show a proper feeling under the present scourge. There should be no general revelling, feasting, and extravagance, the public distress giving a ready vent for all our superfluous enjoyments."

It will naturally become an anxious subject of inquiry, whether the prohibition is to be extended to male servants, and if it is not, why? It will also be important to ascertain whether or not butchers and bakers ought to be permitted to come to our houses at all, for timid persons may imagine that if they stop long enough to give their meat and bread, and errands, and receive fresh orders in return, the mischief is already done, and the cholera is as likely to make its entrance as the mutton.

"I cannot allow this subject to escape from me, without adding one word more, and that is, to declare that no nation deserves, or can thrive without religion and morals being made its foundation. I am afraid the one received its death-blow nearly three years ago, and the low price of whiskey in Scotland will finish what remains. It is imperatively demanded that the landed proprietors, the church, and the public must instantly apply to government for the only remedy—raising spirits to ten or twelve shillings per gallon."

So it seems that religion and cheap whiskey can never go together, not even in the moral atmosphere of Scotland. The minimum of sound presbyterianism, is when whiskey is at ten shillings per gallon; under that, it is no better than episcopalianism, it is even a tinkling cymbal. It would perhaps be unprofitable to enquire whether the best brandy and port, are so unfavourable to heavenly thoughts and aspirations, as whiskey, the spirit of the poor. How it glads a rich man to denounce the vices and infirmities of paupers. The tenant of the club sits him down and dines on the fat of the land, drinks his pint or his bottle of good old wine, and heaves a sigh at the immorality of the wretched artisan, who hurries to drown misery and care in a quartern of gin. The religious world cries out for restrictions on the atrocious wickedness of the lower orders—sends the pestilence upon them as the wrath of the Divinity—and imposes in addition the mischievous cruelty of human power.

We have very little more to say to Mr. Brown. He thinks that the present *only* differs from common cholera arising from heat, as it has acquired the adjuncts of being *unconnected* with warm weather, as being more putridly malignant, and as possessing distinctly a highly contagious property." Very slight differences, indeed. The following is another profound observation.

"I am also satisfied, that the situation of my patients clearly evinced, that if the evacuations had not already gone on to the extent of carrying away all offending matter, they had, at all events, existed to the extent of reducing the patient to a state of *articulo mortis*, so that every further effort in *that way* appeared evidently not only to be useless, but likely to be followed by the most fatal results."

The articulo mortis, in Italics, belongs to Mr. Brown, not to us. The judicious reader will observe the climax, as well as the Latinity of the passage. Mr. Brown admits the injurious influence of fear; indeed we should attach high value to his opinion on this point, as he seems to have had some *personal* experience of the effects of alarm, and, thanks to the judicious measures of himself and his friends, must have had an opportunity of witnessing its effects on a large scale. To dissipate it, he recommends carminative purgatives. We should advise the instant abolition of any board, acting on such notions as the Musselburgh sages appear to have done.

---

XVI.

PARTIAL FRACTURE OF THE LONG BONES IN CHILDREN. By J. HART, M.R.I.A. &c. &c.

AT length the silent city, for the silence of its university is typical of itself, has put forth a medical periodical. Yet the effort appears to have been a great one; and Medicine alone has not yet felt able to shake off the lethargic drowsiness which oppressed her, without the friendly aid of Chemistry. The journal which has just seen the light in Dublin, and of which the first number is now before us, is entitled, the Dublin Journal of Medical and Chemical Science. It consists of 116 pages, 56 of which are devoted to original communications — 17 to bibliographical notices, and the remainder to articles of miscellaneous intelligence. Whether it is to be monthly, quarterly, semennial, we are not officially informed, nor, strange to say, do we find the usual prospectus, which tells how such a publication is wanted, and how lamentably deficient all existing sources of periodical information are acknowledged to be. We hail with pleasure our contemporary from the green isle, though, by the bye, its wrapper is of deep yellow, and need scarcely say that we wish it all success. It is at present too late to give an adequate notice of many of the useful papers it contains, and we must content ourselves with the first, because it is the shortest. It is on the partial fracture of the long bones, which is frequently met with in practice.

"The bones differ very much in the relative proportions of the animal to the earthy part entering into their composition at different periods of life, a circumstance which materially affects both their liability to fracture, and the length of time necessary for the accomplishment of re-union in individuals of different ages.

Thus, in infancy and childhood the animal part of the bones bears a greater proportion to the earthy, whence they possess a greater degree of flexibility. It is owing to this that fractures rarely happen in young children, notwithstanding the many falls to which they are subject, before they have acquired the power of maintaining their equilibrium in their earlier essays in walking.

On the other hand, the bones of persons advanced in life are harder and more brittle, in consequence of the accumulation of an increased proportion of the earthy part, to which is to be attributed the more frequent occurrence of fractures of the long bones, in elderly persons especially.

As the bones of children are less liable to fracture from the cause assigned above, so is the process of their re-union more speedily accomplished, inasmuch as their growth being still in progress, the blood-vessels engaged in the function of their nutrition are in a more vigorous state of action; while in the case of older persons, whose bones contain a less proportion of the animal part, and more of the earthy or saline constituents, re-union proceeds more slowly, because ossification being completed, the nutritious blood-vessels which were actively engaged in that process have fulfilled the task which was allotted to them, after which these vessels undergo a diminution in size, and a corresponding relaxation in their activity.

While the long bones of children are still soft and flexible, they are subject to a kind of injury, the occurrence of which is incompatible with the brittleness of the same organs in adults. This injury is a fracture which extends through a part of the diameter of the bone, the remaining part becoming bent in the manner in which a branch of a tree yields to an attempt to break it while it still retains its sap.

It has fallen to my lot to meet with five cases of this injury within the last three years, one of which occurred in the humerus, two in the radius, and two in the femur, and as this kind of fracture is not particularly described in any of our systematic works on Surgery, nor in any periodical publication to which I could obtain access, I shall briefly notice the particulars of one case, and conclude this Paper with one or two remarks on it.

Tuesday, February 10th, 1831, I was called to see Richard K., a child aged ten months, in consequecce of an accident, of the nature of which his nurse affected ignorance. I found him labouring under the following symptoms: pain caused by handling the left thigh, which presented a marked deformity, having an angular projection forwards about its centre. On comparing this limb with the opposite one, it did not appear shortened, nor was there any turning out of the toes. A straight line from the great trochanter to the outer condyle, measured about a quarter of an inch less, while a line from the great trochanter obliquely across the front of the thigh to the internal condyle of the femur, gave a little better than a quarter of an inch more than similar measurements made on the opposite thigh.

I applied splints and bandages, which were removed on Saturday, the 21st, the eleventh day from the occurrence of the injury, when firm union had taken place. At this time the limb differed from the opposite one in no other respect than in having a slight prominence on the front of the femur, at the place where the fracture had occurred.

January 16th, 1832. I this day saw the child, R. K., eleven months after the accident. There is no perceptible difference between the lower limbs of both sides, with the exception of a slight ridge which can still be felt on the front of the femur at the place of the former injury. He is beginning to walk, and makes equal use of both limbs.

The diagnostic symptoms of this affection are very simple, they are the following: pain and a bent state of the bone injured, without absolute shortening of the limb; on the contrary, it is lengthened on the side to which the ends of the fractured part of the bone project. By attending to these circumstances, it will be always easy to distinguish this injury from ordinary complete fracture.

*Treatment.* The first indication in the treatment is to straighten the bent bone; to effect this, much care and delicacy of manipulation are required, for if it be rudely attempted with a force too great or too suddenly applied, the part of the bone which was merely bent may be broken, and the fracture rendered a complete one, the difficulty of treating which without deformity, will obviously be greater in a child, than in a person who can understand the necessity of submitting to restraint.

The next indication is to prevent the recurrence of the deformity, and to keep the fractured surfaces in contact until they become united by callus, and this is to be fulfilled by the judicious use of splints and bandages, as in ordinary fractures.

I need hardly remark on the shortness of the time (eleven days) in which reunion was completed in the above case: it illustrates the principle laid down in the commencement of this Paper, which refers to the rapidity of the re-union of fractures in children, owing to the more active state of the vessels engaged in the business of ossification at that period of life."

We have seen many instances of such incomplete fracture, and, unless our memory greatly belies us, have related some in a former Number of this Journal, though we have not leisure to search for them at present. However, we have twice seen this accident in the clavicle, once in the tibia, twice in the femur. We would particularly direct attention to a modification of fracture, probably of this description, which occurs in the bones of the fore-arm. We have seen three instances of what we are about to mention. A boy receives a fall, and in endeavouring to save himself, the weight of the body is received upon the outstretched hand. He applies to the surgeon, with the bones of the fore-arm bent either towards the palmar or dorsal side. They are obviously curved, nay, they even form an angular projection, and moderate force is quite ineffectual in restoring them to their proper shape. In each case we put the fore-arm across our knee, used considerable force of the leverage kind, heard the bones give an audible snap, and immediately were able to restore the limb to its natural shape. Under the application of proper splints and compresses, applied as for fracture of the fore-arm, for the usual time, we always succeeded in preventing all deformity and inconvenience. We mention these circumstances to put practitioners on their guard, as we have seen unpleasant mistakes occur.

---

### XVII.

### Mr. Fergusson on the Preparation of Soap Cerate.*

"As considerable difficulties have been encountered in the preparation of soap cerate, according to the formula of the London Pharmacopœia, a few observations on their cause, and the best method of making the preparation, may not be considered useless. The Pharmacopœia directs a pound of semi-vitreous oxide of lead to be boiled in a gallon of vinegar, till it incorporates with it, and eight ounces of soap having been dissolved in the solution, the water is to be evaporated slowly, and the residuum formed into a cerate, with oil and wax.

The properties of soap cerate depending entirely on the salt of lead which it contains, it is impracticable to make a useful preparation according to this formula. I have always found, that upon adding the soap to the solution of subacetate of lead, an immediate decomposition of the materials takes place; the potash of the soap, decomposing the metallic salt, combines with its acid, and forms acetate of potash, while the oil unites with the oxide of lead, forming common lytharge plaster, which rolls about in a heavy mass in the solution of acetate of potash. Should the evaporation now be continued, the residuum, instead of consisting of subacetate of lead, combined mechanically with soap, will contain only lytharge plaster, acetate of potash, and a little protoxide of lead.

By a slight change in the process, a cerate may be made which will contain the metallic salt undecomposed, and in consequence be possessed of the properties expected to be found in the preparation. Instead of adding the soap to the unevaporated solution of subacetate of lead, it should be boiled down slowly, till it is the consistence of treacle, and in that state be added to the other materials, melted together, and suffered to cool till they begin to grow white; the whole must now be stirred constantly while stiffening, like Turner's cerate, or ointment of the nitrate of mercury.

The quantity of vinegar necessary varies with the proportion of acetic acid it contains, 50 parts of acetic acid requiring 108 of lytharge to saturate them, about 7.28 ounces of pure acid

* Dublin Journal of Medical and Chemical Science.

will be sufficient to decompose 1 pound of lytharge; of purified pyroligneous acid (specific gravity, 1020,) 24.34 ounces contain the requisite quantity, but if common fruit vinegar (specific gravity 1014) is used, a little more than nine pints will be required; vinegar of this strength containing but one-twentieth of its weight of acetic acid. An inconvenience, however, attends the use of pyroligneous acid, as from the quantity of carbon precipitated during the evaporation, the cerate will be of a dark colour."

---

### XVIII.

### Cases of Acute and Chronic Laryngitis. By M. Cruveilhier.*

We have not hitherto noticed M. Cruveilhier's excellent work so much as its merits demand. Totally differing in plan from the Morbid Anatomy of Baillie, it abounds in cases which may be made available and useful, independently of the plates which the author has subjoined to them. This, however, is in other respects a great disadvantage, as the limits of the work will be extended beyond all moderate bounds. We shall select for a short article some examples of laryngeal inflammation.

M. Cruveilhier divides the diseases of the larynx into those which occur above the glottis, those below it, and those which attack the glottis itself. Of all laryngeal affections, inflammation, as might à priori be conceived, is the most frequent, and, whether acute or chronic it either attacks the cellular tissue external to the mucous membrane, or the mucous membrane itself.

Case 1. *Inflammation of the Cellular Tissue above the Glottis—Œdema of the Glottis.*

Pierre Vrain, æt. 56, an old soldier, and addicted to drinking, was seized on the 26th April, 1829, with lassitude, and next morning complained of sore throat and pains in the limbs, with fever. On the third day the soreness of the throat was great, deglutition and articulation difficult, and the velum palati was seen red and swollen. *(V. S. ad* ℥x. *Garg. Ros. c. Acid Hydrochlor.)* In the evening the breathing was very difficult, sibilous, with threatening of suffocation; the voice only heard at intervals, hoarse, and croupy; the velum more red and swollen, the isthmus appearing almost closed. *(Hirud.* xxx. *gutt.)* After this the patient experienced great relief, and on the fourth morning the soreness of the throat and dyspnœa were gone. Three white patches were observed on the posterior arch, but the redness and swelling had almost disappeared. In the evening all the bad symptoms returned, and delirium supervened. On the fifth day the breathing was very difficult and noisy, the pulse almost imperceptible; at noon he died.

*Sectio Cadaveris.* The two folds of mucous membrane forming the sides of the superior orifice of the larynx, that situated posteriorly at the sides, in the neighbouring part of the pharynx, the base of the tongue, on the anterior surface and superior border of the epiglottis, were all of whitish colour, and infiltrated as it were with pus; the two mucous folds already alluded to, especially the left, were enormously swollen, projecting towards each other, so as to be in approximation every where, excepting for a small space behind, where a small aperture only remained for the passage of air. The glottis, and the portion of the larynx below it were more natural. On cutting through the parts affected the pus was found to be diffused in the cells of the cellular texture, and not collected into any distinct abscess. The mucous membrane itself appeared soaked with pus, and its vessels were only detected here and there. Already several sloughs were completely or incompletely detached, and small blood-vessels injected marked their boundaries in a distinct manner. The tonsils were sound, the anterior surface of the epiglottis was covered with sloughs, and its inferior

---

* Anatomie Pathologique. Cinquieme Livraison.

part tumid and enlarged contributed to contract the opening of the glottis. A representation of the larynx of the patient accompanies the case, and displays in a marked manner the almost complete obstruction of the air-passage, by the infiltration of the pus in the loose cellular tissue external to the mucous membrane.

M. Cruveilhier makes some judicious reflections on the foregoing case, which is more important in its character, and more worthy of study in its details, than a superficial reader or practitioner might possibly suspect. It is important, because it is one of a class not very unfrequent, and equalled in insidiousness only by its fatality; it deserves consideration because it displays a common professional mistake, that of considering a patient as out of danger or in little danger, when, in point of fact, his existence is in peril. We have seen several instances of the same description, and we speak from personal experience and observation. But we shall notice an observation or two of M. Cruveilhier's. He looks upon the affection as one of diffuse inflammation of the cellular texture external to the mucous membrane of the larynx. Of this there can be no question. He considers that there exists a great analogy between it and the angina œdematosa. They are similar in their seat, but not altogether in their nature; for in one the cellular membrane is infiltrated with serum, in the other with pus. But on the surface of the body we constantly witness the effusion of serum preceding that of pus or lymph in the subcutaneous and in the inter-muscular cellular membrane, and it is possible that a similar course may be observed in these anginæ. We do not look on the point as determined, for reasons into which we cannot enter at present. If, says our author, a membrane, which is attacked with inflammation is firmly attached on one side and free on the other, the inflammatory effusions are thrown out on its free surface; but if loosely connected to other parts by the cellular tissue, effusions are apt to take place in the latter, and he might have added, to be diffused in it to a great and a fatal extent. Thus we have seen in examples of fatal cynanche, an effusion of lymph and pus commence in the cellular membrane external to the mucous membrane of the pharynx and larynx, and spread thence between the œsophagus and spine as low as the diaphragm, between the œsophagus and larynx, and between the larynx and its muscles throughout the front of the neck. Those who have bestowed much attention on the affections of the cellular membrane will experience no surprise at this.

With respect to symptoms we can say little more than that they are those of acute cynanche, and that they are often most deceptive. We should say, however, that there is often a degree of anxiety of aspect, and appearance of something wrong, disproportionate to the obvious mischief. M. Cruveilhier insists very properly on careful examination of the throat internally and externally. We should endeavour to obtain a view of the epiglottis if possible, and frequently we may do so by patience and gentleness. Its condition of redness, paleness, or tumefaction is an index of the state of the larynx above the glottis. Dr. Thulier, of Lunoges, has recommended examination of the part by the finger, but, supposing that accurate information were thereby obtained, patients will seldom be found to submit to this manual inspection in this complaint. In chronic diseases of the larynx, M. C. allows, and we agree with him, that this mode of investigation is extremely useful, and we know that it is practicable.

The treatment recommended by M. Cruveilhier is active and judicious:—a copious bleeding in the first instance, leeches, emetics. To this he should have added the inhalation of vapour, and the administration of calomel with active purgatives. But this will not suit all cases, and when the disease attacks the aged or the debilitated, which it too frequently does, the cautious practitioner will hesitate before he employs very violent measures. In such, calomel and opium, with sudorifics, purgatives, leeches and local steaming, must be chiefly relied on.

CASE 2. *Inflammation of the Cellular Membrane of the Larynx below the Glottis—Death from Suffocation.*

A young Swiss was received into the Maison Royale de Santé, under M. Duméril, with pneumonia. He was convalescent from this, when he complained of a violent pain in the region of the larynx, though examination of the fauces, and of the region above and below the os hyoides elicited nothing unnatural. Dyspnœa and great difficulty of articulation soon succeeded, with paroxysms of suffocation in which the voice, respiration, and cough were croupy. He died on the fifth day from the commencement of the symptoms.

*Sectio Cadaveris.* The larynx, examined from behind, presented a projection at the level of the cricoid cartilage, which when exposed was found denuded, perforated, and reduced to a very thin lamina, surrounded with pus which was contained between it and the thickened mucous membrane. There was no trace of perichondrium. The arytenoid cartilages, surrounded with condensed cellular and mucous membrane, were no longer articulated to the cricoid, disorganized as it was. The laryngeal muscles were all infiltrated, but with what we are not informed. The disorganization of the cricoid cartilage extended to its sides, especially to the left.

M. Cruveilhier observes, that the death of the patient from suffocation is readily explained, by the projection, internally as well as externally, of the thickened mucous membrane and perichondrium detached from the cartilage. M. Cruveilhier also believes that the case was essentially one of inflammation of the cellular membrane external to the mucous. In support of the opinion he cites the following case.

*Case.* A young man convalescent from typhus, was suddenly seized with hoarseness of the voice, unattended with difficulty of breathing. Eight days after the invasion of the symptoms he died, without any paroxysms of suffocation, and apparently from obstruction of the air-passages. On examination a collection of matter was found in the substance of the right mucous fold forming the superior aperture of the larynx. The arytenoid cartilage deprived of its perichondrium, was bathed in the pus. M. Cruveilhier also adverts to another case published by M. Bouillaud in the Journal Complementaire, 1825, tome 19.

*Case.* A man, æt. 22, had been in the Maison de Santé for two months, on account of a putrid fever. Eight days after leaving the establishment, he presented himself at the Cochin Hospital in the following condition. There was extreme hoarseness, which had existed for a month, harsh cough with great soreness of throat, and threatening of suffocation on the slightest exertion. Next evening the restlessness was such that the patient could not remain in his bed, the respirations were deep, prolonged and frequent, accompanied with a rough sound, like that of a base-viol, and the patient was in a paroxysm of terror and suffocation, with a pulse scarcely to be felt. The patient was rather better during the night, but the symptoms returned next day. On the fourth day he had a paroxysm of dyspnœa, almost amounting to suffocation, and in the night he died.

*Sectio Cadaveris.* On the posterior part and sides of the larynx was an abscess capable of containing a filbert. The cricoid cartilage was denuded, as though dissected, and the abscess almost surrounded it. The crico-arytenoid muscles were bare, of greenish colour, and thickened ; the nerves could not be traced. The articulations of the arytenoid cartilages with the cricoid were entirely destroyed.

CASE 5. *Chronic Ulceration of the Larynx, and partial destruction of the Epiglottis—Death by Suffocation.*

A man, æt. 40, applied at the Maison Royale de Santé, with all the symptoms of phthisis laryngea ; deglutition was extremely difficult, almost always followed by long and severe cough, sometimes even by threatening of suffocation. On examination M. Cruveilhier discovered thickening of the uvula, velum, and soft palate, with ulceration of the borders and anterior surface of the epiglottis. He had had cough and hoarseness for eight or ten months, but had only ceased to work for fifteen days. He died suddenly.

*Sectio Cadaveris.* The uvula was furred and thickened; the posterior arch of the palate thickened also; the epiglottis ulcerated and festooned in appearance at its circumference; the superior orifice of the larynx and the mucous membrane covering its posterior surface, thickened, ulcerated, and granulated. The whole interior of the larynx presented the same thickening and granulated appearance; the base of the right arytenoid cartilage was denuded, ulcerated, and partially ossified. The lymphatic glands at the sides of the larynx were indurated and infiltrated with tuberculous matter. The lungs were closely adherent to the thoracic parietes, and crowded with miliary tubercles; in the summit of the right lung was a large cavity perfectly cicatrized on its internal surface.

M. Cruveilhier observes, that he has witnessed sufficient cases to enable him to pronounce with confidence on the existence of phthisis laryngea, as a distinct disease. It is much more frequent notwithstanding in combination with tubercular disease in the lungs, in fact, we should say, from what we have seen of cadaveric investigations, that a very large proportion of phthisical patients present small laryngeal, or at all events tracheal, ulcerations previous to death. Here we must conclude these cases, and although there is no fact absolutely new to be found amongst them, no doctrine of novel application to be drawn from them, no peculiar or successful plan of treatment advocated or even hinted at, still we think that their importance is sufficient to merit the attention of practical men. The insidious character and too fatal nature of these affections of the larynx cannot be too forcibly, too clearly, or too often pointed out, for many a man has in some degree lost his professional character by attaching too light importance to diseases of this description.

---

### XIX.

### MIDDLESEX HOSPITAL.

MR. LAIDLAW, formerly house-surgeon at this institution, has related the particulars of several cases which occurred in it during his consulship.* We shall select two or three for a brief notice.

CASE 1. *Rupture of the Kidney.*

"October 11th, 1829. Alexander Elliot, a remarkably fine healthy young man, twenty-one years of age, while running at full speed, came unexpectedly in contact with the iron palisades of one of the squares, and struck his abdomen against them with such force that he was thrown to the ground by the shock; he immediately got up, and not feeling himself, at the moment, much hurt, walked home, a distance not greater than a hundred yards. Soon after he got home, he became very sick, and vomited, but did not consider it of much consequence, till about two hours afterwards, when, having occasion to make water, he found nothing but blood flowed from the bladder; and he then became alarmed, and procuring a coach, had himself conveyed to the hospital.

At the time of his admission, when I first saw him, he was pale, cold, and covered with a clammy perspiration; his countenance was anxious, and he was greatly agitated. Upon a superficial view, he appeared far more alarmed than injured. He complained of pain in the belly, particularly in the right hypochondriac region; his pulse was full and regular, at seventy-five. At his request some milk and water was given to him; but, immediately upon drinking it, he vomited it up. Twenty leeches were applied to the abdomen without delay.

In the evening the symptoms became more alarming; the vomiting increased, and was accompanied with constant hiccup, which greatly distressed him, the pain in the belly was also

* Medical and Physical Journal, Feb. 1832.

much greater; he rolled about in the bed, was very irritable and restless; the anxiety of countenance was much increased, and the respirations were more frequent than natural: he did not complain when the abdomen was pressed with the hand. As he had not made water since his admission, I introduced a catheter, and drew off about ten ozs. of fluid, apparently chiefly blood, and directed that 15 more leeches should be applied to the belly.

Upon visiting him early the following morning, (Oct. 12th,) he said he was somewhat easier, but had passed a miserable, restless night, and had been greatly distressed by the constant vomiting; his countenance was still very anxious, and he appeared to be suffering a good deal from alarm and agitation. A catheter was introduced, and twelve ounces of bloody urine drawn off: this he said greatly relieved him. Towards night he became much worse; the belly was swollen and tense, and he complained of great pain upon the application of the hand; the vomiting was not abated, and it distressed him much more in consequence of the tenderness of the abdomen. It was found necessary to continue the use of the catheter, and the urine was deeply tinged with blood. Pulse small, sharp and regular, its frequency not increased; tongue natural; skin hot and dry; and he complained of great thirst, which could be only partially allayed by moistening his mouth, as every thing he swallowed was instantly rejected. ℞. Hydrarg. Submur., gr. ij.; Pulv. Opii, gr. ss. statim. Haust. Potassæ Citrat. quartâ quâque hora.

October 13th. Somewhat better; he has had some sleep during the night, and is greatly refreshed; the belly is less painful, and the swelling is diminished: he has passed about a pint of urine without the catheter. As his bowels were confined, he was ordered an enema, and it has produced a copious evacuation, which he says has made him feel much more comfortable. Pulse small, sharp, and much increased in frequency; tongue white and moist. V. S. ad ℥xij. To continue the medicines.

October 14th. He is greatly improved; his skin is cooler, and there is less irritability in the pulse; his bowels have been opened several times, and he passes his water naturally; he suffers very little. The blood drawn yesterday cupped and buffed. Ordered V. S. ad ℥x. Haust. Acetat. Ammon. c. Liq. Antim. Tart. ♏x. quartâ quâque hora.

October 16th. Since the day before yesterday he has been gradually getting better: the pain in the belly is almost gone, and the only thing he now complains of is great thirst and an uncomfortable fulness of the belly. The blood which was taken on the 14th exhibited no inflammatory appearance.

For several days after the last report, every thing appeared to be going on favourably, and he seemed to be getting well rapidly, when suddenly, on the evening of the 21st October, he became much worse; his skin again became hot and dry; his tongue parched and covered with a brown fur; and the anxiety of countenance, which was so apparent at first, had again appeared; he complained of great pain in the right lumbar region; the pulse was full and frequent, but regular. As his bowels had not been opened during the day, an enema was ordered, and he was directed to take the following powder every four hours: ℞. Pulv. Rhei, gr. v.; Hydrarg. c. Cretâ, gr. iij. M. fiat pulv.

On visiting him the following morning, (October 22d,) I found him in less pain than the preceding evening, but the other unfavourable symptoms were unabated. In the course of the day he had a severe shivering fit, which lasted for several minutes, and the pulse became very small and quick. Upon examining the belly, the part originally injured seemed to incline to the side, and appeared to contain fluid. He was directed to continue the medicines, and to have hot fomentations to the belly.

He continued in nearly the same condition during the two following days (October 23d and 24th,) appearing only to get a little weaker; but on the morning of the 26th, it was evident that there was no hope of saving him, as he was sinking rapidly. He complained of no pain in any part; he was perfectly sensible and collected, frequently saying that he had made up his mind to die; his countenance was sallow and very anxious, and he appeared to breathe with great difficulty; the pulse was too quick to be counted; the tongue was black and dry, and the skin hot, and without the slightest moisture. He fancied he should like some soda water, and it was accordingly given him, with small quantities of wine occasionally. At seven o'clock in the evening he died, having survived the accident just fourteen days.

The body was examined eighteen hours after death. Upon opening the cavity of the abdomen, the intestines appeared of a dark sooty colour, which was all that was remarkable in them. The whole of the solid viscera were in a healthy condition, with the exception of the right kidney, which, as had been anticipated, was found to be the seat of the mischief: by the injury it had been broken across just below the pelvis, and in it and the surrounding cellular membrane a large abscess was formed, which contained about three pints of fluid of a light brown colour: this was supposed to be pus, mixed with urine."

This is a very interesting case in several points of view. It displays the series of symptoms which might reasonably be expected to follow a rupture of the kidney, and may afford a hint on the treatment most consistent with facts and principles. The general symptoms, in the first instance, shewed that an important abdominal viscus was injured; the bloody urine told which that organ was. The earlier symptoms were restlessness, pain, not obviously and decisively increased by pressure on the abdomen, vomiting, hiccup. What did they indicate? Commencing inflammation of the peritoneum? No, for the symptoms occasioned by the latter, which, to instance the most analogous case, we see after ruptured liver, intestine, or bladder, differ in many respects from the preceding. They indicated the injection of the cellular membrane around the kidney with blood and urine, and the inflammation thereby induced in it. This inflammation was in some measure checked, but on the 10th day other symptoms were developed; pain in the lumbar region, anxious countenance, typhoid fever, rigor, the almost unerring indications of the formation of matter. He died, and urinous abscess was found to exist in the cellular texture. Here, then, we see that, in all injuries of parts surrounded by abundant cellular tissue, the great dangers to be apprehended are, the diffuse inflammation and the formation of matter, and that the latter may occur when the patient appears to be doing well, and the incautious practitioner is off his guard. We do not approve of the permission to the patient to drink milk and water immediately after the accident. If there is any one point on which we should be more particular than another, in cases of rupture or injury of the abdominal and pelvic viscera, it is the prohibition of both liquid and solid, food and physic, as far and as long as possible. We have had the treatment of many of these cases, and we were always most successful in proportion as we strictly adhered to this maxim. We always endeavour to make the lancet and leeches supersede all other means for the first two or three days.

Case 2. *Wound of the Throat, self-inflicted.*

John Row, æt. 30, a tailor, cut his throat with a razor early in the morning of the 5th March, 1829, and was immediately conveyed to the hospital. Mr. Laidlaw found him nearly dying from loss of blood. There was an extensive wound, reaching from immediately below the mastoid process, on the left side, to the opposite angle of the jaw.

" The left sterno-mastoid muscle was laid completely bare; the os hyoides and epiglottis were separated by the incision from the cartilages of the larynx; and the pharynx was so completely cut across, that the vertebræ of the neck were exposed, and could be seen through the gaping wound. There was no bleeding from any particular vessel at the time of his admission, but a general oozing of blood from the wound, which very soon stopped. A ligature was passed through the edges of the incision at both its extremities, and tied, and it was further supported by strapping. No suture could be made in front, as the patient's respiration was greatly impeded upon any attempt to close the wound more completely. The chin was brought to the breast, and there secured by a bandage passed round the head and under the arms, and he was supported in bed by means of a bed-chair.

He very soon rallied from the apparently dying condition in which I first saw him, and became calm and composed, making many attempts to speak, as if desirous of communicating something to us which he considered of importance: these attempts were, of course, unsuccessful. The day after his admission, he signified by signs that he could write on a board any thing he wished to ask us; and, upon being furnished with chalk, he inquired, with great apparent earnestness, if we did not think there was some hope of his ultimate recovery. He also complained of great thirst, which he endeavoured to allay by endeavouring to drink water, but which merely passed from the mouth out again at the wound: he said it afforded him very little relief. I explained to him the necessity of introducing a tube for the purpose of conveying food to the stomach, and he expressed his willingness to submit to it. On the third day an attempt was made to pass the gum elastic œsophagous tube through the mouth into the gullet, but, after several trials, it was found to create so much irritation, accompanied by fits of suffocation, that it was thought most prudent to desist for the present.

He thus continued, without apparently getting either better or worse, for two days longer: the only thing which he complained of was the excessive thirst, which he still attempted to allay by taking water into the mouth, which instantly flowed through the wound. In this way he consumed several gallons of water daily, and, if left for a moment without a supply of it, he became greatly excited and distressed. On asking him if he suffered from want of food, he wrote on the board that he did not, and that he thought he could do without any for several days longer.

Upon the fifth day after his admission, an attempt was again made to pass a tube into the gullet, which, after a few trials, was attended with success, and a small quantity of beef-tea was thrown into the stomach; it produced a slight degree of nausea, and the tube was withdrawn. In the evening the tube was again passed, and a little more broth was injected. He explained by writing that he did not feel any better for the nourishment he had received, but, on the contrary, rather exhausted. He complained of cold, and requested to have some more bedding, and was very anxious to be allowed to get up, and sit by the fire.

During the night he had a little sleep, and appeared more comfortable than usual; but at an early hour the following morning the nurse found him dying, and before I could be called he had expired.

The body was examined on the following day. Upon examining the wound in the throat, it was found that the main trunks of all the arteries of the neck had escaped injury, but the incision was within a line of the lingual artery and nerve on both sides. The epiglottis was divided transversely, and a portion of it remained attached to the root of the tongue; the pharynx had fallen down or retracted, when divided, so as to be below the incision. The viscera of the thorax were perfectly healthy: in the abdomen, the small intestines were found quite empty, and seemed shrunk: in the lower part of the colon and rectum, a small portion of hardened fæces remained; in the right lobe of the liver, two immense cavities were found filled with fluid, apparently scrofulous matter mixed with bile, and several large hydatids. The largest of these abscesses was situated towards the convex surface of the liver, and was pointing through the diaphragm; it appeared to have been formed by the union of two smaller ones, as there was the remains of a septum in the centre; the walls were thick, and had a cartilaginous feel. The smaller abscess contained several hydatids of a much larger size than those in the other abscess; there was also a greater proportion of bile. It is singular that disease to such an enormous extent should have existed so long as this necessarily must have done, without it being ever suspected that he had any internal complaint, as his friends assured me was the case. The larger abscess would, no doubt, in a very short period have emptied itself in the cavity of the chest, and he would most probably have died from empyema."

This appears a remarkable instance of the extent to which visceral disease may proceed, without giving rise to actual feelings of illness on the patient's part. We do not think that the secondary effects of suicidal wounds of the throat have received sufficient attention. We possess the particulars of some cases, which we shall probably detail in another part of this Journal.

---

### XX.

The Cyclopædia of Practical Medicine. Part III. Price 5s. London, Sherwood and Co.

The late period of publication of this Part of the Cyclopædia of Medicine will necessarily prevent our taking any thing like a due notice of it. An additional sheet has been added this month, and in future this addition will continue to be made, which we think is highly judicious on the part of the proprietors. As some of our contemporaries remarked, we think that the price was previously too great. We are happy to find, what indeed we entertained no doubt would be the case, that the sale has been such as to offer every encouragement to those engaged in its production. One of the best articles of the present number is that on inflammation of the brain, from the pens of Drs. Quain and Adair Crawford. It occupies 34 columns, and is very creditable to the research and judgment of those gentlemen. We cannot pretend to present a complete account of the article in question, but we may offer a specimen or two of the manner in which it is executed, and convey some useful information in a few separate quotations.

Whilst the authors communicate all that is certainly known on the diagnostic characters of inflammation of the membranes and substance of the brain, or of *meningitis* and *cerebritis*, they very properly acknowledge that in practice those diagnostic signs are at present too uncertain, to admit of our hazarding positive opinions on such a foundation. The anatomical character of the circulating system of the brain and its membranes makes it probable, that inflammation of the one becomes complicated too soon with inflammation of the other, to allow of very nice discrimination between them. Practically such distinction is rendered comparatively unimportant by the very circumstance or series of circumstances that render it difficult, and it is of greater consequence to determine with accuracy what are the symptoms which indicate inflammation within the cranium, than nicely to mark its exact situation. The following passage by Dr. Quain on the causes of meningitis is worth transcription; after noticing its frequency and insidiousness after injuries of the head, he goes on to remark.

"In the list of causes should also be included depressing passions; sudden fright, or unexpected disappointment; violent emotions; immoderate indulgence of any sort, particularly in the use of spirituous liquors; the suppression of cutaneous eruptions, and habitual evacuations or discharges; insolation *(coup de soleil)* in warm climates; deposition of bone in the dura mater; tubercle developed in the cerebral substance, and extending to the circumference of the hemisphere, so as to become a mechanical irritant to the membranes.

Such are the exciting causes of meningitis; but there are predisposing circumstances which confessedly render some persons more liable to it than others. One class of persons consists of those who possess what is termed the apoplectic constitution, indicated by a large head, short neck, and sanguineous temperament; the other class exhibits evidences of the lymphatic temperament, together with considerable cerebral development, indicated by precocious talent. Such persons in early life are very liable to attacks of meningitis, which terminate in serous effusion. Sex also seems to be a predisposing cause, or rather the peculiar pursuits and habits of the sexes; for males are more liable than females, according to Martinet, in the proportion of three to one. All ages are liable to this affection, but not equally so; of one hundred and sixteen cases recorded by the author just cited, twenty-nine were under fifteen years, forty-four from fifteen to thirty, thirty-eight from thirty to sixty, and five from sixty to eighty. These results, it should be remembered, are taken from hospital practice, in which the great majority of patients are adults. In private practice the proportion of cases under puberty is much greater than that here stated. The ordinary duration of acute meningitis is from seven to ten days, but death may occur on the third or fourth; few cases are prolonged beyond the twenty-fifth or thirtieth."

*Cerebritis.* This is divided by Dr. Crawford into general and partial, acute and chonic. Inflammation of the brain sometimes commences suddenly, but usually it is preceded by premonitory symptoms, with which it is highly necessary that the practitioner should be familiar.

"The most usual *premonitory* symptoms are, a general uneasiness and restlessness, with a tendency to congestion in the head; a sense of weight and fulness; occasional attacks of pain in the head, or of temporary apoplexy or epilepsy; flushing of the face and increased heat of the head; drowsiness and vertigo; preternatural acuteness of the external senses; intolerance of light and optical illusions; contraction of the pupils, strabismus, or imperfection of sight; tinnitus aurium, or various other noises in the ear; confusion of thought; failure of the memory; mental excitement or depression, or some striking alteration in the habitual character and pursuits of the individual. In some cases there is little appearance of indisposition throughout the day, but the symptoms are aggravated at night: the sleep is uneasy or disturbed by alarming dreams, and in children there is often grinding of the teeth. Pains in the limbs and frequent cramps, general lassitude and muscular debility, are often felt, alternating with fits of shivering and feverishness; the digestive functions are disordered; there is in general loss of appetite; often obstinate vomiting; the bowels are either irritable or torpid, the secretions being always unhealthy.

The above symptoms precede either general or partial inflammation of the brain. Those which follow indicate more especially the invasion of partial and chronic inflammation. A long-continued, fixed, and deep-seated pain in one part of the head; pain, numbness, weakness, a sensation of creeping and tingling in one extremity or in one half the body; or confined to one portion of the extremity: there may be numbness and loss of power in one finger only, or in one set of muscles. Sometimes the speech is affected so as to produce a degree of hesitation, stuttering, or indistinctness of pronunciation. Drowsiness, languor, depression of spirits, are observed also more particularly in the chronic form of cerebritis. Some of these premonitory symptoms may have been present for weeks, for months, or even for a year, or for a longer period. The practitioner cannot be too deeply impressed with the importance of the discovery and due appreciation of the foregoing train of symptoms: many of them may be overlooked by the patient, unless his attention be called to them; he attributes his ailments to nervousness, debility, rheumatism, gout, and any but the real cause. He is allowed, perhaps, to live well; his stomach is strengthened and his nerves are soothed; whilst the disease in the brain, which will certainly end in death, is suffered to pursue its course."

We must pass over a good description of acute and partial cerebritis, contenting ourselves with this short summary of the symptoms of either.

"When the inflammation occupies at the onset a large portion of the brain, we have seen that it is generally complicated with meningitis, and characterized by disturbance in all the vital functions; that we may recognise in the symptoms a period of high excitement, and one of corresponding depression; and that it runs its course speedily, either to a favourable or fatal termination.

When, however, a smaller portion of the brain is engaged in inflammation, as in *partial cerebritis*, the course of the disease is seldom so rapid; its invasion is more gradual, and preceded by symptoms of irritation in some of the organs of voluntary motion, sensation, or intelligence; and in the more advanced periods of the disease the functions of some of these organs become considerably impaired, or completely abolished."

We think there is much reason in the distinction shewn by Dr. Crawford between the state of brain attending convulsions and that occasioning paralysis. In practice we too often see the distinction overlooked, and convulsions considered as certainly indicative of pressure to some amount on the brain. We remember seeing a patient labouring under erysipelas trephined on account of convulsions. Nothing was discovered. Fortunately the patient recovered.

"The convulsions, paralysis, and other pathognomonic symptoms of cerebral inflammation, may be referred to the more immediate action of three causes:—1st, *inflammation* affecting either the membranes or a portion of the substance of the brain: 2d, *nervous irritation* extending from the diseased to the healthy parts of the brain, and stimulating its functions: 3d, *pressure*. The difficulty in ascertaining from which of these causes the various symptoms arise, constitutes the chief obstacle to our always forming an accurate diagnosis in cerebral diseases. Convulsions, spasmodic paralysis, increased activity of the intellectual faculties and external senses, delirium, and all symptoms of excitement, may generally be considered to indicate either commencing inflammation or nervous irritation in the substance of the brain or its membranes. Convulsions may also arise from pressure alone, although paralysis is its most frequent effect; but the absence of all fever and other symptoms of general irritation, will distinguish these convulsions from those of arachnitis and cerebritis: the same distinction applies to the convulsions of a purely nervous character, observed in hysteria, and after the use of certain poisons, &c.

The effects of pressure have often been examined by experiments on animals. If the cranium of a dog is trepanned and pressure performed on the dura mater to a certain extent, the animal shews signs of great uneasiness, and is affected with general convulsions; if the pressure is increased, the convulsions cease, the breathing becomes stertorous, the animal torpid and comatose; if the pressure is diminished, the breathing becomes more free and the convulsions return; and if it is entirely removed, the animal soon completely recovers. The principal causes of pressure are congestion; effusion of blood, pus, or serosity into the ventricles, between the membranes, or in the substance of the brain; organic diseases of the brain or cranium. The obscurity in which all these interesting questions is still involved, is capable, we think, of being considerably lessened by further investigation; and nothing would be more likely to contribute to so desirable an end, than pathological observations made with care and impartiality. A valuable collection of cases illustrating the consequence of pressure on the brain, will be found in Dr. Bright's Reports of Medical Cases."

We have a good account of the morbid anatomy of cerebritis; and Dr. Crawford very properly remonstrates against the too common practice of setting down, as decided inflammation, every appearance of redness and increased vascularity. Under this head, we must content ourselves with a passage on that rare occurrence, ulceration of the brain. We have heard a surgeon, now in the greatest practice in London, declare that he never saw but one instance of it. We have never had an opportunity of observing it.

"*Ulceration.*—This state of the brain has been described by several writers, although it is uncommon. It has been found on the surface of the convolutions, on the optic thalami, and the corpora striata. The ulcers present an irregular surface, covered partially with either a bloody or albuminous exudation, and having jagged edges. In some cases it is hard and dry; in others there are fistulous communications between the ulcer and deep-seated collections of pus; and in others the ulcer forms the bed of a coagulum of blood: the ulcers vary in depth and dimensions. Some writers consider these ulcerations as affecting more particularly the arachnoid and pia mater, and not belonging to the cerebral substance. This may be true in some cases, but not in all. Morgagni speaks, in one of his cases, of an erosion of the corpus striatum, and in another of an ulcerous cavity at the base of the left ventricle. Two cases of the same nature are recorded by Scoutetten.

There are also ulcerations occasioned by the penetration of foreign bodies, of which many cases are given by Morgagni and other surgical writers, but these are foreign to our present purpose."

The periods of life most exposed to cerebral inflammation, are old age and childhood.

"The examination of a large number of cases shews that more than two-thirds were persons above forty-five, and a majority of these between fifty-five and seventy. It is a disease, however, which may affect all ages. The frequency of falls and blows on the head in children, the irritation of dentition, the pressure of the head during parturition, predispose them to cerebritis, although there is perhaps a greater tendency at that period of life to membranous inflammation. *Traces of cerebritis have been found in still-born children* (?) Men are more subject to cerebritis than women, in the proportion of about two-thirds, owing probably to their being more exposed to the influence of exciting causes. The disease, also, is more common in hot than cold climates, and in Summer than Winter."

Perhaps it may admit of question, whether cerebritis occurs in the fœtus, as here stated. That in still-born children, a condition of brain not perfectly natural may have been observed is very likely, and not difficult of explanation; but that inflammatory action has really occurred, is more than our present imperfect acquaintance with the nature of softening, and some other changes of the cerebral substance, can warrant us in definitively pronouncing.

The last consideration that will occupy us, is the treatment of inflammation of the brain. That of the acute form is generally well conducted; but the management of the chronic requires more knowledge, discernment, and discrimination.

"Cerebritis and arachnitis are so formidable, that their prevention is of no less, if not of greater importance, than their treatment. It is extremely important to have recourse to active measures on the very first appearance of any of the premonitory symptoms, however trifling: as we may thus succeed in effectually removing that state of congestion and irritation of the brain which is the precursor of inflammation. The means of accomplishing this are, the careful and timely removal of all the exciting causes; of every source of irritation, both bodily and mental; regulation of the diet; avoiding all excesses; relaxation from study, change of air, general and local bloodletting, counter-irritation, with occasional purgatives. Great vigilance is particularly required in the cerebral affections of infants and children, who frequently suffer without complaining. A predisposition to affection of the brain may often be suspected by some slight cast or rolling of the eyes; by dilatation of the pupils; or occasional startings, and attacks of spasmodic croupy breathing during sleep: there may be every other appearance of perfect health with these symptoms, which are often only evanescent; but they will not escape the attention of the experienced observer, and will be sufficient to put him on his guard."

Dr. Crawford recommends the application of leeches to the anus, or bleeding from the feet or legs, immersed in a hot bath. The daily use of the cold shower-bath is very useful, particularly if the feet are, at the same time, kept immersed in hot water. Dr. Crawford seems to us to speak too lightly of mercury; he gives it as a purgative only. We have seen too many instances in which its specific action seemed highly beneficial, to look at it in so limited a view as Dr. Crawford. The methodus medendi is judiciously laid down in all other respects by our author; but we need not pursue the items further. The article is altogether very creditable to its authors, and a favourable sample of the scope and character of the work. We would recommend its able editors to allow sufficient space to the consideration of subjects of practical utility, and to curtail others, which have no such commanding claims on our respect.

XXI.

I. Operation for Imperforate Vagina.*

Dr. Stedman, of St. Thomas, West Indies, has related a curious case of this description. The patient was a young negress, 16 years of age. Between the labia there was no fissure whatever, except near the orifice of the urethra, where there was a small round hole, about the size of a large pin's head. There was not the slightest vestige of an opening into the vagina, which appeared to be closed up by a thick, firm substance, covered with skin resembling that of other parts of the body. The aperture through which the urine passed being casually enlarged by ulceration, Dr. S. was enabled to introduce a female catheter, which drew off a large quantity of fluid of an urinous smell, mixed with a thin reddish liquid, which appeared, indeed proved, to be the menstrual discharge. The catheter could be depressed nearly in a perpendicular direction behind the septum, closing the vagina, when a larger quantity of menstrual fluid issued. After some accidental complications had been got rid of, Dr. Stedman, with the assistance of several other gentlemen, performed an operation for the girl's relief from her infirmity.

She was placed on a table, in the usual lithotomy position, when Dr. S. having ascertained with the finger per anum, that there was no connexion between the vagina and rectum, introduced with some difficulty a small grooved director, in a perpendicular direction, through the aperture already mentioned. He then made three rapid incisions with a small scalpel through the septum and upon the director, dividing thereby the septum, which was about a quarter of an inch in thickness, and of a membrano-ligamentous structure. The finger was then introduced, and the interior of the vagina found natural in its structure. After the operation, a piece of lint, dipped in olive oil, was introduced between the lips of the wound into the vagina, a compress of lint applied, and all kept in place by a T bandage. A female catheter was also kept in the urethra. The patient is now perfectly recovered, having a vagina of a natural appearance. Dr. Stedman has no doubt that the closure of the vagina took place when the girl was an infant, and was caused by excoriations and subsequent union between the nymphæ, union which subsequently assumed the firmness and structure described. Some time ago, Dr. S. was called to see a female mulatto, two years of age, in which the nymphæ were beginning to adhere. Medical aid being called in time, the adhesions which extended along the whole orifice of the vagina were slight, and were easily separated

* Edinburgh Journal, No. CX.

by the introduction of a silver probe, while the parts were subsequently prevented from adhering by pledgets of lint dipped in a weak solution of alum. It is obvious that cases of this kind, in which the union, though extensive in surface, is not deep, are more easily remedied by operation and more permanently benefitted than those, in which the bond of union extends for some depth into the vagina.

### II. Extirpation of a Tumour in the Situation of the Parotid Gland, preceded ay Ligature of the Carotid Artery.

There cannot be a more gratifying proof of the progress of knowledge than is exhibited every day by the periodical annals of our own science. Scarce a medical journal sees the light, that does not contain the records of important operations performed by provincial and colonial surgeons. They now vie in zeal, in learning, and in talents, with our metropolitan confrères, and if they have not always the same experience and consequently caution to direct them, it is not their fault, but their misfortune. We have always great pleasure in contributing to disseminate the achievements, for achievements they really are, of our zealous brethren in the country and in our foreign possessions. We therefore more willingly publish the ensuing case.

John Sensire, a free-black, æt. 58, a boat-man, applied to Dr. Stedman in the latter end of August, 1830, on account of a large tumour on the right side of the neck and jaw. It extended from behind the concha of the external ear to an inch below the angle of the jaw-bone; upon the upper part, the tip of the ear, and part of the cartilage of the concha, were imbedded in the tumour, which extended on the fore-part from a little below the malar bone to the upper portion of the thyroid cartilage. It dipped under the jaw-bone to the depth of nearly two inches. The greater part was of stony hardness. but two lobes on the top of the tumour were softer, with thin blue skin, which in a few days ulcerated, and gave issue to a thin and fetid humour. The disease had commenced twelve years previously at the angle of the jaw, and had increased with unusual rapidity during the last year, when it was attended with much pain. The patient's constitution appeared robust. Dr. Stedman determined to remove the tumour, previously tying the right common carotid artery. The operation was performed on the 7th September in the presence of Baron Von Bretton, M.D. Dr. Hornbeck, and Dr. Ravn, garrison-surgeon, assisted by three persons, to secure the patient. We cannot satisfactorily abridge the record of the operation, or rather operations; shorn of their difficulties in narration, the narrative itself ceases to be instructive.

"The man being laid on his back on a firm cot, and his chin turned to the left side, an incision was made along the internal margin of the *sterno-cleido mastoideus*, commencing from a point on a line with the middle of the thyroid cartilage, and extending to near the sternal extremity of the clavicle. The fibres of the *platysma myoides* were next cut through, and I proceeded cautiously to dissect through the cellular substance to find the sheath of the artery. This part of the operation was rendered very embarrassing by the quantity of blood, both venous and arterial, that flowed at each touch of the knife, for as fast as it was sponged, the wound was filled up again with blood. As the bleeding continued, notwithstanding five small arteries had been tried, by the advice of Dr. Hornbeck, I enlarged the incision backwards towards the tumour. This was a circumstance of little moment, as this incision would have been necessary, at all events, in the subsequent operation. This gave me more room; and by waiting patiently for a short time, the wound was so far cleared of blood, as to allow me to distinguish the sheath of the carotid artery, with the *descendens noni* on its anterior part. The artery lay very deep, so that after repeated attempts to cut open the sheath, I was obliged to make the man sit up. On turning his chin to the left side, the artery now became much more superficial; and I was easily enabled to open the sheath, by pinching up a part of it with the forceps, and dividing it, by cutting with the knife laid flat. Having passed the common aneurismal needle, armed with one stout well-waxed silk thread, closely round the artery, and having satisfied the gentlemen in attendance that there was nothing but the artery included, I drew the ligature firmly and strongly. The pulsation immediately ceased in the upper portion of the artery, which I could distinctly feel through its sheath for at least a third of an inch above the place where it was tied. The internal jugular vein gave me not the least embarrassment, nor did I even see it during the operation. The artery was tied at a point about a line opposite to the middle of the thyroid cartilage.

This first operation lasted fifty-five minutes, and would probably have been concluded in half the time, but for the embarrassment caused by the superabundant flow of blood.

The patient was now allowed to rest for a quarter of an hour. A cup of chicken-soup, and a wine-glass of a cordial mixture, composed of tincture of lavender, essence of peppermint, &c. were given him.

The patient being again fixed in a recumbent position, I proceeded to extirpate the tumour.

An incision was made from behind the *concha* of the ear to the termination of the tumour in the neck. I next dissected the skin from the tumour until I had arrived near its base. An oval incision was then made on the front part of the tumour, extending from the front of the ear to the termination of the first incision. I was unable to save so much skin as I had wished, from its being tuberculated, and of a suspicious appearance. The tumour was dissected out cautiously on this side also. Notwithstanding the ligature of the common carotid artery, several arteries sprung in the course of the dissection, so that I had altogether to tie seven in this part of the operation. The tip of the ear, and the cartilage above, were now dissected out of the tumour, which was dissected alternately on each side, until it hung by a portion not thicker than the middle finger, deep under the angle of the jaw. Upon attempting to cut this, a considerable artery jetted out its blood. Drs. Hornbeck and Ravn having thrown two ligatures round this part, I boldly cut it away, and had the satisfaction to find that no hæmorrhage followed. A small lymphatic gland, about the size of a bean, being a little hard, was seized with the hook and extirpated. The whole of the space that had been occupied by the tumour was now carefully searched by the attendant physicians and myself, and no diseased portion could be detected. In the course of the dissection, I was obliged to cut away a part of the head of the sterno-mastoid muscle, as it was impossible to separate them.

The wound that was exhibited after the removal of the tumour, including the one made for tying the carotid artery, extended from the mastoid process of the temporal bone to near the sternal extremity of the clavicle, and from the front part of the ears to deep under the jaw, near the angle of the mouth. The lips of the wound from the sternal extremity, to the angle where the two incisions round the tumour met below the jaw, were brought together by the interrupted suture.

The upper wound, where the tumour was situate, could not be closed by the skin, as a large portion had been cut off for its unsoundness. This space was therefore covered with adhesive strap, and allowed to granulate.

Slips of adhesive plaster were put between the stitches on the wound on the neck, and the whole being covered with a layer of charpie, a loose roller was passed round the neck, and over the top of the head.

The second operation lasted about 48 minutes. The patient did not lose above fifteen ounces of blood; he was not the least exhausted; and at the conclusion of the operation, I found his pulse exactly 96."

The patient bore the operation extremely well. The tumour weighed 1lb. 12 oz.; was ulcerated at the summit, was of scirrhous structure, and one of the lobes at the base contained fetid purulent fluid. We need not pursue the subsequent details. On the 10th the whole of the dressings were removed, and the wound on the neck was found to have healed from the lower part up to the last ligature in the skin. The upper part, where the tumour had been, was much contracted, and the granulations were healthy. The wound was dressed daily, all the ligatures separated, the last, that on the carotid, coming away on the 3d October. On the 6th he was dismissed perfectly cured, with a slight contraction of the mouth to the left side. It is now a year since the performance of the operation, and the man continues in uninterrupted good health.

Dr. Stedman believes, what most men of information believe also, that complete extirpation of the parotid gland is, if not impossible, next to it, and has probably never been accomplished: it certainly was not in the present instance. The operation was a fortunate one, and appears to support the idea that negroes recover with more facility than Europeans from formidable mutilations.

---

### XXII.

### Case of Cholera at Banbury.

*Lamb's Conduit-st., March* 16, 1832.

Dear Sir,—I consider the case I mentioned this morning and now enclose, of such great importance, in clearly showing that a disease *identical* with that now prevailing in London, may arise 60 miles or more therefrom, without a shadow of evidence of its having been imported, as will be perceived on perusal of the case admirably drawn up by the surgeon in whose practice it occurred, that I beg you will give it insertion in your valuable Journal.

J. Clarke.

Dear Sir,—I am happy in having it in my power to give you the information you wish, respecting the case of cholera, being, as you observe, the first person that saw the patient, and, in fact, his nurse for nearly two hours subsequently. I visited him about half-past 12, and the symptoms being so entirely different from any thing I had before seen, and agreeing so exactly with the descriptions of cholera I immediately suspected this to be a case, and, after giving directions, returned for medicine, and within five minutes the patient was seen by Mr. T. Brayne according to my request. I shall now describe the symptoms from the statement reported to the Banbury Board of Health, by Mr. T. B. who with myself were his principal attendants. We found him in bed, his face had a livid purple hue, particularly *the lips*, his *eyes sunk*, glassy, and only half covered by the eyelid, and his whole countenance *anxious* and depressed; he spoke in a *peculiar low whisper*, and could only say that he felt extremely weak, his *face was cold*, nose sharp and contracted, tongue white and clammy, felt also cold to the touch; his breathing was quick and laborious, and the exhaled air from the lungs felt *cold* to the hand when held before his mouth; the surface of the whole body was *cold* and clammy, but nearly of a natural colour, and the *pulse could not be felt at the wrist*. He complained most of *cramps* in his *belly, thighs, legs, feet, arms, hands, and back*, particularly when he moved, which he did every now and then to make slight efforts to retch, and then fell back *exhausted* on the bed —also he complained of *great oppression at his chest*, and pain at the *pit of the stomach*, which pressure increased. His wife said that he had had at least *twenty stools* within the last two hours, and that they now ran from him involuntary; they resembled rice gruel, or something white—had been poured into water—slightly tinged with blood, and subsided in a flocculent form at the bottom of the vessel. He had *passed no urine* since the morning; something warm had been given and immediately rejected. Three tea-spoonsful of mustard in half a pint of water were immediately given him, and after the second half of this he vomited copiously and violently. Tin cases and bottles of hot water were applied to as much of the surface as possible, with the addition of more clothes;—after the vomiting from the emetic had subsided, 60 drops of tr. opii in a glass of *strong* hot brandy and water were next given, and part was rejected—the temperature and colour of his face somewhat improved. In the course of two hours he took nearly a pint of brandy with hot water, and two more doses of laudanum; and by the end of that time considerable improvement had taken place in the warmth of surface; the pulse now began to be perceptible, and the cramps and breathing were better. Two grains of calomel with one-third of a grain of opium were given every hour till 7, p.m. at which time he was rather hot, except *the feet*, face changed from purple to red, pulse 120 and full, and the cramps, the legs excepted, much abated; in short, re-action had taken place. Nine, p.m. Improving, has passed three stools, mouth very clammy; *thirst, and a wish for cold drink, which he did throughout;* speech still low—effervescing saline med. with an excess of alkali; test. pp. and minute doses of opium every three hours; diluent drinks, and discontinuance of all stimulants. March 9th. Has had a little sleep, been warm and perspiring through the night—pulse full, countenance natural, has had three slimy stools, *no urine* has been made since the attack, and *there is none in the bladder*. Six, p.m. A comfortable day—two stools, has passed a little urine. Sunday evening, going on well, diarrhœa ceased; no stool since Friday, cramps gone, pulse 88, skin moist, urine in sufficient quantity, complains of weakness, every thing promises certain and speedy recovery. Having related the symptoms, I shall now describe his character. His name is J. Turbitt, æt. 29, works for the Commissioners of Roads, in sweeping, cleansing, &c.—has eaten nothing particularly offensive of late; was employed cleansing a sewer Monday and Tuesday, both very wet days, when he got wet through, and rather inebriated in the evening—rather addicted to drinking, and night-poaching for fish. He had three stools on the Wednesday, and some time since diarrhœa. He got up well on the morning of his attack, (Thursday); soon after six came home to breakfast between eight and nine, and went to work again till half-past ten, when he returned, complaining of having been *very sick, with a purging*. These symptoms, with *cramps* and *coldness*, continued rapidly increasing till noon, when he applied for advice. In November three of his children had typhus fever, the youngest died; the leading symptoms were excessive diarrhœa. Neither he nor his wife were ill. Two other children, living in the same yard, about the same time, had fever; these being the only cases in that part of the town, it being very prevalent elsewhere. We have had no case of fever now for several weeks. The yard he lives in is very dirty and confined, his house comfortless, and he had great deficiency in *bedding, clothes*, &c.

☞ We have, unfortunately, lost the name of the writer, while transcribing the case from the letter to Mr. Clarke. —*Ed.*

---

### XXIII.

### Dr. Yates' Letter.

SIR,—I take the liberty of troubling you with a communication which I have received from Vienna on the subject of the Cholera, which may perhaps be worth your reading, as it contains the opinions of two gentlemen who have had considerable experience during the prevalence of the late epidemic, at the Austrian capital. I showed the original to Dr. Babington of the City, who, I believe, is one of the Board of Health. He considered the paper of sufficient importance to be worthy of a place in the CHOLERA GAZETTE, and, accordingly, introduced me to Dr. Russell, who expressed himself desirous of having it;—he said indeed he should be "personally obliged to me for it, and that the Board would also be grateful." I was thus induced to translate it; but, on calling afterwards on Dr. Russell, I was surprised to find that he objected to give the paper a place in the Gazette, saying that, "as the author set out upon the principle that the disease is *not contagious*, it would be quite contrary to the *views of the Government*, or indeed of the *gazette itself*, and entirely counteract their *designs*. I told him I was not aware that they had any particular motive in view, or that they wished to consider one side of the question more than the other; but that (supposing truth to be the object) they would be happy to receive any information which was founded on *experience*, and likely to benefit *mankind*.

W. Holt Yates, M.D.*

---

### XXIV.

### Remedy for Ophthalmia discovered by Dr. Seeds, R.N.

This old and zealous physician, whilst labouring under an obstinate attack of ophthalmia, determined to try the effect of an external application. It removed the inflammation in his eye with great rapidity, and believing that what was of service to himself, would also prove serviceable to others under similar circumstances, he determined to publish the formula of his preparation. Mr. Guthrie, with his usual affability and good feeling, allowed Dr. Seeds to try the effects of the application at the Ophthalmic Infirmary in Chandos Street. The results we believe were satisfactory. The following is the formula, with the mode of using it.

℞ Sp. aeth. sulph. c. ℥i.

Sp. ammon. comp. ℥i.

Sp. vini camph. ʒi. M.

This lotion is applied over the eyelids, forehead, and temples, in acute and chronic ophthalmia; and also introduced into the nostril with the point of the finger.

We hope that Dr. Seeds will be gratified by the success of his remedy, as we have reason to believe that it has been already very great.

---

### XXV.

### Dr. Clarke's Algid Fever of Smyrna, compared with Dr. Barry's Blue Cholera.

Sir,—In the October Number (1826) of the Medico-Chirurgical Review, there is published a paper written by Dr. Clarke, descriptive of a strange disease that was epidemic at Smyrna, in 1825-6, and which bears so striking a likeness to the worst cases of blue cholera, that the following passage may be worth transcribing at the present moment.

"There is no shivering, nor is the slightest sensation of cold complained of. The pulse, if felt at all, is thready and rapid, disappearing under the slightest pressure of the finger. There is, more usually, a total want of pulsation in all tangible vessels, and even in the region of the heart. The countenance, in some cases, is livid, and betrays unspeakable anguish. The features are withered and shrunk. The intellectual powers are, for the most part, but little impaired; and there is frequently a command of muscular exertion, a calm composure and self-possession which would lead any one, unacquainted with the patient's situation, to believe him free from complaint. But when the fingers are applied to the wrist, and the pulse is sought in vain—when the more than deadly coldness of the surface is perceived, the illusion vanishes. In this state of equivocal existence, he continues from ten to eighteen or twenty-four hours, and, in some cases, much longer, when, if he survives the paroxysm by the persevering application of remedies, and the re-active energies of the system, an increase of temperature takes place, which gradually acquires different degrees of intensity, and occasionally requires to be moderated."—Medicus.

The above description is so perfectly identical with some of the very *worst forms* of the present epidemic, that we think it is well worthy of a place here.

---

### XXVI.

### Dr. O'Halloran on the Epidemic Cholera.

*To Dr. Johnson,*

My dear Sir—I visited the cholera hospital at Southwark, and I do not hesitate in giving it as my opinion, that the disease which prevails in that quarter is not cholera. I am induced to form this conclusion from its identity with many of the cases of epidemic fever which I

---

* We regret that we cannot in this number find room for the German Paper. It will certainly appear in our next. *Ed.*

have seen in the different towns of the Mediterranean and West Indies, where the character of the common endemic of the country is remarkably changed by the operation of an epidemic cause, and where the same exhaustion—the same sinking of the pulse—the same shrinking of the features—the sinking of the eyes—the deadly coldness of the extremities—the malignant aspect—the insatiable thirst—the vomiting, retching, griping and spasms—the pain in the hypochondria and calves of the legs—the continual drowsiness, restlessness, with the peculiarities of the tongue, voice, and urinary organs, were remarkable amongst the poor in the ill-ventilated houses in filthy lanes and alleys.

Moreover, the cholera of the East is seldom preceded by diarrhœa—in the epidemic of London, neglected diarrhœas drain the system, in a great measure of its blood, and the frightful termination, misnamed cholera, is the effect of the absence of the vital fluid. In the malignant epidemic diarrhœa of London, where reaction and excitement are produced by the prompt application of remedies, the character changes, and the disease assumes the typhoid type—conversions unusual in the cholera of the East.

The cholera of the East strikes suddenly, and the termination in recovery or death takes place within a short period—in the epidemic in London, the period is protracted to an indefinite time.

In the East, whole communities are sometimes seized suddenly by epidemic cholera—in London the epidemic chiefly confines itself to the wretched inhabitants of filthy streets and ill-ventilated houses.

Much importance seems to be attached to the state of the urinary bladder after death, considered by some as diagnostic of cholera; but, such is its condition generally, in all malignant fevers; for, as the urinary secretion is suspended, the organ contracts so as to be incapable of containing an ounce of fluid; and this, not the effect of the spasms and cramps, as they will have it, accounts for the diminution of the organ, as the deficiency of glandulous secretions about the mouth and neck, together with the collapsed state of the lungs, explains the peculiarity in the voice and its occasional loss.

A similar degree of consequence is attached to the absence of the pulse, and to the appearance of the extremities before death. There is nothing unusual in the appearance so much insisted on as peculiar to this disease, for we find, in all malignant congestive fevers of tropical climates, that as soon as the circulation deserts the surface and extremities, the latter turn blue; and cases are on record, where the feet assumed the appearance of putrefaction before life was extinct—a condition which I witnessed in the hospital at Xeriz, in the epidemic of 1820.

The only peculiarity which I witnessed in this disease, consisted in the nature of the stools; but as the intestines are cleared of the morbid contents in the early stage, it is not to be wondered at that the evacuations should resemble and consist of, in a great measure, the fluid taken into the stomach after secretion and absorption are at an end. T. O'HALLORAN, M.D.

*London, 17th March,* 1832.

---

We have received a pamphlet from the pen of Dr. Reich, of Berlin, detailing most extraordinary success in the treatment of the late epidemic in that capital. We have no room for details. He considers the poison which produces the disease called cholera to be one generated in the stomach and bowels, not received *ab externo.* His plan of treatment was this. Dissolve ten grs. of emetic tartar in seven ozs. of water, and give half an ounce every two hours, continuing the medicine, even after the evacuations have ceased, till appetite and natural fæces are restored. The first effects are, more copious purging than vomiting; but these soon cease, *tolerance* of medicine being, in a short time, established. He allows the patient plenty of cold drink—prohibits all heat and frictions—gives no other internal remedy—and states that, by the above measure, he cured 119 out of 123 patients.

Considering the fatality of the disease in its asphyxial stage, we seriously recommend a trial of the plan.

